# Reversible Redox Ligand-Centered Reactivity in 2,6-Bisiminopyridine Aluminum Systems

**DOI:** 10.1021/acs.inorgchem.4c02664

**Published:** 2024-10-04

**Authors:** Juan Manuel Delgado-Collado, Hellen Videa, Pablo J. Serrano-Laguna, M. Ángeles Fuentes, Eleuterio Álvarez, Antonio Díaz Quintana, Antonio J. Martínez-Martínez, Antonio Rodríguez-Delgado, Juan Cámpora

**Affiliations:** † Instituto de Investigaciones Químicas, CSIC-Universidad de Sevilla. Av. Américo Vespucio, 49, Sevilla 41092, Spain; ‡ CIQSO-Center for Research in Sustainable Chemistry and Department of Chemistry, CSIC-Associated Unit, University of Huelva, Campus El Carmen, Huelva 21007, Spain

## Abstract

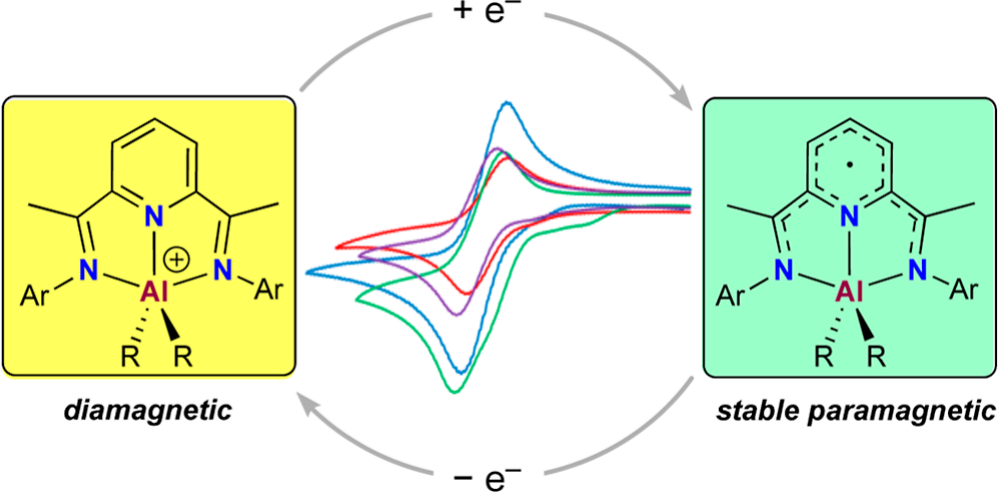

We report the synthesis of cationic 2,6-bisiminopyridine organoaluminum complexes, [(BIP)AlR_2_]^+^, as stable BAr^F^_4_^–^ or PF_6_^–^ salts, and their reversible single-electron reduction into well-defined paramagnetic species, [(BIP·)AlR_2_]. Four redox couples, [(BIP)AlR_2_]^+/0^, have been fully characterized through structural, spectroscopic, electrochemical and computational techniques.

## Abstract 2

Reversible single-electron redox transformations between diamagnetic Al^3+^ and paramagnetic “Al^2+^-like” species in organoaluminum 2,6-bisiminopyridine (BIP) chemistry.

## Introduction

Replacing costly or scarce chemical elements with more abundant alternatives is essential for advancing sustainable technologies.^1^ This task is particularly challenging for catalysts based on noble metals since these facilitate various bond formation/cleavage reactions through facile and reversible two-electron redox processes—a capability not easily replicated by more abundant metals.^2,3^ While many artificial catalysts rely on these scarce precious metals, living organisms employ enzymes containing “base” metals like Fe or Cu and, frequently, main-group elements like Zn or Mg that lack inherent redox capabilities.^4^ In these biological systems, the organic ligand framework is crucial for enabling electron, proton, or even complex molecular fragment transfers in a highly selective fashion.

Recent advances have demonstrated that base-metal catalysts can be significantly enhanced by “metal–ligand” cooperative effects, where ligands actively complement the metal by accepting or releasing electrons, protons, and/or functional units,^5,6^ thereby mimicking enzyme-like behavior. This includes the redox “non-innocence” phenomenon, whereby ligands mediate electron transfers independently from the metal,^7^ thus enhancing the versatility of catalysts in precious-metal-free transformations across a wide variety of substrates, including CO_2_.^8^ Particularly interesting are the 2,6-bis(imino)pyridine (BIP) ligands,^9^ whose ability to reversibly take up to four electrons can impart “nobility” to base metals such as Co or Fe.^10,11^ These BIP ligands enable such base metal complexes to catalyze reactions typically reserved for precious metals like C–C couplings,^12^ hydrogenations,^13^ hydrosilylations^14^ or hydroborations.^15^

**Scheme 1:**
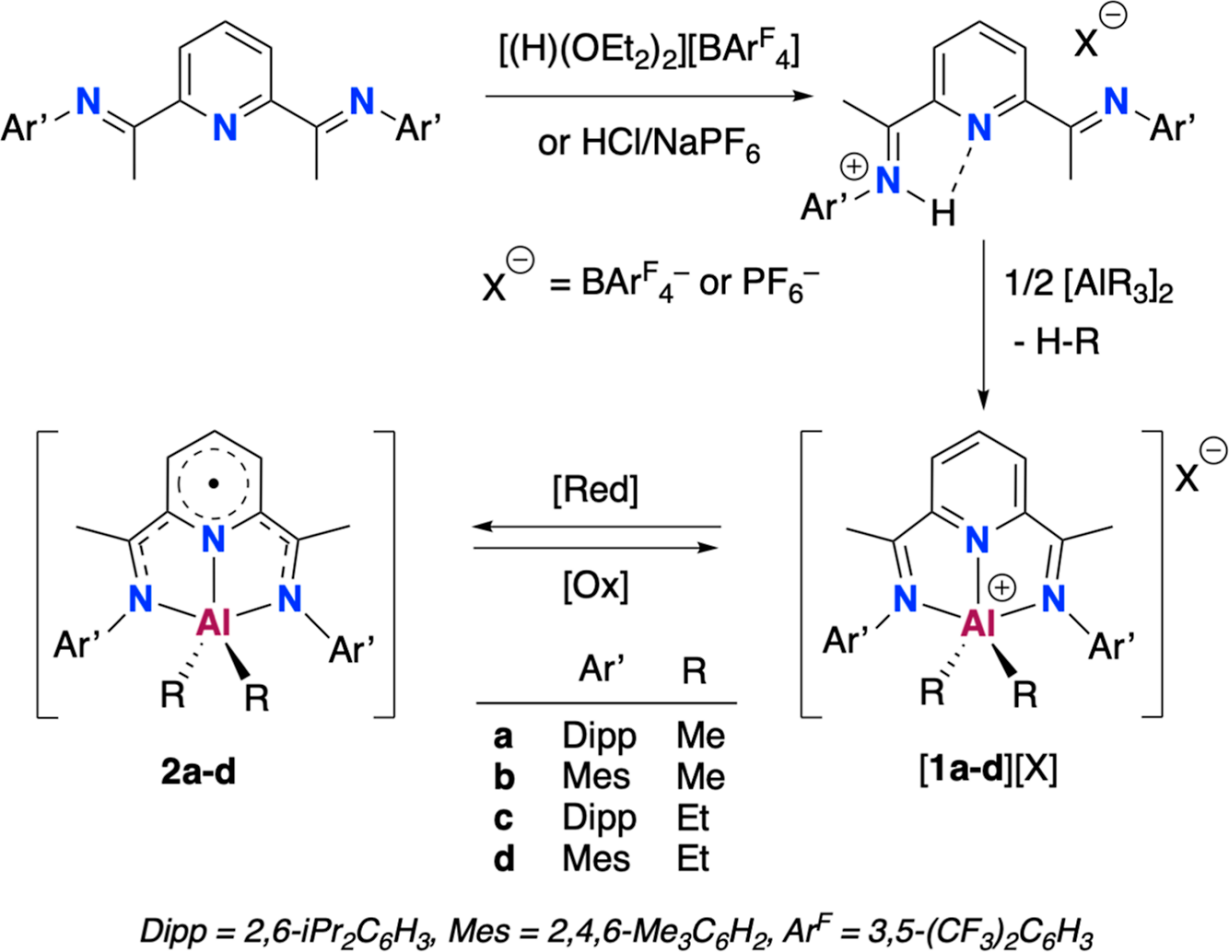
Syntheses of Cationic Aluminum Complexes [(^Ar’^BIP)AlR_2_]^+^**1**^**+**^ as BAr^F^_4_^–^ or PF_6_^–^ Salts and Reversible Redox Transformation to Paramagnetic Derivatives [(^Ar’^BIP·)AlR_2_] **2**

Building on the initial work on the interaction of [(BIP)Fe] systems with AlR_3_ reagents reported by Gambarotta,^16^ and some of us,^17^ which led to the discovery of unusual paramagnetic organoaluminum [(BIP·)AlR_2_] species, we have now devised their rational synthesis and unveiled their uniqueness to undergo reversible redox processes. Despite the established reactivity and applications in catalysis of some main-group metal [(BIP)MX_*n*_] complexes,^8,18,19^ including Berben’s systems with “AlH”,^20^ “AlCl”^21^ and “AlCl_2_”^22^ fragments, a straightforward approach to reversibly manipulate reduced “Al^2+^”-like species has remained elusive. Herein, we present a simple method for reversible ligand-centered reduction of aluminum species without significant structural alterations. We report the synthesis of cationic organoaluminum complexes [(BIP)AlR_2_]^+^, **1**^**+**^, and their reversible single-electron reduction to well-defined and isolable paramagnetic [(BIP·)AlR_2_], **2** (Scheme 1). Our study expands the understanding of BIP redox capabilities with main-group metals and showcases reversible redox organoaluminum pairs [(BIP)AlR_2_]^+/0^, illustrating their potential for redox-mediated reactivity.

## Results and Discussion

We synthesized the conjugated acids for the BIP ligands, [^Ar’^BIP(H)][BAr^F^_4_] (Scheme 1), which previously proved to be ideal precursors for accessing cationic alkyl metal complexes such as [(^Ar’^BIP)MnR][BAr^F^_4_]^23^ or [(^Ar’^BIP)ZnR][BAr^F^_4_].^24^ Reacting equimolar amounts of these pro-ligand salts with alkyl reagents AlMe_3_ or AlEt_3_ led to the formation of the BAr^F^_4_^–^ salts of the organoaluminum cations [(^Ar’^BIP)AlR_2_]^+^ as orange-yellow crystalline solids in excellent yields of 90–95%. This selectivity was confirmed by NMR spectroscopy, which showed a single Al–R cleavage in each case. Additionally, we synthesized the PF_6_^–^ salts of selected examples obtaining [**1a**][PF_6_] and [**1c**][PF_6_], in 91% and 87% yields, respectively. A related analog, [(^Dipp^BIP)AlEt_2_][Al(OC_4_F_9_)_4_], similar to [**1c**][BAr^F^_4_], was recently prepared by transmetalation from a low valent “[(^Dipp^BIP)Ga]^+^” precursor;^18^ however, only a few crystals of this compound could be isolated, contrasting with the straightforward robustness of our method.

These cationic complexes were fully characterized, including by NMR spectroscopy (see Supporting Information for full details). Their ^1^H NMR spectra show distinct, sharp signals for the BIP ligand with a characteristic resonance in the range δ 2.45–2.62 for the Me–C=N group. Additionally, these spectra display a single set of resonances for the Al-bound alkyl groups at δ ≈ −0.91 for AlMe_2_ and δ ≈ −0.05 CH_2_ and 0.12 CH_3_ for AlEt_2_, indicating chemical equivalency of both Me and Et groups.

**Figure 1:**
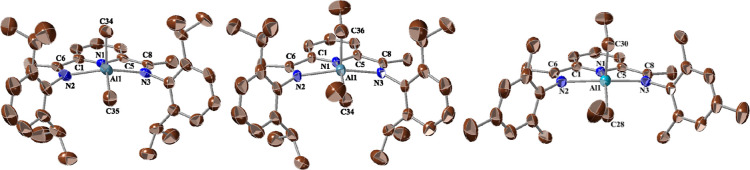
Crystal structures of [**1a**][BAr^F^_4_] (left), [**1c**][PF_6_] (center) and [**1d**][BAr^F^_4_] (right), showing only the cationic organometallic units. All hydrogen atoms have been omitted for clarity. Selected bond lengths (Å) and angles (deg), approximately symmetrical bonds are given as averages: **1a**^**+**^: Al1–N1, 2.011(4); Al–N(im), 2.152(3); C=N, 1.283(4); NC–C(Py), 1.492(4); N1–C, 1.339(4); Al1–C34, 1.959(5); Al1–C35, 1.945(5); N2–Al1–N(3), 144.32(15); N2–Al1–C35, 142.5(2). **1c^+^**: Al1–N1, 1.997(3), Al1–N(im), 2.169(2); Al–C, 1.970(3); C=N, 1.281(3); NC–C(Py), 1.483(3); N1–C, 1.342(3); N2–Al1–N3, 144.51(12); N1–Al1–C, 114.8(2)-1**d^+^:** Al1–N1, 2.019(2); Al–N(im), 2.177(1); Al–C, 1.972(2); C=N, 1.283(2); 1.484(2); N1–C, 1.344(2); N2–Al1–N3, 148.23(8); N1–Al1–C, 137.18(12).

The crystal structures of [**1a**][BAr^F^_4_], [**1c**][PF_6_], and [**1d**][BAr^F^_4_] (Figure 1) reveal pentacoordinate Al centers, robustly coordinated by the BIP ligands in a slightly distorted square pyramidal configuration. The three N donor atoms from the tripodal BIP ligand and one C-alkyl atom form the base of the pyramid, while the second alkyl group occupies the apex. The Al atom is displaced by ca. 0.6 Å above the mean coordination plane formed by the BIP ligand, approaching the geometric center of the pyramid. The intraligand C=N, (Py)C–C(N) and αC–N(Py) bond lengths (ca. 1.28, 1.48 and 1.36 Å, respectively) are similar to those observed in unperturbed BIP ligands.^9c,25^ The Al–N(Py) bond lengths are notably short (ca. 2.00 Å) compared to the relatively longer Al–N(imine) bonds (ca. 2.17 Å), presumably due to the steric hindrance of the bulky Ar groups. Although the solid-state structures of the cationic units **1**^**+**^ exhibit two distinct alkyl groups, this distinction is not observed in the solution NMR spectra, suggesting positional exchange between the apical and basal alkyl groups. This dynamic behavior evinces an intramolecular swinging motion with a low energy barrier, similar to the positional exchange processes observed in solution for analogous dialkyl complexes [(BIP)FeR_2_]^26^ and [(BIP)ZnR_2_].^27^

**Figure 2:**
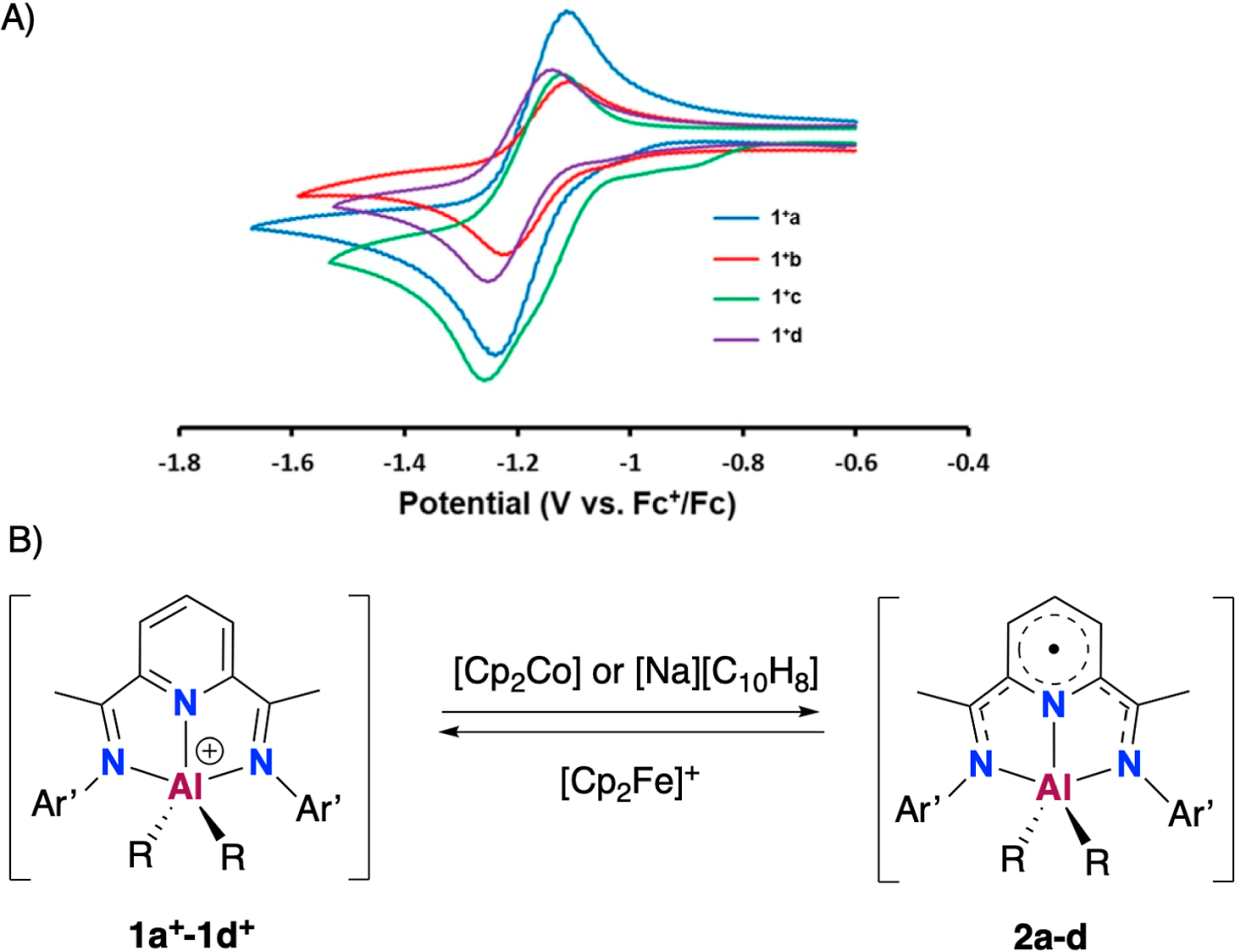
(A) CV traces for cationic complexes **1**^**+**^ (as BAr^F^_4_^–^ salts) showing a single reduction event (*E*^o^′ vs Fc^+/0^, dichloromethane, 100 mV·s^–1^, [TBA][PF_6_]). (B) Reversible redox reactions involving the paramagnetic electroneutral derivatives [(^Ar′^BIP·)AlR_2_] **2**.

The derivatives [**1**][BAr^F^_4_] demonstrate remarkable stability, despite the typical high reactivity and hydrolytic sensitivity associated with organoaluminum compounds. They do not react with weak protic reagents such as common alcohols or traces of water, likely due to the reduced Lewis acidity of the coordinatively crowded Al centers. Solutions of complexes [**1**][BAr^F^_4_] in dichloromethane remained stable for days under a normal air atmosphere as long as these were kept in closed NMR tubes. No degradation was detected by NMR spectroscopy for over 2 days. Encouraged by their chemical stability in solution, we further explored their redox properties using cyclic voltammetry (CV), as illustrated in Figure 2A. Each [(^Ar’^BIP)AlR_2_][BAr^F^_4_] complex, [**1**][BAr^F^_4_], undergoes a single reversible reduction event within diffusion-controlled processes (Figures S21, S24, S27 and S30). The redox potentials exhibit slight variations depending on the Ar’ substituents on the BIP ligands (Dipp: **1a**^**+**^ and **1c**^**+**^ with *E*^o^′ = −1.17 V; Mes **1b**^**+**^ and **1d**^**+**^ with *E*^o^′ = −1.19 V). In addition, the effect of the alkyl groups, “AlMe_2_” or “AlEt_2_”, on their redox potentials is negligible, suggesting overall that the redox process is predominantly ligand-centered, involving the conjugated pyridine and imino moieties.

Interestingly, the presence of the AlR_2_ unit appears to significantly stabilize the reduced forms, contributing to the reversibility of the redox process. In contrast, the CV study of the protonated salt of the ligand, [^Dipp^BIP(H)][BAr^F^_4_] shows an irreversible reduction at a somewhat more positive potential (*E*_P_ ≈ −1.07 V, Figure S31), highlighting the lower chemical stability of the resulting free radical [^Dipp^BIP(H)·]. Additionally, when conducted in THF, the irreversible reduction of the free ^Dipp^BIP ligand occurs at *E*_p_ ≈ −2.55 V. The significant cathodic shift of the latter CV trace compared to [^Dipp^BIP(H)]^+^ and the similar cationic species **1**^**+**^ is attributed to the absence of electrostatic compensation provided by the positive charge.

The reversibility and moderate reduction potentials of around −1.2 V for the cationic species **1a**^**+**^–**1d**^**+**^ suggested that a mild single-electron reductant such as cobaltocene, CoCp_2_, could effectively reduce cations **1**^**+**^ ([Cp_2_Co]^+/0^, *E*^o′^ = −1.33 V vs Fc^+/0^ in dichloromethane).^28^ In situ monitoring of the reaction of complexes [**1**][BAr^F^_4_] with one equivalent of Cp_2_Co by ^1^H NMR spectroscopy, in either CD_2_Cl_2_ or Tol-*d*_8_, revealed complete disappearance of the resonances of **1a**^**+**^-**1d**^**+**^ within 20 min (see Supporting Information for further details). The resulting dark red-purple solutions exhibited only signals attributed to [Cp_2_Co][BAr^F^_4_], suggesting the formation of paramagnetic aluminum species. Subsequently, adding one equivalent of ferrocenium hexafluorophosphate [Fc][PF_6_] as an oxidant to these mixtures immediately restored the ^1^H NMR signals of the diamagnetic cations **1**^**+**^. The restored spectra appeared somewhat broad and displayed slightly shifted resonances for the species **1**^**+**^, more pronounced for the **1c**^**+**^**/2c** redox pair (Figures S15–S18), likely due to minor variations in the system, such as ion pairing and exchange effects involving [BAr^F^_4_]^−^ and [PF_6_]^−^ counterions, or the presence of residual paramagnetic species. These in situ reversible redox reactions are essentially quantitative as concluded from comparing the signal intensities with the residual solvent peaks. This encouraged us to isolate the corresponding paramagnetic organoaluminum species for detailed structural analyses.

Scaling up these NMR-monitored reductions with [Cp_2_Co] enabled us to isolate and fully characterize the reduced species, namely [(^Ar’^BIP·)AlR_2_] **2a**–**2d** (Figure 2A). Specifically, **2a** and **2c** were prepared in dichloromethane where the byproduct [Cp_2_Co][BAr^F^_4_] crystallized upon cooling to −36 °C, as confirmed by X-ray diffraction (see Supporting Information for full details). The BAr^F^_4_^–^ salt of cobalticinium was removed by filtration, and precipitation with *n*-hexane allowed the isolation of **2a** and **2c**. Similarly, **2b** and **2d**, more soluble in aromatic solvents, were prepared in toluene, and the insoluble cobalticinium salt was filtered out, facilitating the isolation of **2b** and **2d**. Although complexes **2** were isolated as analytically pure red-purple solids in good to excellent yields (59–71%), this was challenging due to the high solubility of the [Cp_2_Co][BAr^F^_4_] salt. To overcome this, we replaced the cobaltocene reductant with the readily available reductant sodium naphthalene [Na][C_10_H_8_] and replaced the [BAr^F^_4_]^−^ anion with [PF_6_]^−^ to reduce the solubility issues and simplify product isolation. The reduction of [**1c**][PF_6_] with freshly titrated [Na][C_10_H_8_] in THF produced **2c** along with volatile naphthalene. The insolubility of NaPF_6_ in *n*-hexane facilitated the isolation of **2c** in almost quantitative yield (88%, Figure 2B). In contrast, although using [Cp_2_Co][BF_4_] as the reductant led to the formation of red-purple solutions, complex reaction mixtures were obtained, likely due to the incompatibility of the [BF_4_]^−^ anion with the reduced species **2**, leading to decomposition.

**Figure 3:**
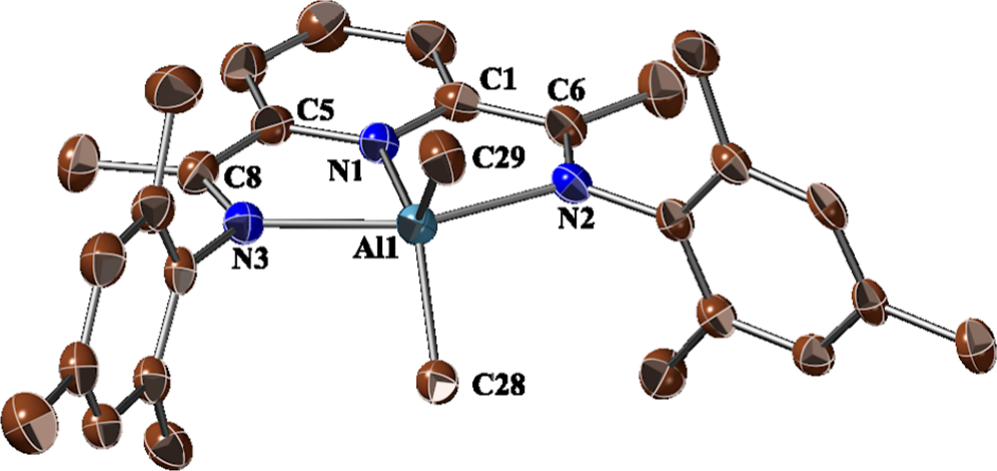
Crystal structure of **2b**. All hydrogen atoms have been omitted for clarity Thermal ellipsoids plot at 50% probability. Selected bond lengths (Å) and angles (deg): Al1–N1, 1.927(3); Al1–N2, 2.166(3); Al1–N3, 2.148(3); C6–N2, 1.304(5); C8–N3, 1.308(4); C1–C6, 1.439(6); C5–C8, 1.446(6); C1–N1, 1.381(5); C5–N1, 1.373(4); N1–Al–C29, 123.25(13); N2–Al1–N3, 153.21(10); C28–Al1–C29, 113.79(15).

We successfully grew crystals of the new reduced species **2b** by cooling a saturated solution in toluene at −36 °C, which yielded crystals suitable for X-ray diffraction analysis (Figure 3). The key structural metrics of **2b** are consistent with those previously reported for the analogs **2a**(16) and **2c**,^17^ except for the variations in the aromatic substituents on the BIP ligands. The reduction from **1**^**+**^ to **2** is characterized by significant changes in bond lengths within the BIP ligand. For instance, considering averages for the three structures reported for each type of complex,^29^ the imino C=N bond and α–C–N lengthen from 1.283(6) and 1.342(6) Å, respectively, in **1**^**+**^ to 1.313(6) and 1.373(7) Å, respectively, in **2** while the corresponding C(Py)–C(N) bonds at the pyridine ring shorten from 1.486(6) in **1**^**+**^ to 1.439(7) Å in **2**. These changes are consistent with the effects expected for a single-electron reduction of the BIP ligand.^9c,30^

Notably, the central Al–N(Py) bond in complexes **2**, averaging ca. 1.92 Å, is substantially shorter than in their oxidized counterparts **1**^**+**^ (average ca. 2.01 Å). However, the Al–N(imine) bond lengths remain similar between the oxidized and reduced complexes, averaging ca. 2.17 Å in both **1**^**+**^ and **2**. The geometry around the Al center also reflects changes due to the reduction of the systems. In particular, in the neutral methyl derivatives **2a** and **2b**, the Al atom is coplanar with the N3 donor set of the BIP ligand, aligning with a trigonal bipyramidal geometry as depicted in Figure 3. Conversely, in the slightly more hindered ethyl derivative **2c**,^17^ the Al center deviates from the N3 plane, adopting a square-pyramidal geometry, similar to that observed in its cationic counterpart **1c**^**+**^.

**Figure 4:**
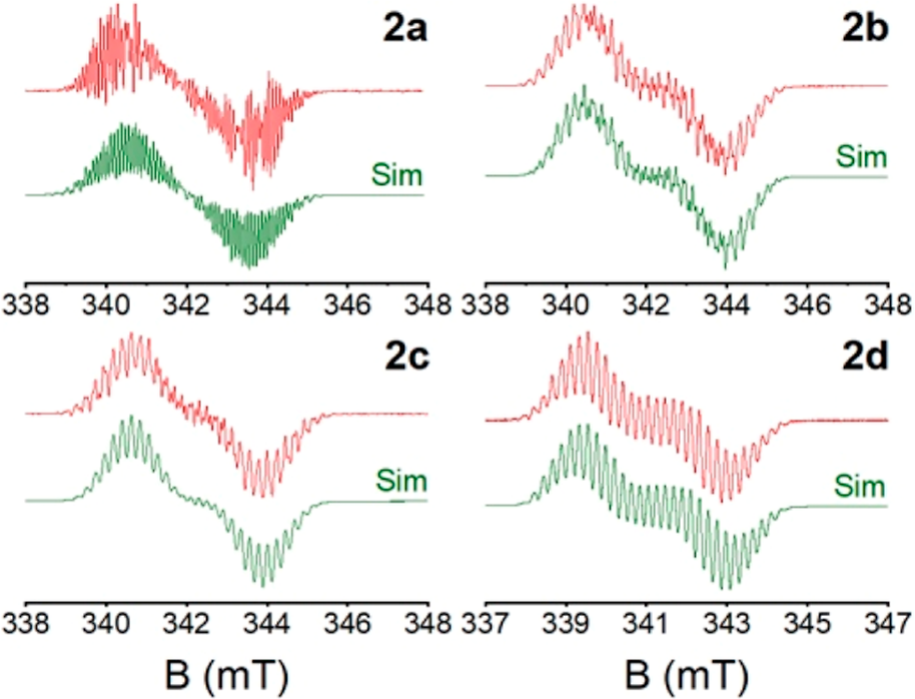
Experimental (top) and simulated (bottom) X-band EPR signals of **2a**–**d** in dichloromethane at 293 K.

The characterization of the paramagnetic species **2a**–**d** was completed with their IR and solution X-band electronic paramagnetic resonance (EPR) spectra. It is worth noting that their IR spectra lack the prominent band at ∼1600 cm^–1^, characteristic of the C=N stretch of the BIP ligand. This is consistent with the reduced π-bond character of the BIP skeleton. The EPR spectra of **2b**–**d**, along with the previously characterized **2a**(16) (Figure 4) exhibit a signal at *g* ≈ 2.00, indicative of a ligand-centered radical. In addition, the EPR spectra displayed a singular complex pattern for complexes **2** due to hyperfine couplings with the ^27^Al (*I* = 3/2) and ^15^N (*I* = 1) atoms from the pyridine and imine functions, along with several hydrogen (*I* = 1/2) nuclei. The values of these coupling constants, shown in Table 1, were determined by fitting the simulated signal shape to the experimental data. Our EPR results are in good agreement with those previously reported for **2a**,^16^ underscoring their similar electronic structures. Our data confirm that the unpaired electron is more strongly coupled with the pyridine ^15^N and the ^27^Al nuclei, which suggests a significant contribution of the Al orbitals to the molecular SOMO, thereby modulating the radical-like nature and chemical stability of the paramagnetic species **2**. The hyperfine coupling constant to the ^1^H atom at the pyridine’s position 4 is remarkably large, which indicates a significant localization of the unpaired electron at this position. The couplings to other ^1^H nuclei within the BIP ligands are smaller, and contributions from the Al-bonded methyl (Me) or ethyl (Et) groups are negligible (see Tables S1–S4). This pattern accounts for the observed high reactivity previously observed at the C4 position of the pyridine ring, although reactivity at the C3 position often competes.^31^

**Table 1:** Main Isotropic *g* and Hyperfine Constants (*A*) for Complexes **2**^a^

compound	**2a**	**2b**	**2c**	**2d**
ligand/R	^Dipp^BIP/Me	^Mes^BIP/Me	^Dipp^BIP/Et	^Mes^BIP/Et
g	2.0063	2.0049	2.0042	2.0063
A^Al^	14.83	16.43	15.30	15.25
A^Npy^	14.87	12.16	12.32	14.42
A^Nim^ (2)	4.99	2.99	5.18	4.94
A^H4^	16.04	14.47	13.28	17.50
A^H3,3′^(2)	2.57	5.13	2.16	5.02
A^CH3^ (6)	4.99	6.82	6.55	7.45
Fit RMSD	0.065	0.043	0.039	0.097

a 10^–3^ M (toluene, 298 K). Number of equivalent nuclei shown in parentheses. For full listings of *A* values, see Supporting Information (Tables S1–S4).

DFT calculations using the M06-L functional were performed to deepen our understanding of the redox behavior of the [(^Ar’^BIP)AlR_2_]^+/0^**1**^**+**^/**2** pairs in conjunction with the [Cp_2_Fe]^+/0^ and [Cp_2_Co]^+/0^ redox reagents.^32^ The crystal geometries for complexes **1**^**+**^ and **2** were optimized at the M06-L/SV(P) level, using the CPCM solvation model in dichloromethane. A set of critical bond distances, including the C=N and C–C bonds in the BIP ligand, and the Al–N bonds, as well as the geometric τ_5_ index,^33^ calculated from the N(imine)–Al–N′(imine) and the largest N(Py)–Al–C angles, were used to assess the coordination geometries (see Supporting Information, Table S7). These calculations replicated the experimental crystal structures of the couples **1**^**+**^/**2**, within an average deviation <1% between experimental and calculated bond lengths. The τ_5_ value for the computed geometries showed consistent results with experimental data, indicating near-zero values for square-pyramidal (SQP) structures of **1**^**+**^ and **2b** species and approximately 0.5 for **2a** and **2b**, indicating trigonal-bipyramidal (TBP) geometries for the latter. The τ_5_ index for an ideal trigonal-bipyramid is 1, but the nonlinearity along the main N2–Al–N3 axis decreases the geometry index to an intermediate value. For **1c**^**+**^ and **2d**, for which no experimental X-ray data is available, our calculations predicted SQP geometries (τ_5_ ≈ 0). The vibrational M06-L/SV(P) calculations also predict a significant intensity loss of the ν(C=N) stretch band, and a shift to lower frequencies (ca. 100 cm^–1^) on going from **1a**–d^**+**^ to **2a**–d, explaining the difficulty of assigning this band in the IR spectra of the reduced species.

Single-point energy corrections at the M06-L/SVPD level provided accurate enough values of the free energy changes for the reversible redox reactions **1a**–d^**+**^ ↔ **2a**–d, thereby allowing precise computation of their Δ*E*^o^ potentials vs the ferrocene (Fc) or cobaltocene (*Cc*) redox couples. As a benchmark, the calculated potentials for the [Fc]^+/0^ vs [*Cc*]^+/0^ couples dichloromethane was −1.36 V, closely aligned with the experimental −1.33 V.^28^ The calculated reduction potentials for the **1**^**+**^/**2** couples vs. were within the experimental range of −1.15 to −1.20 V vs Fc^+/0^ (Table 2), and confirming their ability to oxidize *Cc*.

**Table 2:** Experimental and Calculated Reduction Potentials (*V*) vs (Fc^+^/Fc)

redox pair	Δ*E*^o′^ Exptal^a^	Δ*E*^o^ Calcd^b^
*Cc*^+^/*Cc*^ref^	–1.33	–1.36
**1a**^**+**^**/2a**	–1.17	–1.20
**1b**^**+**^**/2b**	–1.19	–1.12
**1c**^**+**^**/2c**	–1.17	–1.11
**1d**^**+**^**/2d**	–1.19	–1.16

a vs Fc^+^/Fc, in CH_2_Cl_2_, 293 K. [TBA][PF_6_] background electrolyte.

b M06-L,CPCM-CH_2_Cl_2_/SVPD//SV(P).

Additionally, we computed the EPR parameters for paramagnetic complexes **2**. While EPR parameters for **2a** were initially approximated using the BP86 or B3LYP functionals,^16^ the *meta*-GGA functional BW6D95 paired with the EPR-II and def2-TZVP (for Al) basis sets yielded slightly more consistent results across complexes **2a**–**d**. A full description of the computational methodology and comparative results are collected in the Supporting Information (Tables S10–S13).

## Conclusions

In this study, we have established a general methodology for synthesizing reduced aluminum paramagnetic species [(BIP·)AlR_2_] through the chemical reduction of their isostructural cations [(BIP)AlR_2_]^+^ using mild one-electron redox reagents, like [Cp_2_Co]. The organoaluminum precursors [(BIP)AlR_2_]^+^ were conveniently synthesized as stable BAr^F^_4_^–^ or PF_6_^–^ salts by the reaction of the conjugate acids of BIP ligands, with commercial aluminum reagents, AlMe_3_ and AlEt_3_. Furthermore, we demonstrated the reversibility of the redox process involving the [(BIP)AlR_2_]^+/0^ couples, both electrochemically via voltammetry and chemically with [*Cc*] and [Fc]^+^ reagents. The redox processes linking the diamagnetic [(BIP)AlR_2_]^+^ and paramagnetic [(BIP·)AlR_2_] species were characterized in detail using a suite of spectroscopic, crystallographic, electrochemical, and computational methods to confirm their mild exchange potentials and redox reversibility. These findings significantly advance our understanding of BIP ligand redox capabilities with main-group metals, particularly with Earth-abundant aluminum, paving the way for future studies into their reactivity and potential applications in redox catalysis.

## Experimental Section

Most compounds presented in this work are highly sensitive to oxygen and moisture. Therefore, inert atmosphere Schlenk techniques and a N_2_-filled glovebox were routinely used in manipulations and procedures. All solvents (dichloromethane, toluene, *N*-hexane, tetrahydrofuran and diethyl ether) were rigorously degassed, dried, and distilled immediately prior to use. Instrumentation and procedures, NMR, IR and EPR spectra of new compounds, elemental analysis (EA), X-ray diffraction studies, electrochemistry and computational details are included in the Supporting Information.

Solutions of HCl (2 M in diethyl ether) and NaPF_6_ were supplied by Thermo Scientific, stored in a glovebox and employed as received. Aluminum trialkyls (AlMe_3_, pure and 2 M in toluene and AlEt_3_ 1.0 M in hexane) were purchased from Sigma-Aldrich and used as received. The protonated tetraarylborate salts [H^Dipp^BIP][BAr^F^_4_] and [H^Mes^BIP][BAr^F^_4_] where ^Dipp^BIP is 2,6-[2,6-^i^Pr_2_C_6_H_3_N=C(Me)]_2_-C_5_H_3_N, ^Mes^BIP is 2,6-[2,4,6-Me_3_C_6_H_2_N=C(Me)]_2_-C_5_H_3_N, and Ar^F^_4_ is (3,5-(CF_3_)_2_C_6_H_3_) respectively, were prepared according to our own reported methodology.^23^ Bis(cyclopentadienyl)cobalt (Cp_2_Co, where Cp=C_5_H_5_) in powder form, purchased from Alfa Aesar was stored at −30 °C within a glovebox and used without further purification. Ferrocenium hexafluorophosphate ([Cp_2_Fe][PF_6_] with Cp=C_5_H_5_, abbreviated as [Fc][PF_6_]), sourced from Merck, was also stored in a glovebox and used as received.

### Synthesis of [^Dipp^BIPH][Cl]

A 2 M solution in Et_2_O of HCl (206.0 μL, 0.412 mmol) was added dropwise to stirred suspension of ^Dipp^BIP (198.1 mg, 0.412 mmol) in Et_2_O (20 mL) at −30 °C. The resulting pale-yellow solution was stirred for 30 min at room temperature. Then, the solvent was evaporated under vacuum, isolating a pale-yellow solid residue that corresponded to [^Dipp^BIPH][Cl] (204.4 mg; 96%). ^**1**^**H NMR** (C_6_D_6_, 25 °C, 400 MHz): δ 1.13 (d, ^3^*J*_HH_ = 6.9 Hz, 12H, CH*Me*Me), 1.22 (d, ^3^*J*_HH_ = 6.9 Hz, 12H, CHMe*Me*), 2.27 (s, 6H, *Me*(CN)), 2.91 (sept, ^3^*J*_HH_ = 7.0 Hz, 4H, *CH*MeMe), 7.15 (m, 6H, CH_N–Ar_), 7.43 (br s, 1H, 4-CH_py_), 8.66 (d, ^3^*J*_HH_ = 7.9 Hz, 2H, 3-CH_py_). ^**13**^**C{**^**1**^**H} NMR** (C_6_D_6_, 25 °C, 100 MHz): δ 17.57 (*Me*–CN), 23.02 (CHMe*Me*), 23.67 (CH*Me*Me), 28.96 (*C*HMeMe), 123.78 (3,5-*C*H_Py_), 125.43 (*m*-CH_N–Ar_), 136.91 (*o-C*_N–Ar_), 137.76 (4-*C*H_Py_), 144.46 (*i-C*H_N–Ar_), 154.05 (2-*C*H_Py_), 168.97 (Me–*C*N). *The signal attributed to p-CH*_*N–Ar*_ was not-observed. **EA for C_33_H_44_ClN_3_** (found vs calculated, crystalline sample): C 76.42 (76.49), H 8.28 (8.56), N 8.29 (8.11).

### Synthesis of [^Dipp^BIPH][PF_6_]

In a nitrogen-filled glovebox, 53.7 mg (0.320 mmol) of NaPF_6_ are slowly added to a pale-yellow solution of [^Dipp^BIPH][Cl] (150 mg, 0.290 mmol), dissolved in 20 mL of THF, cooled to −30 °C. The solution instantly changed to orange. After 1 h stirring at room temperature, the solvent and volatiles were removed at reduced pressure. The solid residue was extracted in dichloromethane, filtered and dried again. The NMR spectra of the isolated orange solid corresponded exclusively to compound [^Dipp^BIPH][PF_6_] (163.6 mg, 90%). ^**1**^**H NMR** (CD_2_Cl_2_, 25 °C, 400 MHz): δ 1.14 (d, ^3^*J*_HH_ = 6.9 Hz, 12H, CH*Me*Me), 1.19 (d, ^3^*J*_HH_ = 7.0 Hz, 12H, CHMe*Me*), 2.45 [s, 6H, *Me*(CN)], 2.70 (sept, ^3^*J*_HH_ = 7.1 Hz 4H, *CH*MeMe), 7.25 (br s, 6H, CH_N–Ar_) 8.46 (br s, 1H, 4-CH_py_), 8.65 (br s, 2H, 3-CH_py_). ^**19**^**F{**^**1**^**H} NMR** (CD_2_Cl_2_, 25 °C, 376 MHz): δ −72.91 (d, *J*_FP_ = 718.0 Hz, [P*F*_6_]^−^). ^**31**^**P{**^**1**^**H} NMR** (CD_2_Cl_2_, 25 °C, 162 MHz): δ −144.56 (sept, *J*_PF_ = 716.0 Hz, [*P*F_6_]^−^). ^**13**^**C{**^**1**^**H} NMR** (CD_2_Cl_2_, 25 °C, 100 MHz): δ 17.39 (*Me*–CN), 22.87 (CHMe*Me*), 23.58 (CH*Me*Me), 29.07 (*C*HMeMe), 124.12 (3,5-*C*H_Py_), 127.54 (*m*-CH_N–Ar_), 128.91 (*p-C*H_N–Ar_), 138.07 (*o-C*_N–Ar_), 143.79 (4-*C*H_Py_), *not observed* (*i-C*H_N–Ar_), *not observed* (2-*C*H_Py_), 168.50 (Me–*C*N). **EA for C**_**33**_**H**_**44**_**PF**_**6**_**N**_**3**_ (found vs calculated, crystalline sample): C 63.12 (63.15), H 7.31 (7.07), N 6.83 (6.69).

### Synthesis of [Al(Me)_2_(^Dipp^BIP)][PF_6_] **([1a][PF**_**6**_**])**

A 5 mL CH_2_Cl_2_ cold (−25 °C) colorless solution of AlMe_3_ (32 μL, 0.313 mmol) was slowly added to another orange CH_2_Cl_2_ solution (15 mL) of [^Dipp^BIPH][PF_6_] (178.5 mg; 0.285 mmol) at the same temperature, immediately changing to a yellow-brown. The mixture was stirred vigorously for 10 min, and then, the solvent and volatiles were removed under vacuum. The solid residue was washed with hexane (3 × 5 mL). Finally, the product was filtered and dried at reduced pressure, obtaining a yellow-brown powdery solid (177.2 mg, 91%) that corresponded to complex **[1a][PF**_**6**_**]**. ^**1**^**H NMR** (CD_2_Cl_2_, 25 °C, 300 MHz): δ −0.92 (s, 6H, Al*Me*_2_), 1.09 (d, ^3^*J*_HH_ = 7.0 Hz, 12H, CH*Me*Me), 1.23 (d, ^3^*J*_HH_ = 6.9 Hz, 12H, CHMe*Me*), 2.57(sept, ^3^*J*_HH_ = 6.9 Hz, 4H, *CH*MeMe), 2.59 (s, 6H, *Me*(CN)), 7.30 (d, ^3^*J*_HH_ = 7.3 Hz, 4H, *m*-CH_N–Ar_), 7.38 (t, ^3^*J*_HH_ = 7.4 Hz, 2H, *p*-CH_N–Ar_), 8.68 (d, ^3^*J*_HH_ = 7.9 Hz, 2H, 3-CH_py_), 8.95 (t, ^3^*J*_HH_ = 8.1 Hz, 1H, 4-CH_py_). ^**19**^**F{**^**1**^**H} NMR** (CD_2_Cl_2_, 25 °C, 376 MHz): δ −72.80 (d, *J*_FP_ = 718.0 Hz, [P*F*_6_]^−^). ^**31**^**P{**^**1**^**H} NMR** (CD_2_Cl_2_, 25 °C, 162 MHz): δ −144.55 (sept, *J*_PF_ = 716.0 Hz, [*P*F_6_]^−^). ^**13**^**C{**^**1**^**H} NMR** (CD_2_Cl_2_, 25 °C, 100 MHz): δ −7.93 (Al*Me*_2_), 19.27 (*Me*–CN), 24.15 (CHMe*Me*), 25.15 (CH*Me*Me), 29.16 (*C*HMeMe), 125.14 (*m*-CH_N–Ar_), 128.70 (3,5-*C*H_Py_), 129.35 (*p-C*H_N–Ar_), 139.35 (*o-C*_N–Ar_), 140.25 (*i-C*_N–Ar_), 147.73 (4-*C*H_Py_), 148.07 (2-*C*H_Py_), 171.10 (Me–*C*N). IR (KBr/Nujol, cm-1): ν 1600 (Intense, C=N stretch). **EA for C**_**35**_**H**_**49**_**AlPF**_**6**_**N**_**3**_ (found vs calculated, crystalline sample): C 61.46 (61.48), H 7.48 (7.22), N 6.21 (6.15).

### Synthesis of [Al(Me)_2_(^Dipp^BIP)][BAr^F^_4_] **([1a][BAr**^**F**^_**4**_**])**

A dichloromethane (5 mL) solution of trimethyl aluminum (AlMe_3_, 11.6 μL, 0.121 mmol) was added via pipette to another CH_2_Cl_2_ solution (15 mL) of [(^Dipp^BIPH)][BAr^F^_4_] (142.5 mg; 0.101 mmol) both at −25 °C, observing an instantaneous change from orange to yellow-green. The reaction mixture was magnetically stirred for 16 h. Then, the solvent and volatiles were removed at vacuo, isolating a solid which upon washing with hexane (3 × 5 mL), filtration and drying allow obtaining a yellow-green powdery solid (131.6 mg, 93%) that corresponded with (**[1a][BAr**^**F**^_**4**_**]**). Recrystallization in a cold (−35 °C) CH_2_Cl_2_: hexane (5:1) solution produced yellow cubic crystals suitable for X-ray diffraction studies. ^**1**^**H NMR** (CD_2_Cl_2_, 25 °C, 400 MHz): δ −0.91 (s, 6H, Al*Me*_2_), 1.08 (d, ^3^*J*_HH_ = 6.9 Hz, 12H, CH*Me*Me), 1.22 (d, ^3^*J*_HH_ = 6.9 Hz, 12H, CHMe*Me*), 2.51 (sept, ^3^*J*_HH_ = 6.9 Hz, 4H, *CH*MeMe), 2.55 (s, 6H, *Me*(CN)), 7.31 (d, ^3^*J*_HH_ = 7.3 Hz, 4H, *m*-CH_N–Ar_), 7.40 (t, ^3^*J*_HH_ = 7.4 Hz, 2H, *p*-CH_N–Ar_), 7.55 (s, 4H, *p*-CH_Ar_ BAr^F^_4_), 7.72 (br s, 8H, *o*-CH_Ar_ BAr^F^_4_), 8.45 (d, ^3^*J*_HH_ = 7.9 Hz, 2H, 3-CH_py_), 8.68 (d, ^3^*J*_HH_ = 8.0 Hz, 1H, 4-CH_py_). ^**19**^**F{**^**1**^**H} NMR** (CD_2_Cl_2_, 25 °C, 376 MHz): δ −62.87 (s, BAr^F^_4_). ^**11**^**B{**^**1**^**H} NMR** (CD_2_Cl_2_, 25 °C, 128 MHz): δ −6.61 (s, BAr^F^_4_). ^**13**^**C{**^**1**^**H} NMR** (CD_2_Cl_2_, 25 °C, 100 MHz): δ −7.80 (Al*Me*_2_), 19.22 (*Me*–CN), 24.09 (CHMe*Me*), 25.01 (CH*Me*Me), 29.33 (*C*HMeMe), 117.90 (*p-C*H_Ar_ BAr^F^_4_), 123.64 (*m*-CH_N–Ar_), 125.33 (*C*F_3_ BAr^F^_4_), 126.35 (3,5-*C*H_Py_), 128.35 (*p-C*H_N–Ar_), 129.23 (q, ^2^*J*_CF_ = 33 Hz, *C*-CF_3_ BAr^F^_4_), 135.21 (*o-C*H_Ar_ BAr^F^_4_), 138.95 (*o-C*_N–Ar_), 139.81 (*i-C*_N–Ar_), 147.16 (4-*C*H_Py_), 147.94 (2-*C*H_Py_), 162.11 (q, ^1^*J*_CB_ = 50 Hz, *i-C*_Ar_ BAr^F^_4_), 170.12 (Me–*C*N). **EA for C**_**67**_**H**_**61**_**AlBF**_**24**_**N**_**3**_ (found vs calculated, crystalline sample): C 57.42 (57.40), H 4.51 (4.39), N 2.85 (3.00).

### Synthesis of (^Dipp^BIP·)AlMe_2_ (**2a**)

A J. Young’s ampule was loaded with 200 mg (0.142 mmol) of **[1a][BAr**^**F**^_**4**_**]** and 27 mg (0.142 mmol) of Cp_2_Co. The solids were combined with 3 mL of dry dichloromethane to form a dark purple suspension. This mixture was stirred vigorously at room temperature and progress was monitored via ^1^H NMR analysis of aliquots. After 4 h, the resonances attributed to **[1a][BAr**^**F**^_**4**_**]** disappeared. The mixture was then filtered through a cannula into a Schlenk tube and cooled to −36 °C. After 16 h, single crystals of [Cp_2_Co][BAr^F^_4_] suitable for X-ray crystallography were harvested. This crystalline material was filtered to give a dark purple solution. The resulting dark purple solution was concentrated to 1 mL, and the addition of 20 mL of anhydrous *n*-hexane precipitated a purple solid. This product was isolated by cannula filtration and dried under high vacuum (<10^–2^ mbar), affording **(**^**Dipp**^**BIP·)AlMe**_**2**_**(2a)** as a dark purple solid (Yield: 46 mg, 0.084 mmol, 59%.), according to its EA and its EPR spectrum, which can be checked in the Supporting Information. Single crystals suitable for X-ray diffraction analysis were obtained by cooling a toluene solution of **2a** at −36 °C for 24 h. The X-ray crystal structure of **(**^**Dipp**^**BIP·)AlMe**_**2**_**(2a)** obtained in this study is consistent with the structure earlier reported by Gambarotta.^2^ This confirmation reinforces the reproducibility of the crystalline structure under the described synthesis conditions.

**Note on**^**1**^**H NMR Data:***The absence of*^*1*^*H NMR resonances for* (^***Dipp***^***BIP·***)***AlMe***_***2***_ (**2a**) *is attributable to the paramagnetic nature of the compound.***EA for C**_**35**_**H**_**49**_**AlN**_**3**_ (found vs calculated, crystalline sample): C 78.08 (78.03), H 9.30 (9.17), N 7.99 (7.80). **IR (KBr/Nujol, cm**^**–1**^**)**: ν 1644, 1607, 1587 (medium intensity, C=C Py and C=N imine). *The bands observed at 1644 cm*^*–1*^*for the C*=*C stretch is characteristic of the dearomatized pyridine ring.*

### Synthesis of [Al(Me)_2_(^Mes^BIP)][BAr^F^_4_] **([1b][BAr**^**F**^_**4**_**])**

To 148 mg (0.118 mmol) of [(^Mes^BIPH)][BAr^F^_4_] in 15 mL of CH_2_Cl_2_ at −25 °C was added 13.5 μL (0.142 mmol) of AlMe_3_ in 5 mL of CH_2_Cl_2_ via syringe. Then, the same experimental protocol used for the preparation of **[1a][BAr**^**F**^_**4**_**]** was applied to the synthesis of **[1b][BAr**^**F**^_**4**_**]**, isolating a brown solid (150.8 mg, 95%) which according to the NMR spectra corresponded to the expected compound **[1b][BAr**^**F**^_**4**_**]**. Recrystallization attempts did not afford suitable crystals for X-ray diffraction studies. ^**1**^**H NMR** (CD_2_Cl_2_, 25 °C, 400 MHz): δ −0.91 (s, 6H, Al*Me*_2_), 2.01 (s, 12H, *o*-*Me*_N–Ar_), 2.36 (s, 6H, *p*-*Me*_N–Ar_), 2.47 (s, 6H, *Me*–CN), 7.01 (s, 4H, *m*-CH_N–Ar_), 7.59 (s, 4H, *p*-CH_Ar_ BAr^F^_4_), 7.76, (br s, 8H, *o*-CH_Ar_ BAr^F^_4_), 8.48 (d, ^3^*J*_HH_ = 8.0 Hz, 2H, 3-CH_py_), 8.73 (d, ^3^*J*_HH_ = 7.8 Hz, 1H, 4-CH_py_). ^**19**^**F{**^**1**^**H} NMR** (CD_2_Cl_2_, 25 °C, 376 MHz): δ −62.82 (s, BAr^F^_4_). ^**11**^**B{**^**1**^**H} NMR** (CD_2_Cl_2_, 25 °C, 128 MHz): δ −6.61 (s, BAr^F^_4_). ^**13**^**C{**^**1**^**H} NMR** (CD_2_Cl_2_, 25 °C, 100 MHz): δ −9.67 (Al*Me*_2_), 14.29, 17.19 (*Me*–CN), 18.37 (*o*-*Me*_N–Ar_), 20.87 (*p*-*Me*_N–Ar_), 117.90 (*p-C*H_Ar_ BAr^F^_4_), 123.64 (*C*F_3_ BAr^F^_4_), 128.10 (3,5-*C*H_Py_), 128.53 (*p-C*_N–Ar_), 129.28 (q, ^2^*J*_CF_ = 33 Hz, *C*-CF_3_ BAr^F^_4_), 130.36 (*m*-CH_N–Ar_), 135.25 (*o-C*H_Ar_ BAr^F^_4_), 137.87 (*o-C*_N–Ar_), 138.95 (*i-C*_N–Ar_), 147.50 (4-*C*H_Py_), 149.14 (2-*C*H_Py_), 162.10 (q, ^1^*J*_CB_ = 50 Hz, *i-C*_Ar_ BAr^F^_4_), 169.22 (Me–*C*N). **EA for** C_61_H_49_AlBF_24_N_3_ (found vs calculated, bulk sample): C 55.65 (55.60), H 4.04 (3.75), N 3.22 (3.19).

### Synthesis of (^Mes^BIP·)AlMe_2_ (**2b**)

A J. Young’s ampule was charged with 233 mg (0.177 mmol) of **[1b][BAr**^**F**^_**4**_**]** and 33 mg (0.177 mmol) of Cp_2_Co. The solids were combined with 3 mL of anhydrous toluene, forming a dark red suspension. This mixture was stirred vigorously at room temperature and progress was monitored via ^1^H NMR analysis of aliquots. After 4 h, the resonances attributed to **[1b][BAr**^**F**^_**4**_**]**disappeared. After this time, the reaction mixture was filtered into a Schlenk tube via cannula to remove the salt [Cp_2_Co][BAr^F^_4_] precipitated. Then, dry *n*-hexane was added dropwise to the resulting solution to point of incipient crystallization. The Schlenk tube was then stored at −36 °C overnight. The resultant solid was isolated via cannula filtration, dried under high vacuum (<10^–2^ mbar), yielding **(**^**Mes**^**BIP·)AlMe**_**2**_ (**2b**) as a dark red solid. Yield: 57 mg, 0.125 mmol, 71%. Suitable plate-shaped single crystals of **2b** for X-ray diffraction analysis were obtained by layering a toluene solution (1 mL) of the compound with *n*-hexane (2 mL) and progressively cooling down the mixture to 0 °C for 4 h and then from 0 °C to −36 °C overnight. **Note on**^**1**^**H NMR data:***The absence of*^*1*^*H NMR resonances for* (^***Mes***^***BIP·***)***AlMe***_***2***_**(2b)***is attributable to the paramagnetic nature of the compound.***EA for C**_**29**_**H**_**37**_**AlN**_**3**_ (found vs calculated, bulk sample): C 76.63 (76.62), H 8.30 (8.20), N 9.01 (9.24). **IR (KBr/Nujol, cm**^**–1**^**):** ν 1640, 1609, 1557 (medium to weak intensity, C=C Py and C=N imine). *The band observed at 1640 cm*^*–1*^*for the C*=*C stretch is characteristic of the dearomatized pyridine ring.* The **EPR** of **2b** spectrum is available in the Supporting Information.

### Synthesis of [Al(Et)_2_(^Dipp^BIP)][BAr^F^_4_] **[1c][BAr**^**F**^_**4**_**]**

The synthesis of **[1c][BAr**^**F**^_**4**_**]** was carried out following a similar experimental protocol than that applied for the preparation of **[1a][BAr**^**F**^_**4**_**]**. To 120.0 mg (0.089 mmol) of [(^Dipp^BIP)]^+^[BAr^F^_4_]^−^ in 15 mL of CH_2_Cl_2_ at −25 °C was added 106.8 μL (0.107 mmol) of AlEt_3_ 106.8 μL (0.107 mmol, 1 M in *n*-hexane). An orange powdery solid (114.5 mg, 90%) was isolated which according to the NMR spectra corresponded solely to compound **[1c][BAr**^**F**^_**4**_**]**. ^**1**^**H NMR** (CD_2_Cl_2_, 25 °C, 300 MHz): δ −0.05 (q, ^3^*J*_HH_ = 8.0 Hz, 4H, Al(*CH*_*2*_CH_3_)_2_), 0.11 (t, ^3^*J*_HH_ = 7.0 Hz 6H, Al(CH_2_*CH*_*3*_)_2_), 1.09 (d, ^3^*J*_HH_ = 6.9 Hz, 12H, CH*Me*Me), 1.26 (d, ^3^*J*_HH_ = 6.8 Hz, 12H, CHMe*Me*), 2.49 (sept, ^3^*J*_HH_ = 6.5 Hz, 4H, *CH*MeMe), 2.57 (s, 6H, *Me*(CN)), 7.32 (d, ^3^*J*_HH_ = 7.7 Hz, 4H, *m*-CH_N–Ar_), 7.42 (t, ^3^*J*_HH_ = 7.7 Hz, 2H, *p*-CH_N–Ar_), 7.56 (s, 4H, *p*-CH_Ar_ BAr^F^_4_), 7.73 (br s, 8H, *o*-CH_Ar_ BAr^F^_4_), 8.47 (d, ^3^*J*_HH_ = 8.0 Hz, 2H, 3-CH_py_), 8.65 (d, ^3^*J*_HH_ = 8.0 Hz, 1H, 4-CH_py_). ^**19**^**F{**^**1**^**H} NMR** (CD_2_Cl_2_, 25 °C, 376 MHz): δ −62.82 (s, BAr^F^_4_). ^**11**^**B{**^**1**^**H} NMR** (CD_2_Cl_2_, 25 °C, 128 MHz): δ −6.59 (s, BAr^F^_4_). ^**13**^**C{**^**1**^**H} NMR** (CD_2_Cl_2_, 25 °C, 100 MHz): δ −1.29 (Al(CH_2_*C*H_3_)_2_), 8.10 (Al(*C*H_2_CH_3_)_2_), 19.20 (*Me*–CN), 23.92 (CHMe*Me*), 25.11 (CH*Me*Me), 29.31 (*C*HMeMe), 117.91 (*p-C*H_Ar_ BAr^F^_4_), 123.66 (*m*-CH_N–Ar_), 125.34 (*C*F_3_ BAr^F^_4_), 126.36 (3,5-*C*H_Py_), 127.99 (*p-C*H_N–Ar_), 129.30 (q, ^2^*J*_CF_ = 30 Hz, *C*-CF_3_ BAr^F^_4_), 135.23 (*o-C*H_Ar_ BAr^F^_4_), 139.55 (*o-C*_N–Ar_), 139.85 (*i-C*_N–Ar_), 146.77 (4-*C*H_Py_), 147.72 (2-*C*H_Py_), 162.21 (q, ^1^*J*_CB_ = 50 Hz, *i-C*_Ar_ BAr^F^_4_), 170.41 (Me–*C*N). **EA for C**_**69**_**H**_**65**_**AlBF**_**24**_**N**_**3**_ (found vs calculated, bulk sample): C 57.88 (57.95), H 4.21(4.58), N 3.16(2.94).

### Synthesis of [Al(Et)_2_(^Dipp^BIP)][PF_6_] **([1c][PF**_**6**_**])**

An orange solution of [^Dipp^BIPH][PF_6_] (130.2 mg; 0.208 mmol) in 15 mL of CH_2_Cl_2_ was reacted with another of AlEt_3_ 1 M in hexane (208 μL, 0.208 mmol) at −30 °C. The resultant solution immediately changed to a yellow-brown color and this was kept under magnetic stirring for 10 min. The solvent and volatiles were then removed under vacuum, isolating a crude orange solid. This residue was washed with hexane (3 × 5 mL), filtered and evaporated to dryness, producing a yellow-brown powdery solid corresponding with **[1c][PF**_**6**_**]** (128.3 mg, 87%). Then, this was dissolved in 1 mL of CH_2_Cl_2_ and subsequently 0.1 mL of hexane. The resulting solution was stored at −30 °C and after 72 h, a yellow cubic microcrystalline solid appeared that was suitable for X-ray diffraction studies. ^**1**^**H NMR** (CD_2_Cl_2_, 25 °C, 300 MHz): δ −0.06 (q, ^3^*J*_HH_ = 8.3 Hz, 4H, Al(*CH*_*2*_CH_3_)_2_), 0.13 (t, ^3^*J*_HH_ = 7.9 Hz 6H, Al(CH_2_*CH*_*3*_)_2_), 1.12 (d, ^3^*J*_HH_ = 6.9 Hz, 12H, CH*Me*Me), 1.28 (d, ^3^*J*_HH_ = 6.9 Hz, 12H, CHMe*Me*), 2.55 (sept, ^3^*J*_HH_ = 6.5 Hz, 4H, *CH*MeMe), 2.62 (s, 6H, *Me*(CN)), 7.32 (d, ^3^*J*_HH_ = 7.7 Hz, 4H, *m*-CH_N–Ar_), 7.41 (t, ^3^*J*_HH_ = 7.7 Hz, 2H, *p*-CH_N–Ar_), 8.69 (d, ^3^*J*_HH_ = 8.0 Hz, 2H, 3-CH_py_), 8.92 (t, ^3^*J*_HH_ = 8.0 Hz, 1H, 4-CH_py_). ^**19**^**F{**^**1**^**H} NMR** (CD_2_Cl_2_, 25 °C, 376 MHz): δ −72.96 (d, *J*_FP_ = 710.0 Hz, [P*F*_6_]^−^). ^**31**^**P{**^**1**^**H} NMR** (CD_2_Cl_2_, 25 °C, 162 MHz): δ −144.54 (sept, *J*_PF_ = 712.0 Hz, [*P*F_6_]^−^). ^**13**^**C{**^**1**^**H} NMR** (CD_2_Cl_2_, 25 °C, 100 MHz): δ −1.40 (Al(CH_2_*C*H_3_)_2_), 8.14 (Al(*C*H_2_CH_3_)_2_), 19.19 (*Me*–CN), 23.91 (CHMe*Me*), 25.20 (CH*Me*Me), 29.12 (*C*HMeMe), 125.10 (*m*-CH_N–Ar_), 128.73 (3,5-*C*H_Py_), 128.89 (*p-C*H_N–Ar_),, 139.87 (*o-C*_N–Ar_), 140.18 (*i-C*_N–Ar_), 147.47 (4-*C*H_Py_), 147.67 (2-*C*H_Py_), 171.33 (Me–*C*N). **EA for C**_**37**_**H**_**53**_**AlPF**_**6**_**N**_**3**_ (found vs calculated, bulk sample): C 62.44 (62.43), H 7.50 (7.51), N 5.92 (5.90). **IR (KBr/Nujol, cm**^**–1**^**):** ν 1597 (C=N, BIP).

### Synthesis of (^Dipp^BIP·)AlEt_2_ (**2c**). *Route A*

A J. Young’s ampule was loaded with 397 mg (0.277 mmol) of **[1c][BAr**^**F**^_**4**_**]** and 54 mg (0.282 mmol) of Cp_2_Co. The solids were combined with 3 mL of anhydrous dichloromethane, giving a dark red suspension. This mixture was stirred vigorously at room temperature and progress was monitored via ^1^H NMR analysis of aliquots. After 4 h, the resonances attributed to **[1c][BAr**^**F**^_**4**_**]** disappeared. Subsequent cooling of the mixture to −36 °C initiated the precipitation of [Cp_2_Co][BAr^F^_4_]. After 2 h at this temperature, the cold mixture was filtered and the filtrate was dried under vacuum (<10^–2^ mbar) to yield **(**^**Dipp**^**BIP·)AlEt**_**2**_**(2c)** as a dark golden/brown solid. Yield: 81 mg, 0.142 mmol, 51%. Crystallization was attempted with several solvent mixtures but not achieved. **Note on**^**1**^**H NMR data**:*The absence of*^*1*^*H NMR resonances for* (^***Dipp***^***BIP·***)***AlEt***_***2***_**(2c)***is attributable to the paramagnetic nature of the compound.***IR (KBr/Nujol, cm**^**–1**^**):** ν 1643, 1607, 1584 (medium to weak intensity, C=C Py and C=N imine). *The band observed at 1643 cm*^*–1*^*for the C*=*C stretch is characteristic of the dearomatized pyridine ring.*

### Synthesis of **2c**. *Route B*

Compound **[1c][PF**_**6**_**]** (82.2 mg; 0.115 mmol) was dissolved in 10 mL of THF and cooled to −30 °C. Then, 131 μL of a solution of sodium-naphthalene (0.88 M in THF, 0.115 mmol) was added at the same temperature. The orange solution turned brown immediately, being stirred for 30 min at room temperature. Then, the solvent and volatiles were dried under vacuum. The solid residue was then extracted in hexane (3 × 5 mL), filtered and brought to dryness, yielding 57.6 mg (88%) of a brown solid corresponding to complex **2c**. Although the synthesis and full characterization of **2c** has already been reported by us,^17^ this route provide pure samples as the corresponding **EA for C**_**37**_**H**_**53**_**AlN**_**3**_ (found vs calculated): C 78.48 (78.40), H 9.66 (9.48), N 7.30 (7.41) and **EPR spectrum** (see Supporting Information) confirmed.

### Synthesis of [Al(Et)_2_(^Mes^BIP)][BAr^F^_4_] **([1d][BAr**^**F**^_**4**_**])**

The same experimental protocol employed for the preparation of **([1a][BAr**^**F**^_**4**_**])** was used for the synthesis of **([1d][BAr**^**F**^_**4**_**])**. To 252.0 mg (0.200 mmol) of [(^Mes^BIPH)][BAr^F^_4_], 239.8 μL (0.240 mmol) of AlEt_3_ (1 M in hexane) was added via pipet, both at approximately −25 °C. An orange solid that according to the NMR spectra corresponded to **[1d][BAr**^**F**^_**4**_**]**. was isolated (250.2 mg, 93%). Then, this was dissolved in a CH_2_Cl_2_/hexane (4:1) mixture and stored at −35 °C. An orange crystalline solid suitable for X-ray diffraction studies was obtained within 48 h. ^**1**^**H NMR** (CD_2_Cl_2_, 25 °C, 300 MHz): δ −0.05 (q, ^3^*J*_HH_ = 8.4 Hz, 4H, Al(*CH*_*2*_CH_3_)_2_), 0.25 (t, ^3^*J*_HH_ = 7.9 Hz 6H, Al(CH_2_*CH*_*3*_)_2_), 2.01 (s, 12H, *o*-*Me*_N–Ar_), 2.32 (s, 6H, *p*-*Me*_N–Ar_), 2.45 (s, 6H, *Me*–CN), 7.02 (s, 4H, *m*-CH_N–Ar_), 7.55 (s, 4H, *p*-CH_Ar_ BAr^F^_4_), 7.73, (br s, 8H, *o*-CH_Ar_ BAr^F^_4_), 8.45 (d, ^3^*J*_HH_ = 7.7 Hz, 2H, 3-CH_py_), 8.68 (d, ^3^*J*_HH_ = 7.8 Hz, 1H, 4-CH_py_).^**19**^**F{**^**1**^**H} NMR** (CD_2_Cl_2_, 25 °C, 376 MHz): δ −62.85 (s, BAr^F^_4_).^**11**^**B{**^**1**^**H} NMR** (CD_2_Cl_2_, 25 °C, 128 MHz): δ −6.61 (s, BAr^F^_4_). ^**13**^**C{**^**1**^**H} NMR** (CD_2_Cl_2_, 25 °C, 100 MHz): δ −0.51 (Al(CH_2_*C*H_3_)_2_), 8.23 (Al(*C*H_2_CH_3_)_2_), 17.26 (*Me*–CN), 18.22 (*o*-*Me*_N–Ar_), 20.88 (*p*-*Me*_N–Ar_), 117.91 (*p-C*H_Ar_ BAr^F^_4_), 123.66 (*C*F_3_ BAr^F^_4_), 126.36 (3,5-*C*H_Py_), 127.87 (*p-C*_N–Ar_), 129.30 (q, ^2^*J*_CF_ = 33 Hz, *C*-CF_3_ BAr^F^_4_), 130.37 (*m*-CH_N–Ar_), 135.22 (*o-C*H_Ar_ BAr^F^_4_), 137.99 (*o-C*_N–Ar_), 140.04 (*i-C*_N–Ar_), 146.98 (4-*C*H_Py_), 148.44 (2-*C*H_Py_), 162.20 (q, ^1^*J*_CB_ = 50 Hz, *i-C*_Ar_ BAr^F^_4_), 170.02 (Me–*C*N). **IR (KBr/Nujol, cm**^**–1**^**):** ν 1599 (intense, C=N, ^Mes^BIP); 1276, 1125, 887 (B–C for [BAr^F^_4_]^−^). **EA for C**_**63**_**H**_**53**_**AlBF**_**24**_**N**_**3**_ (found vs calculated, bulk sample): C 56.36 (56.22), H 4.16 (3.97), N 3.22 (3.12).

### Synthesis of (^Mes^BIP·)AlEt_2_ (**2d**)

A J. Young’s ampule was loaded with 325 mg (0.241 mmol) of **[1d][BAr**^**F**^_**4**_**]** and 46 mg (0.241 mmol) of Cp_2_Co. The solids were combined with 3 mL of anhydrous toluene, giving a dark red suspension. This mixture was stirred vigorously at room temperature and progress was monitored via ^1^H NMR analysis of aliquots. After 4 h, the resonances attributed to **[1d][BAr**^**F**^_**4**_**]** disappeared. Subsequent cooling of the mixture to −36 °C initiated the precipitation of [Cp_2_Co][BAr^F^_4_]. The reaction mixture was then filtered into a Schlenk tube via cannula, to which dried *n*-hexane was added until incipient crystallization occurred, and subsequently stored at −36 °C overnight. The next day, a brown/red solid was collected, isolated by cannula filtration, and dried under vacuum (<10^–2^ mbar) to yield **(**^**Mes**^**BIP·)AlEt**_**2**_**(2d)** as a red/brown solid. Yield: 70 mg, 0.145 mmol, 60%. Despite the appearance of incipient crystallization, the procedure did not yield single crystals suitable for X-ray diffraction analysis. **Note on**^**1**^**H NMR data:***The absence of*^*1*^*H NMR resonances for* (^***Mes***^***BIP·***)***AlEt***_***2***_**(2d)***is attributable to the paramagnetic nature of the compound.***EA for C**_**31**_**H**_**41**_**AlN**_**3**_ (found vs calculated, bulk sample): C 77.33 (77.14), H 8.87 (8.56), N 8.69 (8.71). **IR (KBr/Nujol, cm**^**–1**^**):** ν 1641, 1528 (medium to weak intensity, C=C Py and C=N imine). *The band observed at 1641 cm*^*–1*^*for the C*=*C stretch is characteristic of the dearomatized pyridine ring.* The **EPR** of **2d** spectrum is available in the Supporting Information.

### Redox in Situ NMR Monitoring. General Procedure

### Initial Setup

A J. Young tap NMR tube was loaded with 10 mg of the chosen aluminum complex **[^Ar’^BIPAlR**_**2**_**][BAr**^**F**^_**4**_**]**, (**1a**^**+**^**_d**^**+**^, where Ar’= Dipp and Mes and R = Me and Et). Solvents were chosen based on their ability to dissolve the reactants and products, as well as their compatibility with NMR spectroscopy. Depending on the starting complex, either 0.5 mL of CD_2_Cl_2_ (for complexes with L = ^Dipp^BIP) or Tol-*d*_8_ (for complexes with L = ^Mes^BIP) was added. The redox reaction was then in situ monitored via ^1^H NMR spectroscopy, with continuous monitoring being crucial for determining the exact point of reaction completion and observing the dynamic changes during the redox processes. The temperature was maintained at room temperature to facilitate the redox reactions without additional thermal input.

### Reduction Step

Before the addition of the reductant, a ^1^H NMR experiment was acquired for each aluminum complex **1a**^**+**^**–d**^**+**^ to establish a baseline. An equimolar quantity of Cp_2_Co was then added to the corresponding aluminum complex solution. The solution was mixed using a bespoke NMR tube rotor at room temperature, with the progress and completion of the reduction monitored by ^1^H NMR analyses. After 20 min, the reduction of all aluminum complexes was confirmed by the disappearance of ^1^H NMR resonances for **1a**^**+**^**–d**^**+**^ attributable to the paramagnetic NMR-silence nature of the reduced compound **[(^Ar’^BIP·)AlR**_**2**_**]** (**2a**–**d**) with the only signals being those of [Cp_2_Co][BAr^F^_4_].

### Oxidation Step

Following the reduction of each aluminum complex to the corresponding **2a**–**d**, species, an equimolar amount of [Fc][PF_6_] was added. The mixture was then mixed and monitored by ^1^H NMR analyses. After 20 min, the oxidation of the aluminum species was evidenced by the reappearance of ^1^H NMR resonances for the species **1a**^**+**^**–d**^**+**^ along with resonances attributable to Fc and species [Cp_2_Co]^+^.

### Notes on Alternative Reducing and Oxidizing Agents

Further experiments tested the redox behavior using Ferrocene (Fc) and Cp_2_Co as alternative reducing agents, with only Cp_2_Co successfully reducing the aluminum complexes **1a**^**+**^**–d**^**+**^. Attempts to oxidize the **2a**–**d** species with [Fc][BF_4_] failed. THF-*d*_8_ was also trialed as a solvent; however, it led to degradation of the reduced aluminum species, resulting in complex reaction mixtures.

### Single-Crystal X-ray Analysis

A summary of the crystallographic data and the structure refinement results for compounds **[1a][BAr**^**F**^_**4**_**]**, **[1c][PF**_**6**_], **[1d][BAr**^**F**^_**4**_**]**, **[Cp**_**2**_**Co][BAr**^**F**^_**4**_**]** and **2b** is given in Tables S1–S5 in the Supporting Information. Crystals of a suitable size for X-ray diffraction analysis were coated with dry perfluoropolyether and mounted on glass fibers and fixed in a cold nitrogen stream (*T* = 193 K) to the goniometer head. Data collection was carried out on a Bruker-AXS, D8 QUEST ECO, PHOTON II area detector diffractometer, using monochromatic radiation λ(Mo K_α_) = 0.71073 Å, by means of ω and φ scans with a width of 0.50°. The data were reduced (SAINT^34^) and corrected for absorption effects by the multiscan method (SADABS^35^). The structures were solved by intrinsic phasing modification of direct methods (SHELXT^36^) and refined against all *F*^2^ data by full-matrix least-squares techniques (SHELXL-2018/3^37^) minimizing *w*[*F*_o_^2^ – *F*_c_^2^]^2^. All non-hydrogen atoms were refined anisotropically. The hydrogen atoms were included from calculated positions and refined riding on their respective carbon atoms with isotropic displacement parameters. In general, the –CF_3_ groups of BAr^F^_4_ in **[1a][BAr**^**F**^_**4**_**]** present positional disorder, so five of them were modeled as two components of the disorder with their respective occupancy coefficients. Therefore, it was also necessary to use some geometric restraints (SADI, SIMU) during the structure refinement to ensure a sensible geometry. In the asymmetric unit of [**1c][PF**_**6**_], a crystallizing THF molecule that did not need to be modeled is shown next to the salt complex. A search for solvent-accessible voids for structure **[1a][BAr**^**F**^_**4**_**]** using SQUEEZE^38^ showed two volumes of potential solvents of 190 Å^3^ for each (52 electron count), whose solvent content could not be identified or refined with the most severe restrictions, but due to the volume and the electrons present, it would match a very disordered *n*-hexane molecule. While a search for solvent-accessible voids for the [**1c][PF**_**6**_] structure using SQUEEZE showed a single potential solvent volume of 292 Å3 (70 electron count), whose solvent content could not be identified or refined under the most severe constraints, but due to the volume and electrons present, it would match one and a half molecules of very disordered *n*-hexane. The corresponding CIF data represent SQUEEZE treated structures with the solvent molecules handling as a diffuse contribution to the overall scattering, without specific atom position and excluded from the structural model. The SQUEEZE results were appended to the CIF. Crystallizing compounds **[1a][BAr**^**F**^_**4**_**]** and **[1c][PF**_**6**_**]** is challenging due to their instability, and they appear somewhat twinned, exhibiting at least four additional minority domains. Refining these structures using HKLF 5 and BASF did not yield improved results. Consequently, due to the imperfections in the crystals and the presence of some disorder, as previously discussed and resolved, all the R-values are somewhat elevated. Single-crystal X-ray diffraction data for **[Cp**_**2**_**Co][BAr**^**F**^_**4**_**]** and **2b** were obtained under a nitrogen gas stream using an Oxford Cryostream 800 unit^39^ at the specified temperature with a Bruker D8 Quest Eco diffractometer equipped with a Photon II 7 detector (Mo, λ = 0.71073 Å). The raw frame data was reduced using APEX3. Structures were resolved using SHELXT and refined using full-matrix least-squares refinement on all *F*^2^ data using the SHELXL^40^ interface within GUI OLEX2.^41^ Unless stated otherwise, all non-hydrogen atoms were refined anisotropically, while hydrogen atoms were geometrically placed and allowed to ride on their parent atoms. Disorder was addressed by implementing a split-site model and restraining geometries and displacement parameters. In several structures some of the CF_3_ groups on the BAr^F^_4_^–^ anion was disordered and modeled over two main domains, and restrained to maintain sensible geometries. Distances and angles were calculated using the full covariance matrix. The corresponding crystallographic data were deposited with the Cambridge Crystallographic Data Centre as supplementary publications. CCDC 2337930 (**[1a][BAr**^**F**^_**4**_**]**), 2337931 (**[1c][PF**_**6**_**]**), 2334613 ([**1d][BAr**^**F**^_**4**_**]**) 2326416**[Cp**_**2**_**Co][BAr**^**F**^_**4**_**]** and 2326417 for **2b** contain the supplementary crystallographic data for this paper. The data can be obtained free of charge via*:*https://www.ccdc.cam.ac.uk/structures/.

## Supporting Information Available

Instrumentation description, characterization data including NMR, IR and EPR spectra, EA, crystallographic files (CIF), cyclic voltammetry (CV), and computational data (PDF)
