# Supplementary material for: Reversible Redox Ligand-Centered Reactivity in 2,6-Bisiminopyridine Aluminum Systems
Source: Inorg Chem. 2024 Oct 4;63(41):19156–66. doi: 10.1021/acs.inorgchem.4c02664 (PMC11483753; doi:10.1021/acs.inorgchem.4c02664)
Supplement: Supplementary file 1 — ic4c02664_si_002.pdf [file ic4c02664_si_002.pdf]

Supporting Information for

Reversible Redox Ligand-Centered Reactivity in 2,6-Bisiminopyridine Aluminum Systems

Juan Manuel Delgado-Collado,[†,a] Hellen Videa,[†,b] Pablo. J. Serrano-Laguna,[†,b], M. Ángeles Fuentes,[b] Eleuterio Álvarez,[a] Antonio Díaz Quintana,[a] Antonio J. Martínez-Martínez[b,*], Antonio Rodríguez-Delgado [a,*] and Juan Cámpora.[a,*]

[a] *Instituto de Investigaciones Químicas, CSIC-Universidad de Sevilla, c/Américo Vespucio, 49, 41092 Sevilla, Spain*

[b] *CIQSO-Center for Research in Sustainable Chemistry and Department of Chemistry, CSIC-Associated Unit, University of Huelva, Campus El Carmen, 21007 Huelva, Spain*

Contents

1. Instrumentation and procedures S2-S3
2. NMR and IR spectra of new compounds S4-S22
3. Redox In-situ monitoring spectra S23-S26
4. Cyclic voltammetry (CV) of **1a**$^+$-**d**$^+$ and $[H^{DiPP}BIP][BAr^F{}_4]$ S27-S33
5. EPR of **2a-d** S34-S35
6. X-ray structural characterization S36-S47
7. Computational details S46-S58
8. References S58-S58

1. Instrumentation and procedures

NMR Spectroscopy: NMR spectra were recorded on Bruker equipment, models DPX-300, DPX-400, AVIIIHD 400 nanobay or a AVIII 500, typically at 298 K, unless stated otherwise. The chemical shifts of the ^{1}H and $^{13}C\{^{1}H\}$ spectra are referenced to tetramethylsilane (TMS), whereas the shifts of the $^{19}F\{^{1}H\}$ spectra are expressed with regard to $CFCl_3$. Spectral assignations were routinely helped with bidimensional (2D) ^{1}H-^{1}H COSY, ^{1}H-^{13}C HMBC and HSQC heterocorrelation spectra. $^{19}F\{^{1}H\}$ NMR spectra used external referencing with 1% $CFCl_3$ sample in $CHCl_3$. $^{11}B\{^{1}H\}$ NMR spectra were externally referenced to 5% $BF_3{\cdot}OEt_2$ sample in C_6D_6. Chemical shifts (δ) are reported in ppm and coupling constants (J) in Hz. The resonances corresponding to the $[BAr^{F}{}_4]^{-}$ counterion are omitted due to their consistent single resonance at δ -62.9 and -6.5 in the ^{19}F and ^{11}B NMR spectra, respectively. Prior use, CD_2Cl_2 was dried over CaH_2, filtered, and distilled under reduced pressure. Deuterated toluene (Tol-d_8) was dried using CaH_2, distilled under low pressure ($< 1 \times 10^{-2}$ mbar), and stored under nitrogen over a potassium mirror. Anhydrous deuterated tetrahydrofuran (THF-d_8) was dried using CaH_2, distilled under low pressure ($< 1 \times 10^{-2}$ mbar) and stored under nitrogen over Na mirror.

Infrared Spectroscopy: Infrared spectra were recorded on a Bruker Vector 22 and on a Bruker Alpha FT-IR spectrometer and sampling preparation was made in Nujol mulls between KBr discs, mounted in a nitrogen-filled glove box.

Elemental Analysis: CHN microanalyses were executed in a LECO TruSpec CHN elementary analyzer in the Instituto de Investigaciones Químicas (CSIC-Universidad de Sevilla)

X-ray diffraction studies: These measurements were carried in a Bruker-AXS, D8 Quest ECO diffractometer equipped with a micro-focus IμS 3.0 source, using graphite monochromatized Mo radiation λ(Mo Kα) = 0.71073 Å and with an area detector Bruker Photon II 14 - CPAD in the Instituto de de Investigaciones Químicas (CSIC-Universidad de Sevilla) and in an Oxford Cryostream 800 unit with a Bruker D8 Quest Eco diffractometer equipped with a Photon II 7 detector (Mo, λ = 0.71073 Å), in the CIQSO-Center for Research in Sustainable Chemistry and Department of Chemistry Universidad de Huelva, respectively.

EPR spectroscopy: Electronic Paramagnetic Resonance spectra were recorded with a Bruker Elexsys spectrometer working in X-Band (~ 9.47 GHz) equipped with a helium

gas-flow cryostat. EPR samples were dissolved in toluene under a nitrogen atmosphere and transferred to quartz tubes that were subsequently sealed. Experimental cw-EPR data were fit using EasySpin (version 5.2.35) and MATLAB R2023a package.[1] The spin system (S = ½) was assumed to be under isotropic fast motion regime. Hyperfine couplings from an ^{27}Al, three ^{14}N and several ^{1}H atoms —according to the specific molecular structure— were considered. The initial guess for computations were previously reported estimations of g_{iso} and hyperfine coupling constants (A).[2] Voigt functions were used to fit line width broadenings; the contribution of Gaussian and Lorentzian shapes being allowed to vary during the latest iterations. As previously reported, hydrogen atoms belonging to the two imine methyl groups were considered equivalent.[2] The same approach was used for the methyl or ethyl groups bound to Al. For DFT-based computations of EPR parameters, see Computational Details in Supplementary Information.

Electrochemistry: Cyclic voltammetry experiments were carried out using a Metrohm Autolab PGSTAT204 potentiostat. Glassy carbon working (⌀ = 3 mm) and non-aqueous silver/silver chloride pseudoreference electrodes were obtained from CH Instruments. The counter electrode was a glassy carbon rod (⌀ = 3 mm) from ALS. CV experiments were performed in a 20 mL cell with custom-made ports for all electrodes. Tetrabutylammonium hexafluorophosphate was purified by recrystallization from ethanol and dried under vacuum before being stored in a desiccator. All data were referenced to an internal ferrocene standard.

2. NMR spectra of new compounds

[^{DiPP}BIPH][Cl]

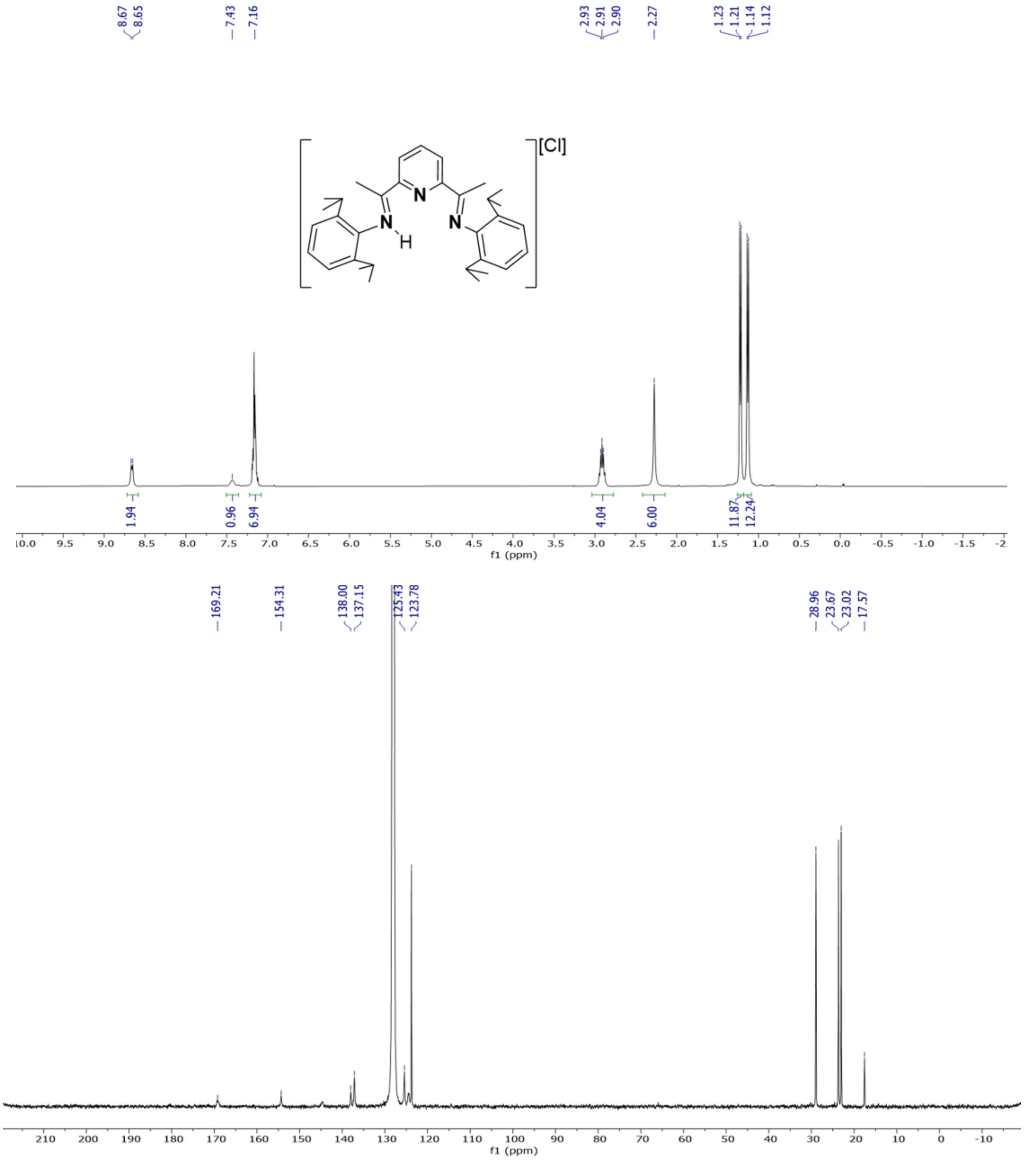

Figure S1. **^{1}H-** (C_6D_6, 25 °C, 400 MHz) and **^{13}C{^{1}H}-NMR** (100 MHz) spectra of **[^{DiPP}BIPH][Cl].**

[^{DiPP}BIPH][PF$_6$]

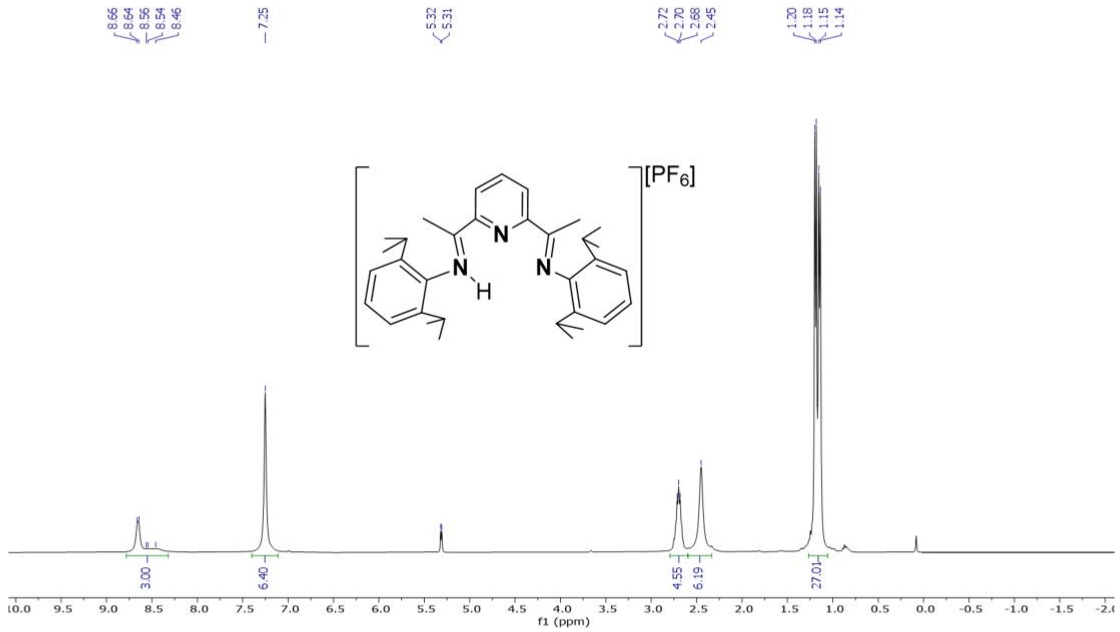

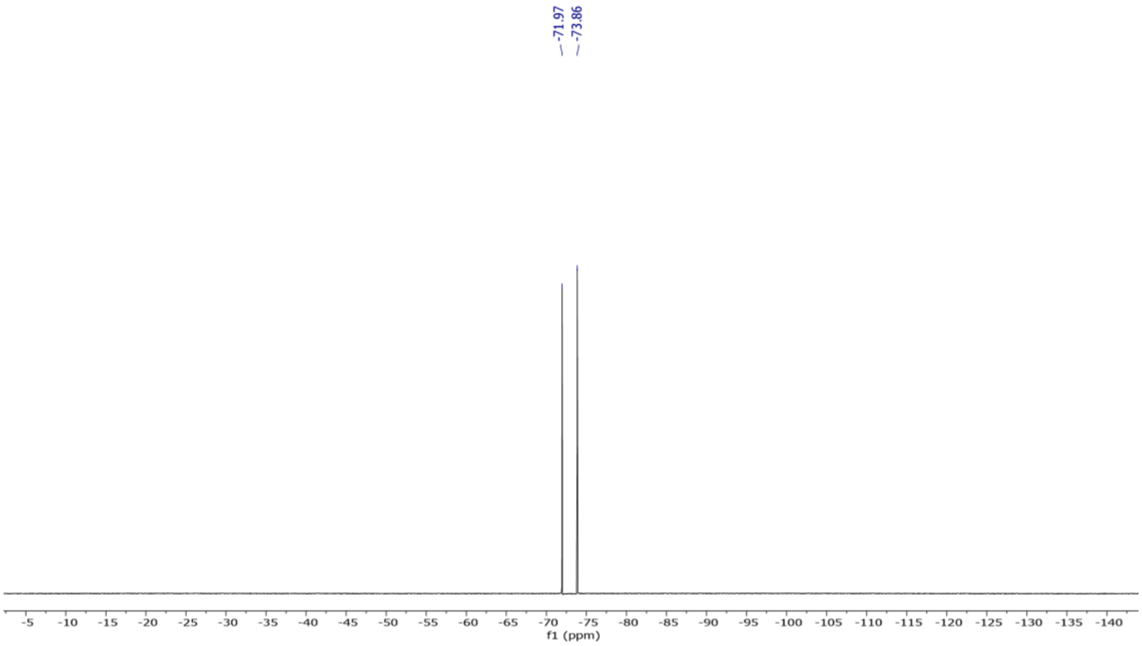

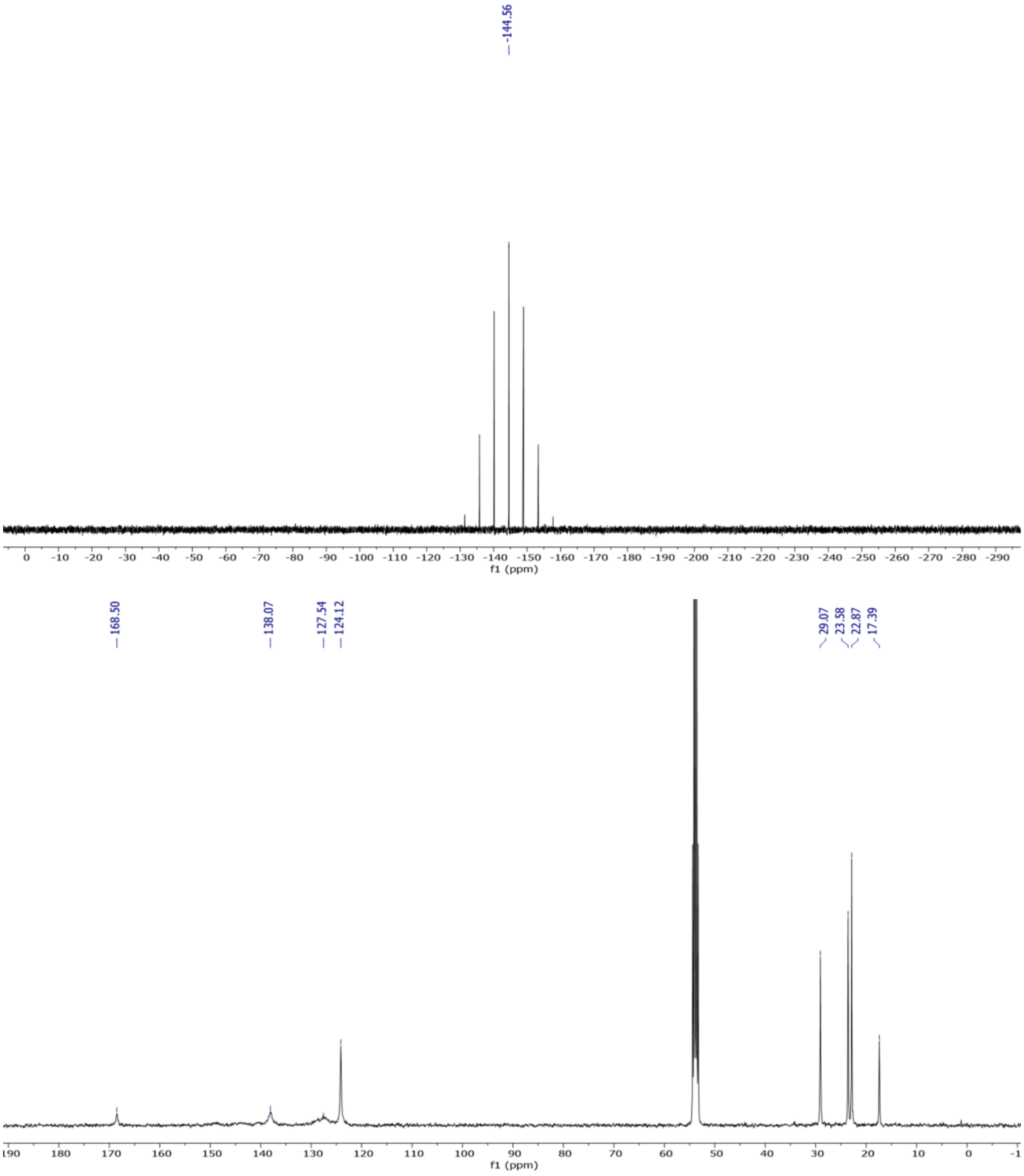

Figure S2. ^{1}**H-** (CD_2Cl_2, 25 °C, 400 MHz) ^{19}**F{**^{1}**H}-** (376 MHz) ^{31}**P{**^{1}**H}- (**162 MHz) and ^{13}**C{**^{1}**H}-NMR** (100 MHz) spectra of **[**^{DiPP}**BIPH][**PF_6**].**

[Al(Me)$_2$(^{DiPP}BIP)][PF$_6$] ([1a][PF$_6$])

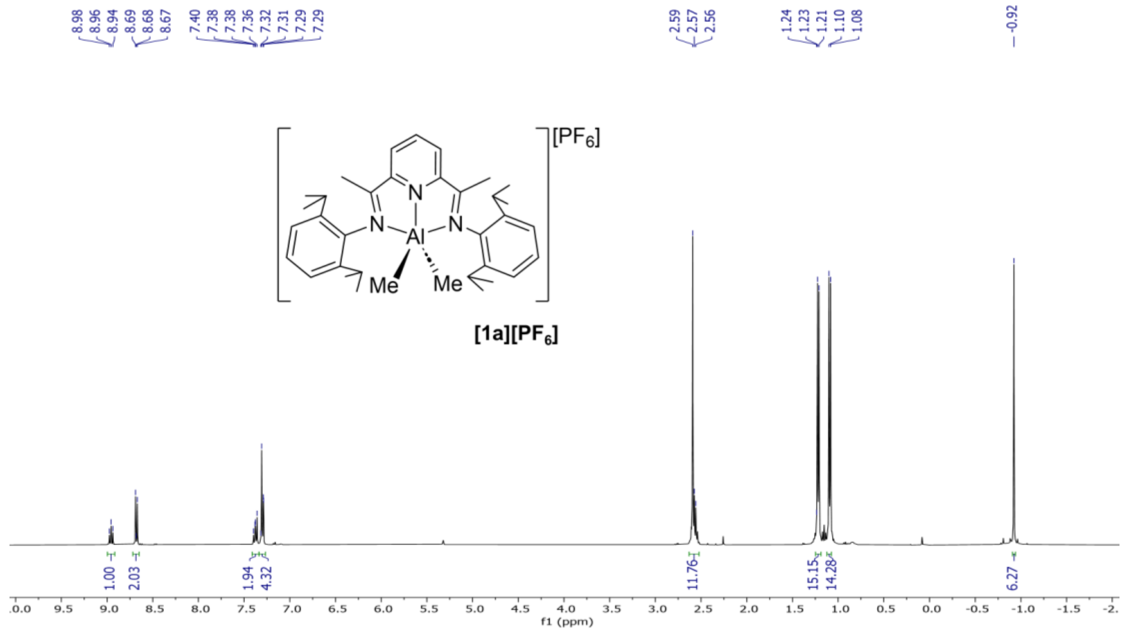

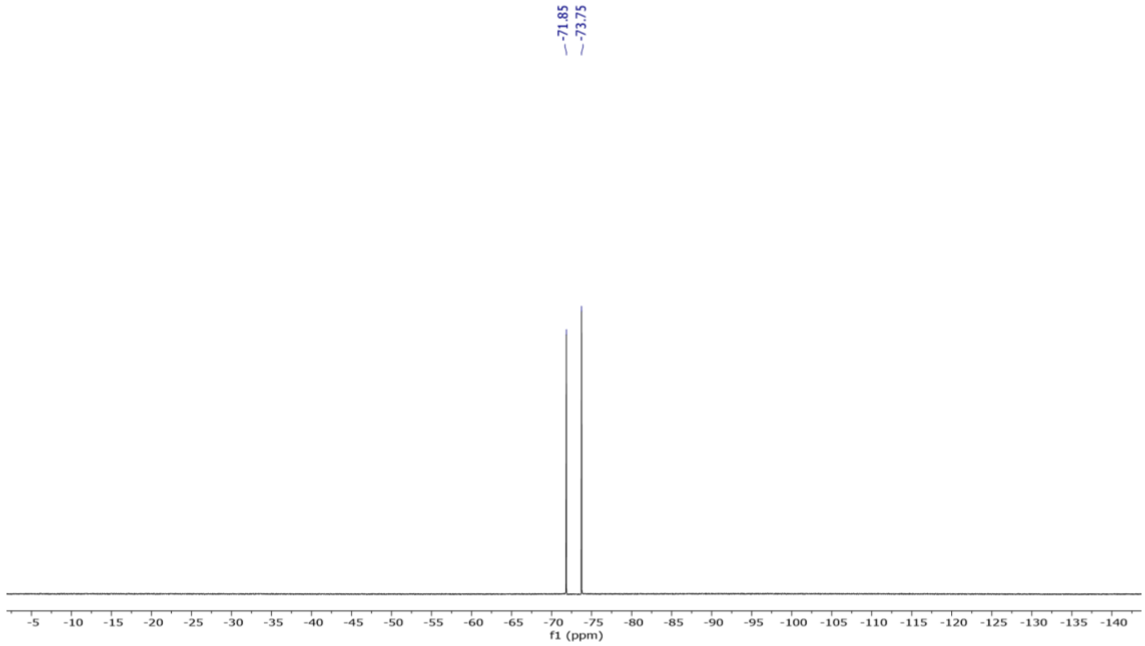

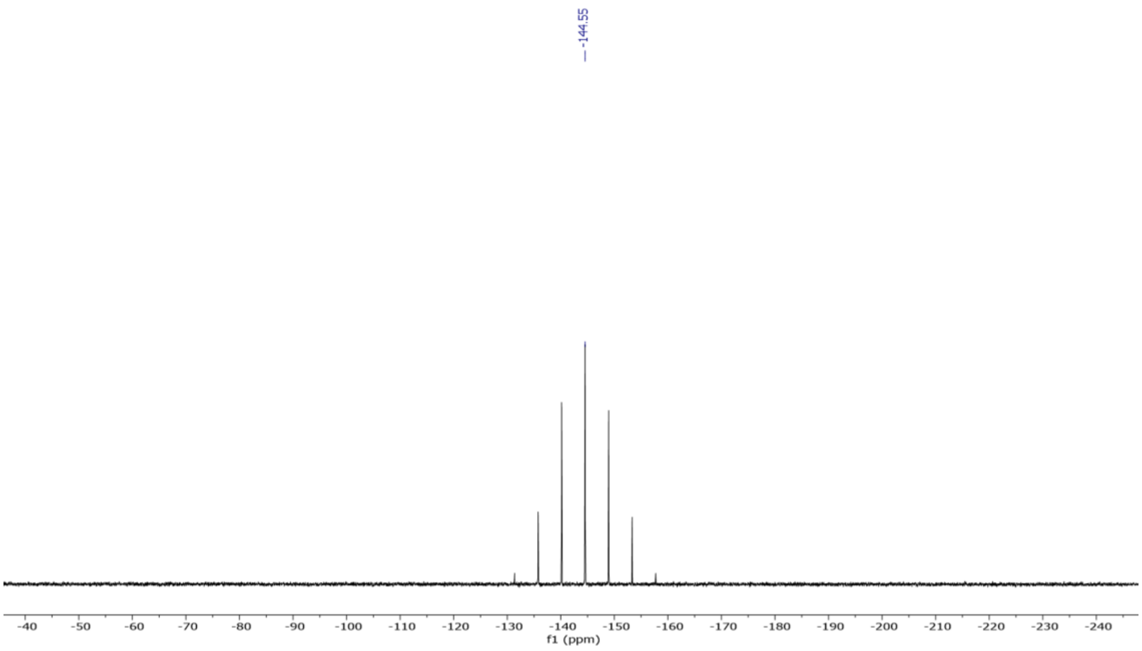

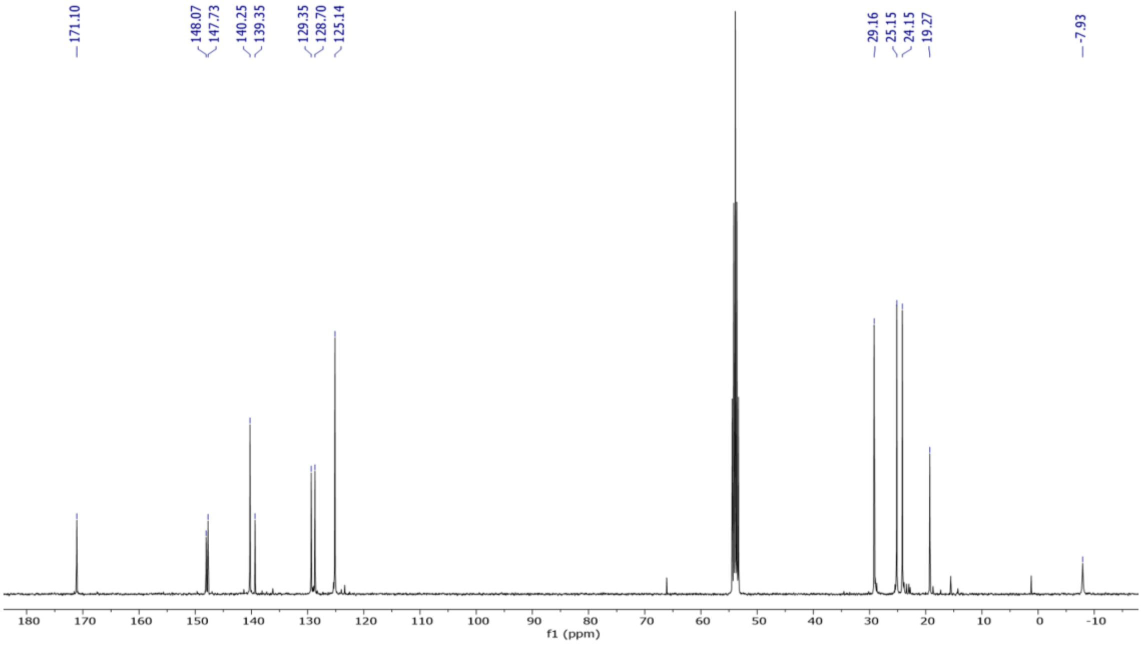

Figure S3. **^{1}H-** (CD_2Cl_2, 25 °C, 400 MHz) **^{19}F{^{1}H}-** (376 MHz) **^{31}P{^{1}H}-** (162 MHz) and **^{13}C{^{1}H}-NMR** (100 MHz) spectra of **[1a][PF_6].**

[Al(Me)$_2$(^{DiPP}BIP)][BAr$^F_4$] ([1a][BAr$^F_4$])

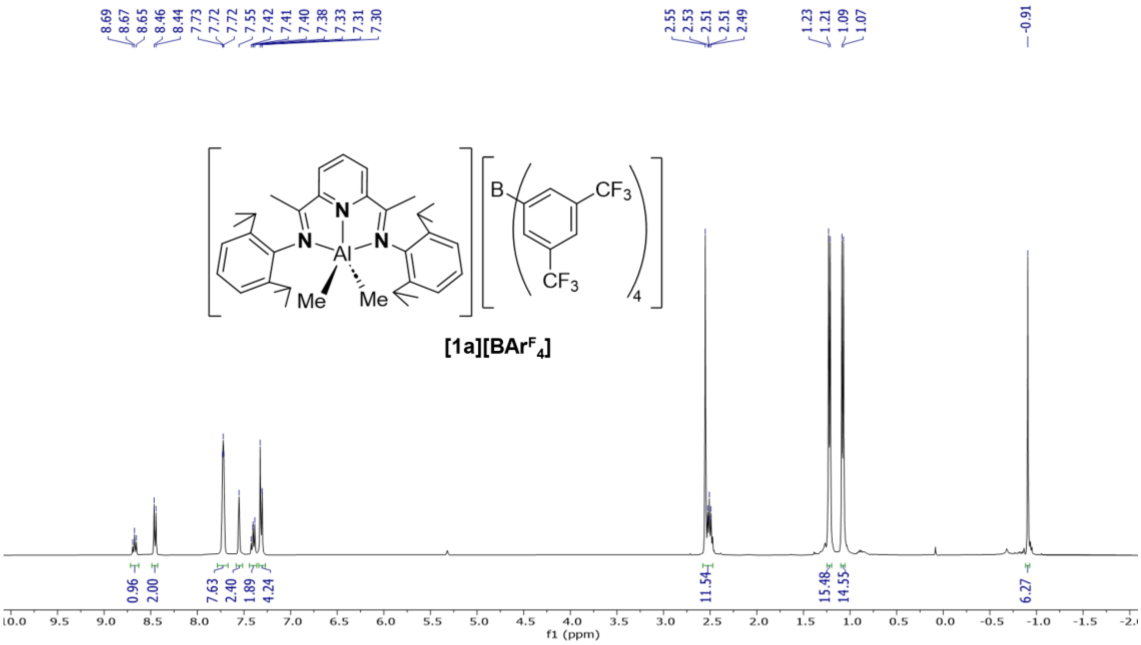

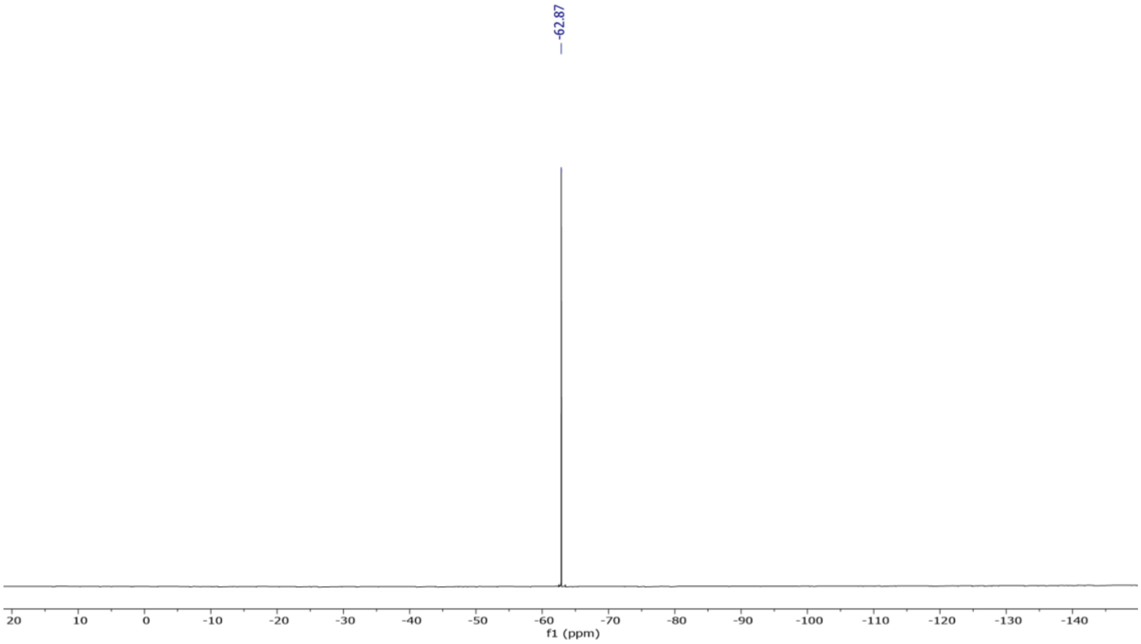

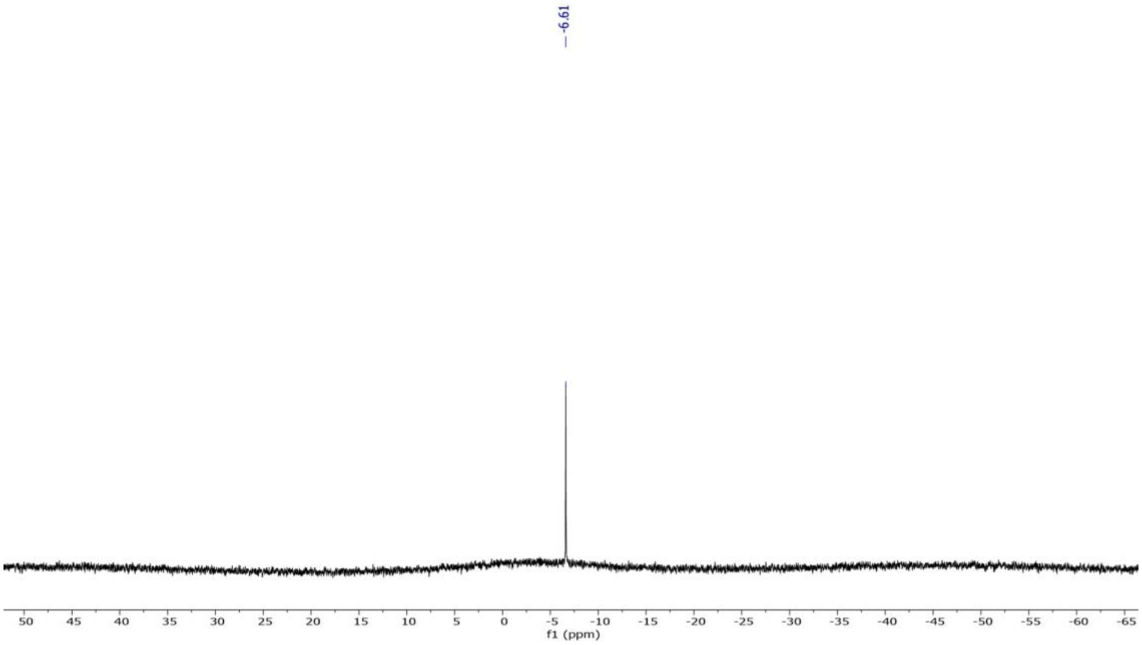

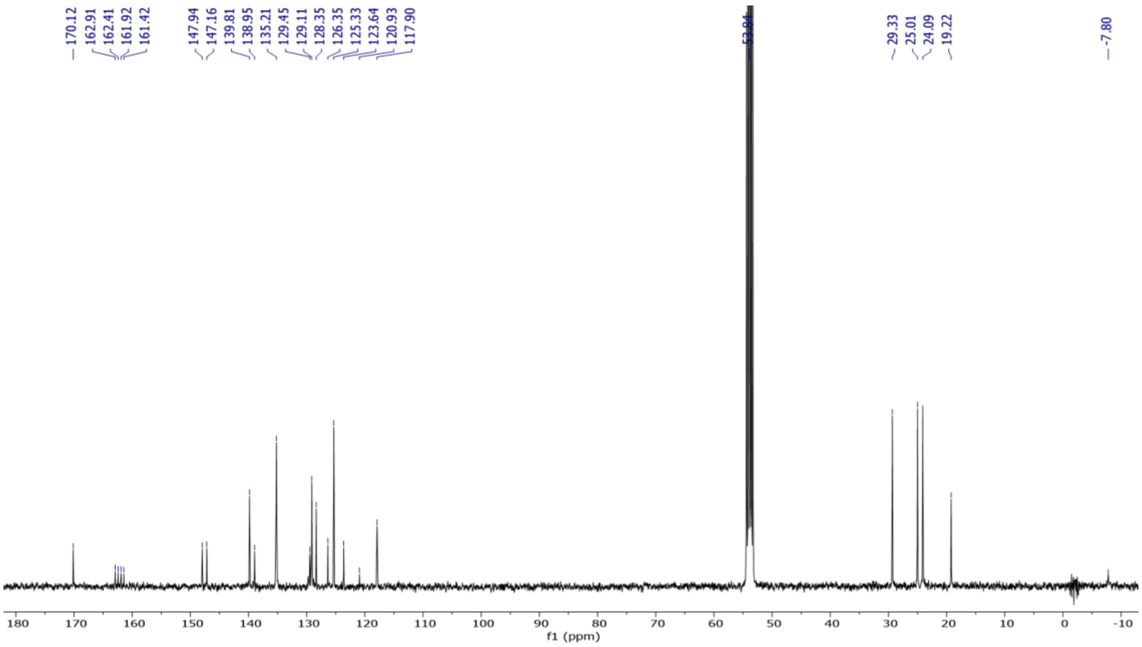

Figure S4. **^{1}H-** (CD_2Cl_2, 25 °C, 400 MHz), **^{19}F{^{1}H}-** (376 MHz), **^{11}B{^{1}H}-** (128 MHz) and **^{13}C{^{1}H}-NMR** (100 MHz) spectra of **[1a][BAr^{F}$_4$]**.

(^{DiPP}BIP·)AlMe$_2$ (2a)

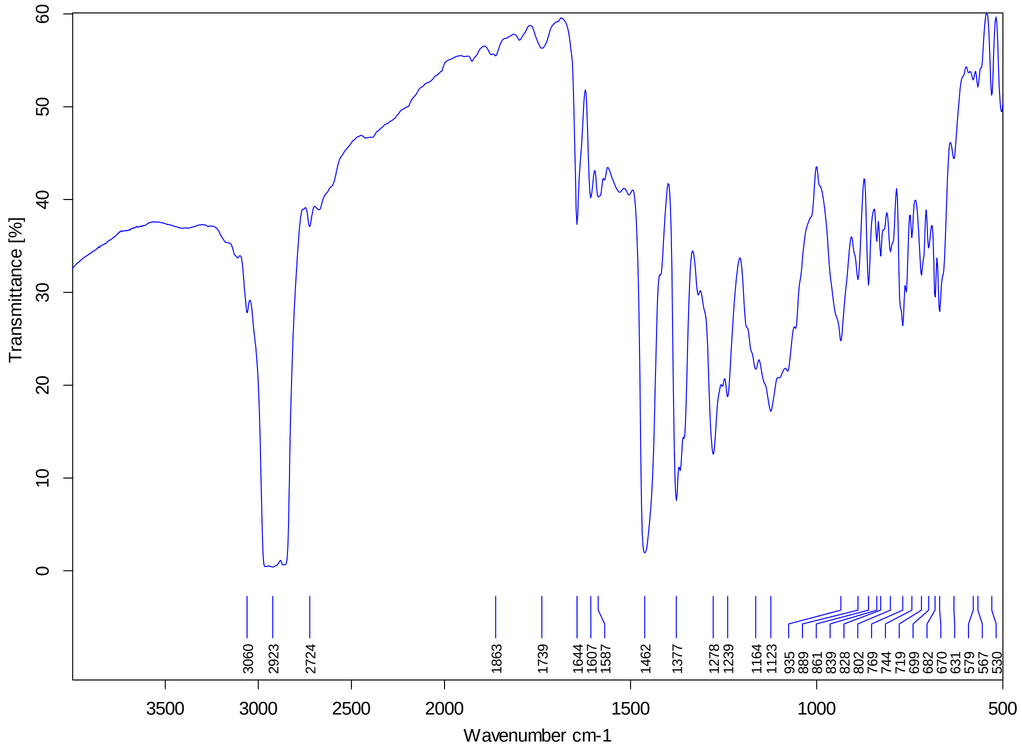

Figure S5. **IR (KBr/Nujol)** spectrum of **(^{DiPP}BIP·)AlMe$_2$ (2a).**

[Al(Me)$_2$(^{Mes}BIP)][BAr$^F_4$] ([1b][BAr$^F_4$])

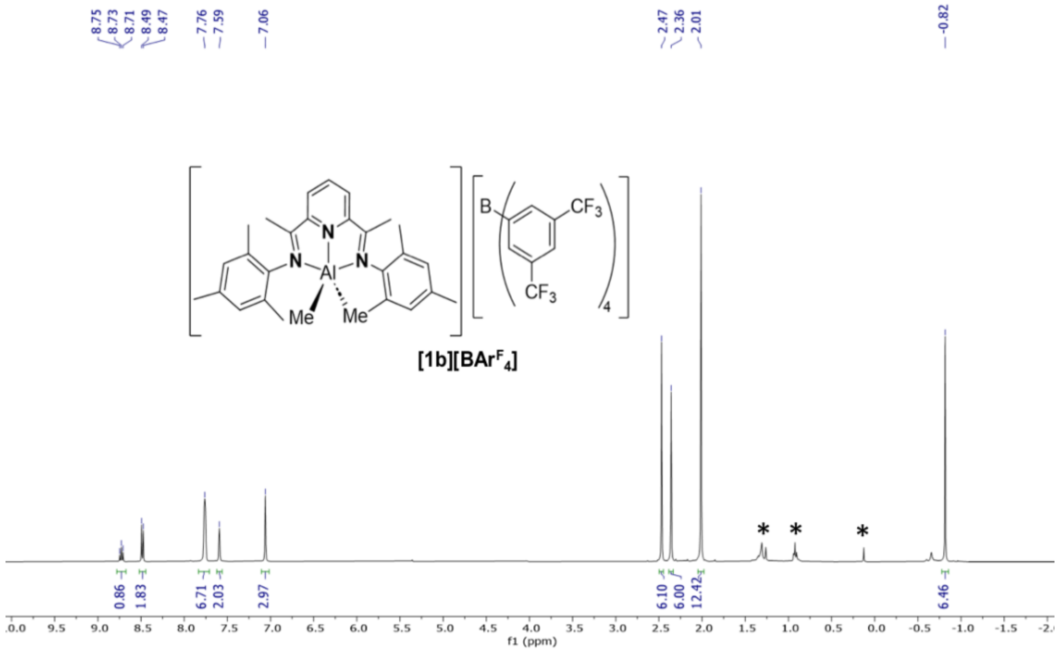

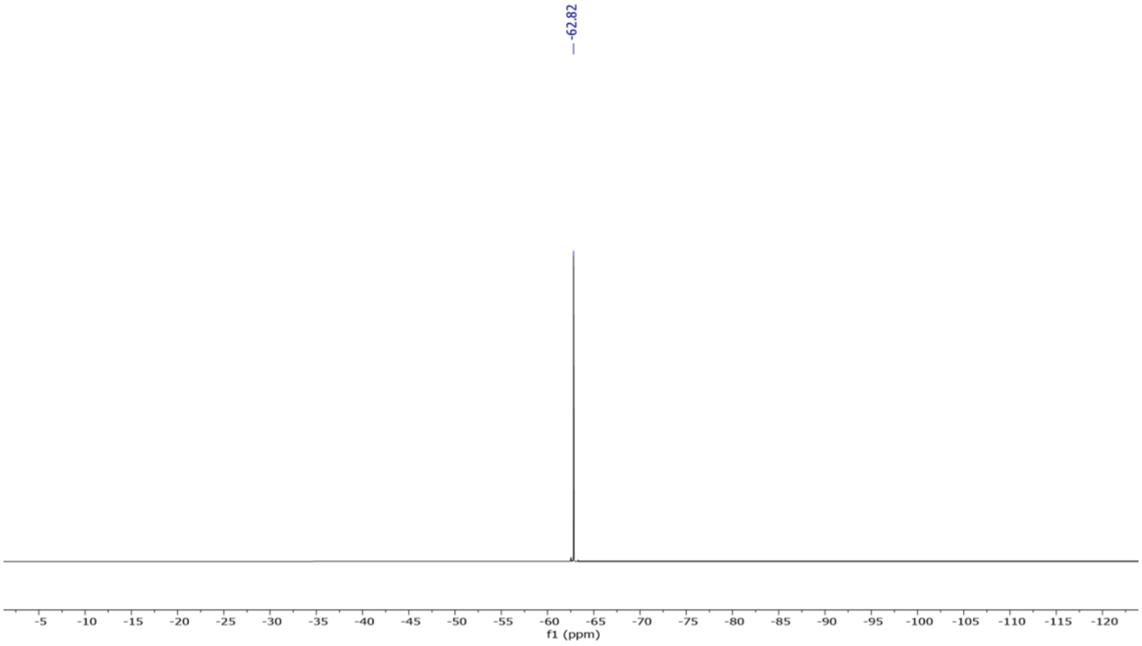

-62.82
f1 (ppm)

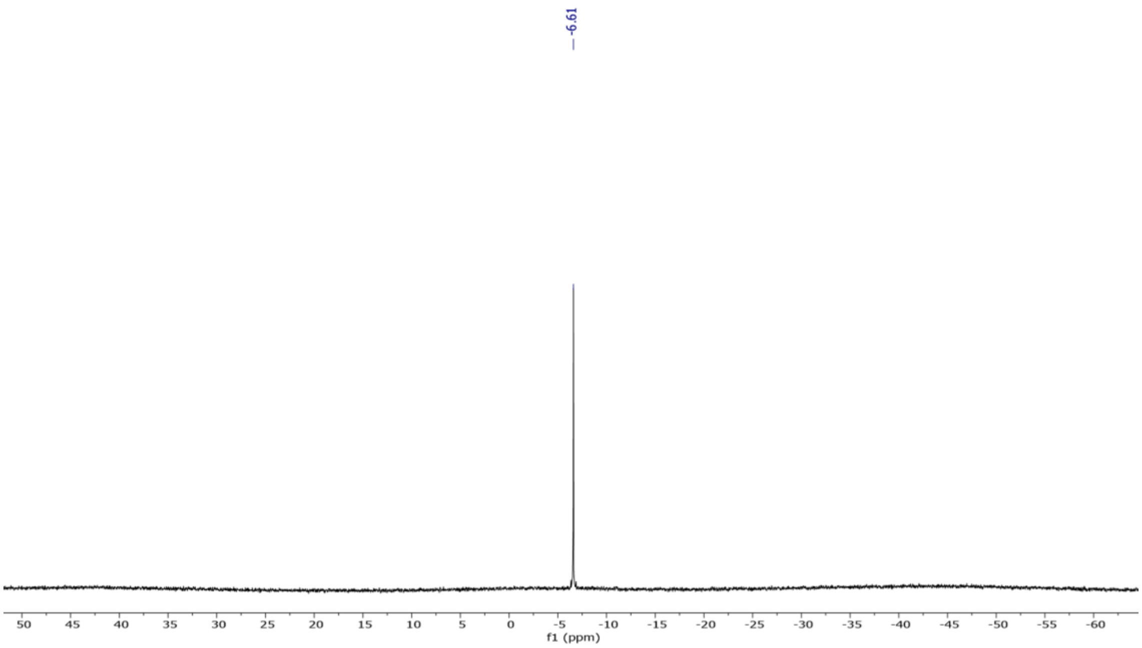

-6.61
f1 (ppm)

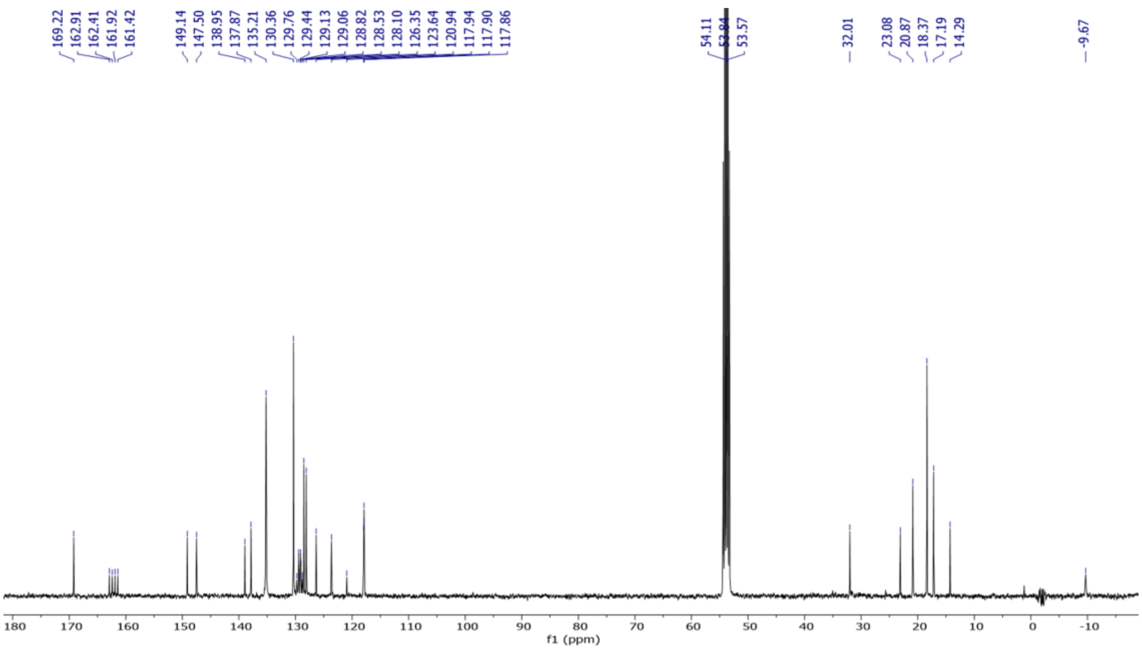

Figure S6. **^{1}H-** (CD_2Cl_2, 25 °C, 400 MHz), **^{19}F{^{1}H}-** (376 MHz), **^{11}B{^{1}H}-** (128 MHz) and **^{13}C{^{1}H}-NMR** (100 MHz) spectra of **[1b][BAr^F$_4$]**. Resonances marked with asterisks in the ^{1}H-NMR were assigned for remaining traces of some of the solvents (hexane) used in the recrystallization process and impurities (grease), respectively.

(^{Mes}BIP·)AlMe$_2$ (2b)

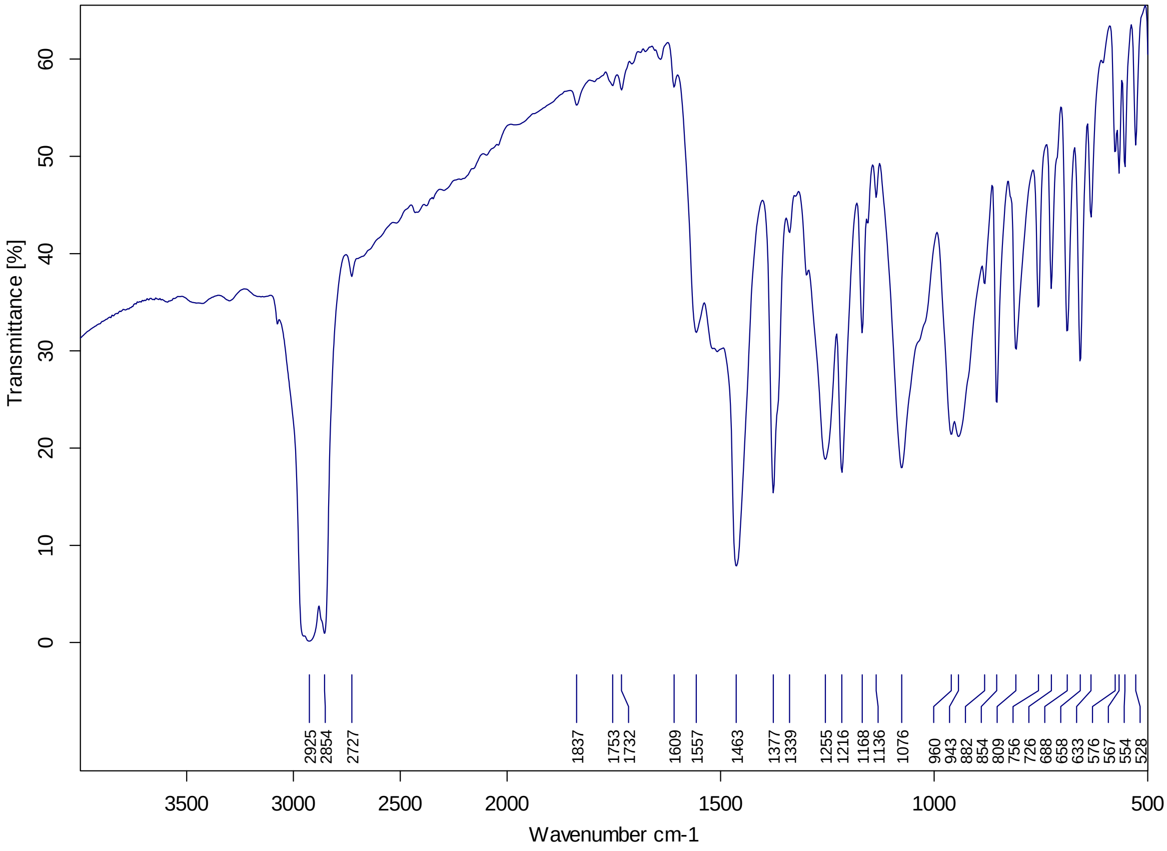

Figure S7. **IR (KBr/Nujol, cm^{-1})** spectrum of **(^{Mes}BIP·)AlMe$_2$ (2b).**

[Al(Et)$_2$(^{DiPP}BIP)][BAr^{F}$_4$] [1c][BAr^{F}$_4$]

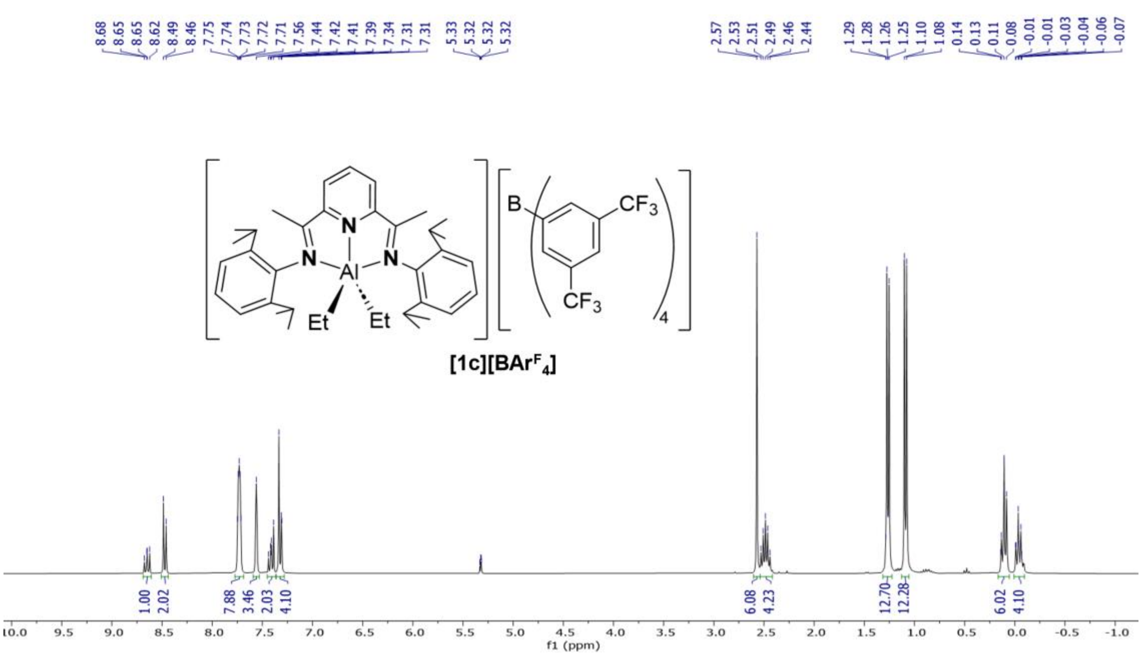

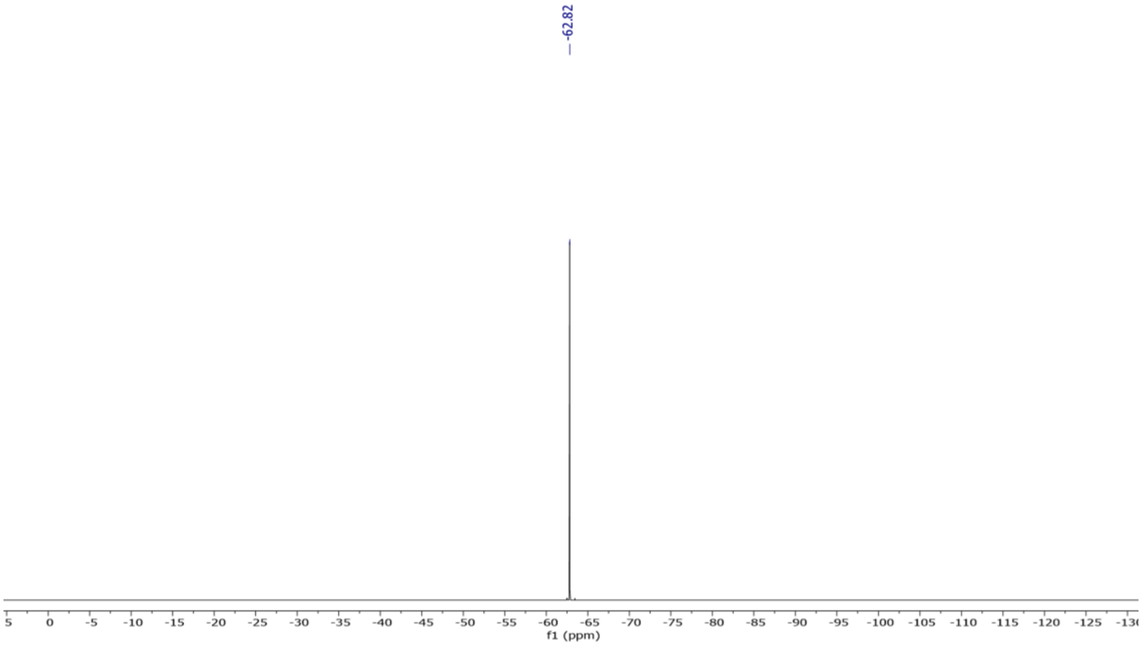

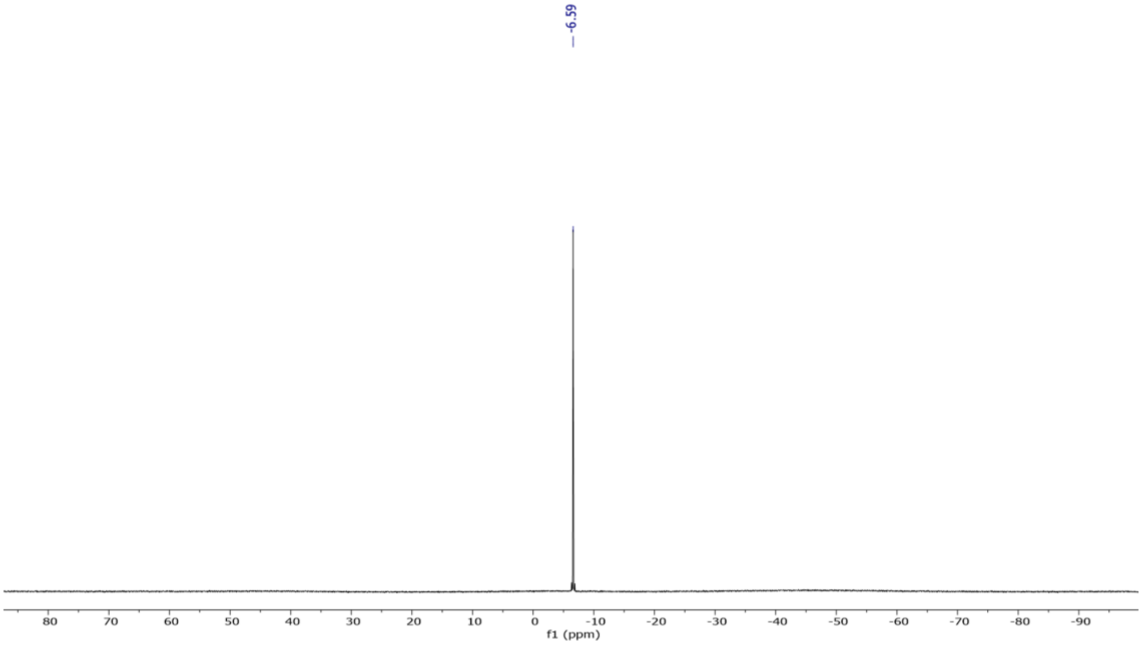

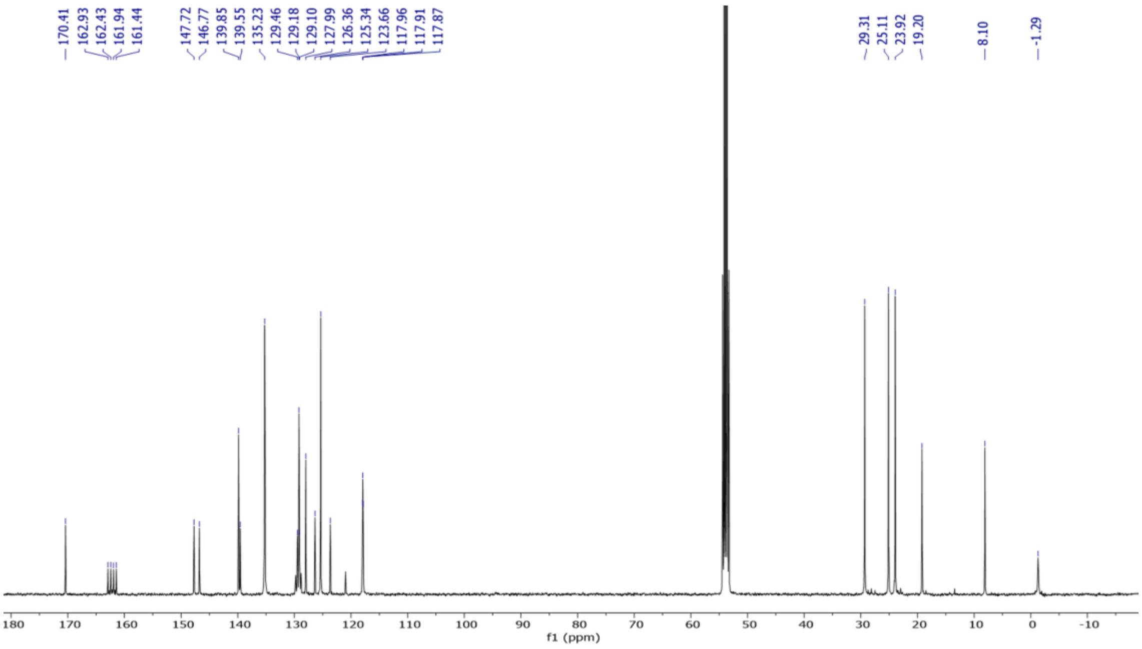

Figure S8. **^{1}H-** (CD_2Cl_2, 25 °C, 400 MHz), **^{19}F{^{1}H}-** (376 MHz), **^{11}B{^{1}H}-** (128 MHz) and **^{13}C{^{1}H}-NMR** (100 MHz) spectra of **[1c][BAr$^{F}_{4}$].**

$[Al(Et)_2(^{DiPP}BIP)][PF_6]$ ($[1c][PF_6]$)

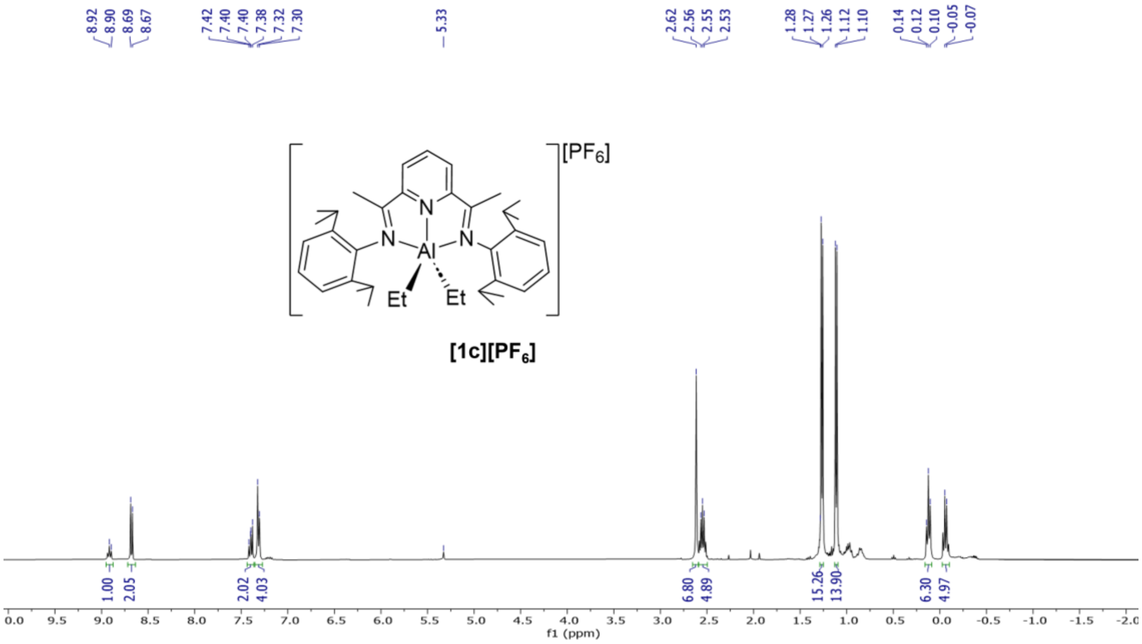

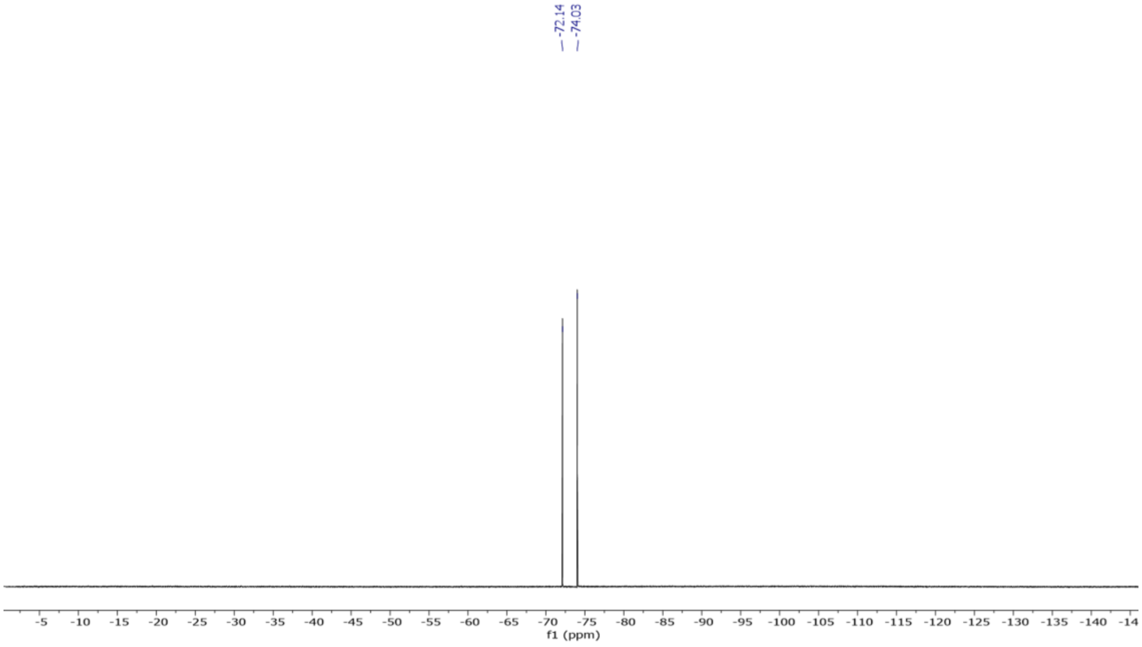

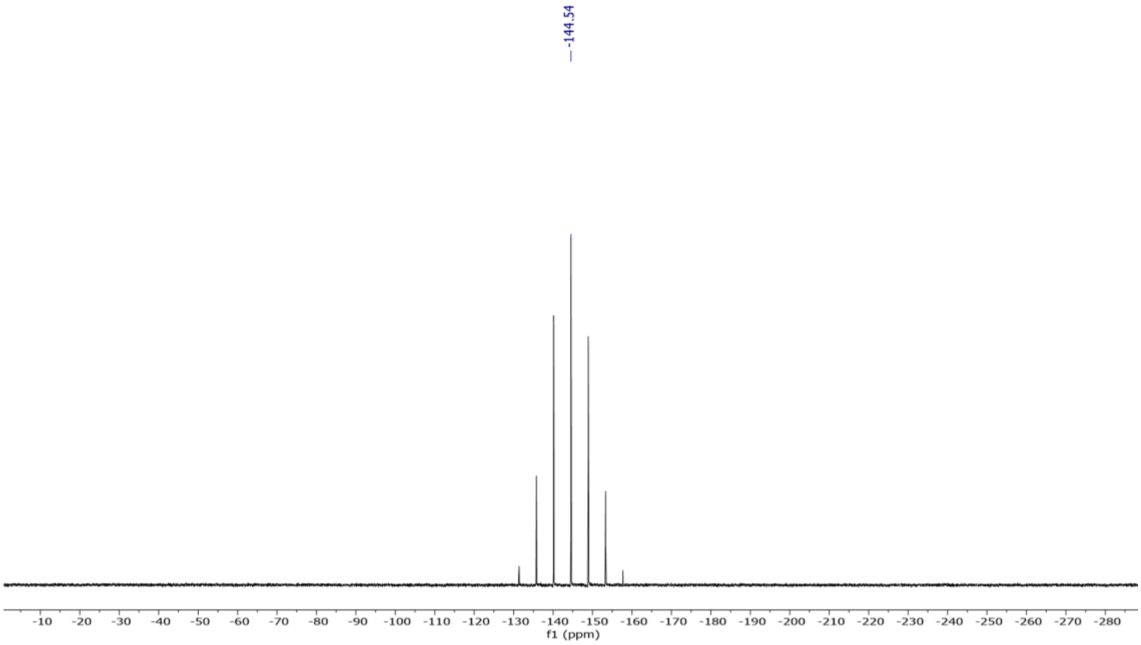

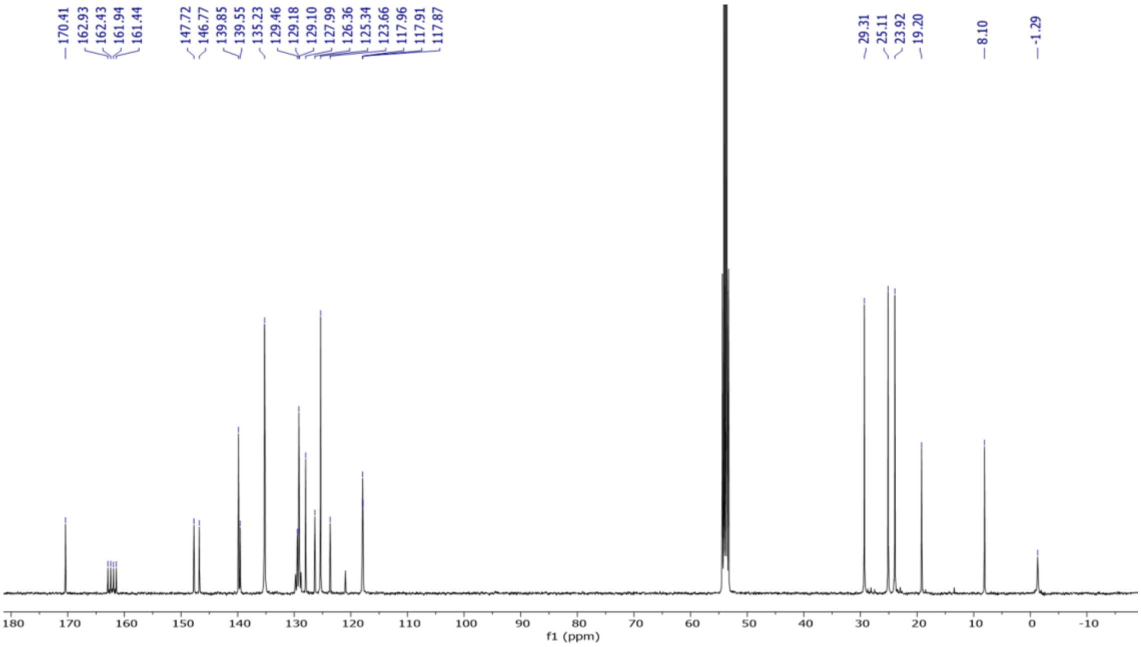

Figure S9. **^{1}H-** (CD_2Cl_2, 25 °C, 400 MHz), **^{19}F{^{1}H}-** (376 MHz), **^{31}P{^{1}H}-** (162 MHz), **^{13}C{^{1}H}-NMR** (100 MHz) spectra of **[1c][PF$_6$].**

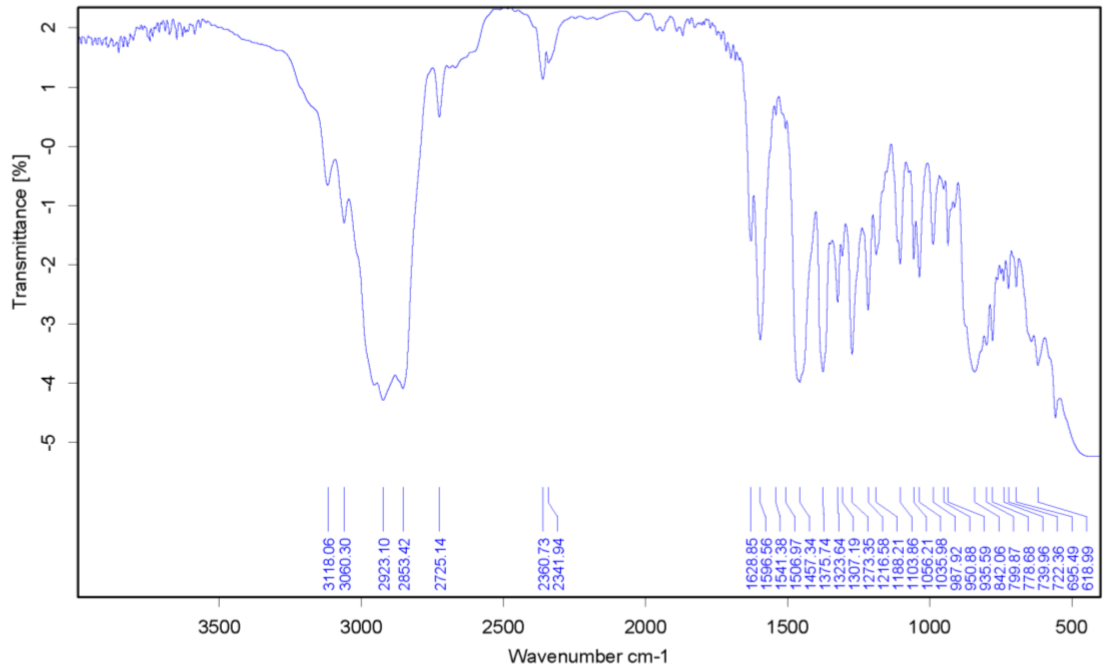

Figure S10. **IR (KBr/Nujol, cm^{-1})** spectrum of **[1c][PF_6].**

(^{DiPP}BIP·)$AlEt_2$ (2c)

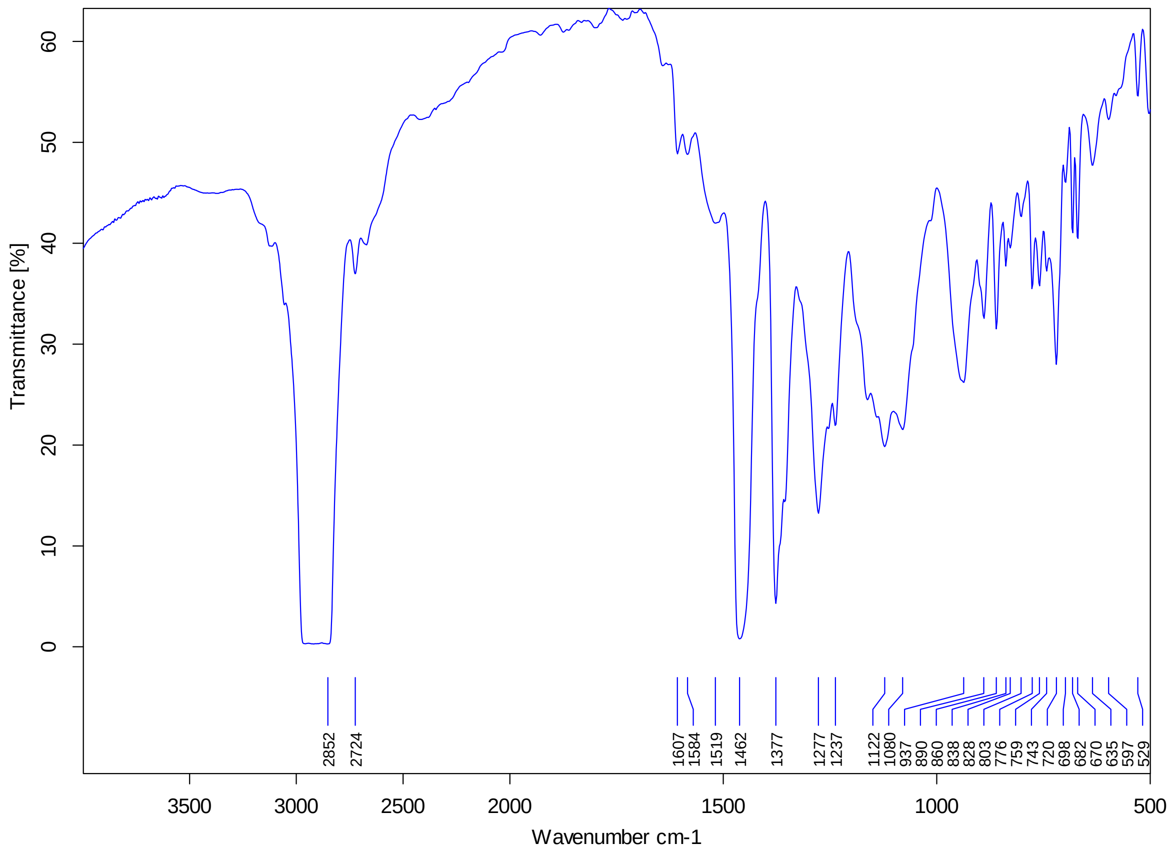

Figure S11. IR (KBr/Nujol, cm^{-1}) spectrum of **(^{DiPP}BIP·)$AlEt_2$ (2c).**

[Al(Et)$_2$(^{Mes}BIP)][BAr$^F_4$] ([1d][BAr$^F_4$])

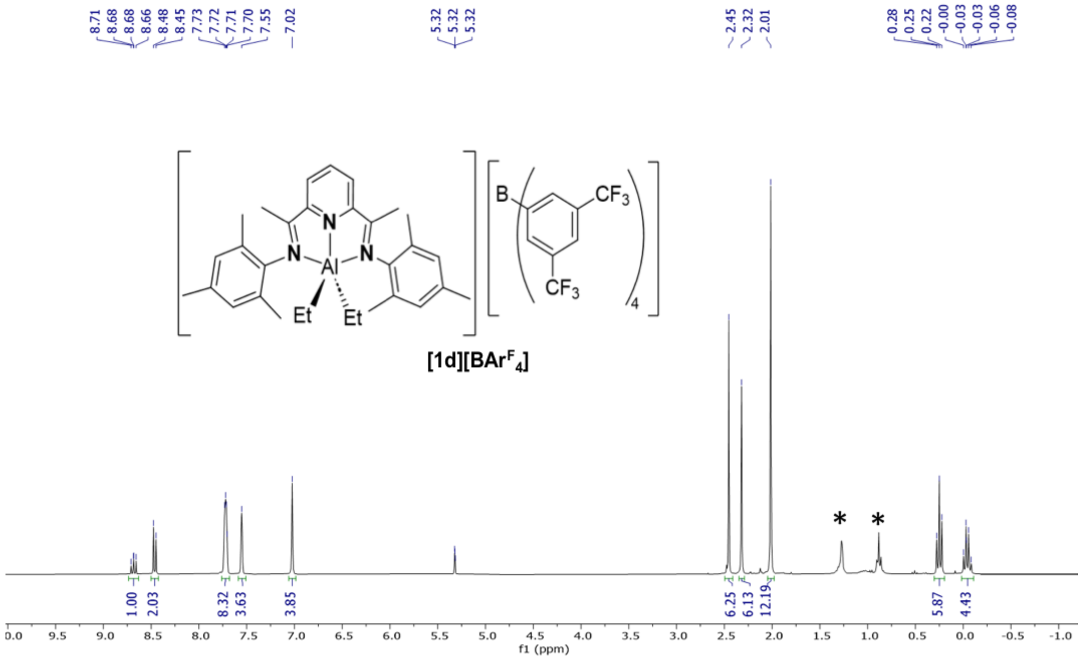

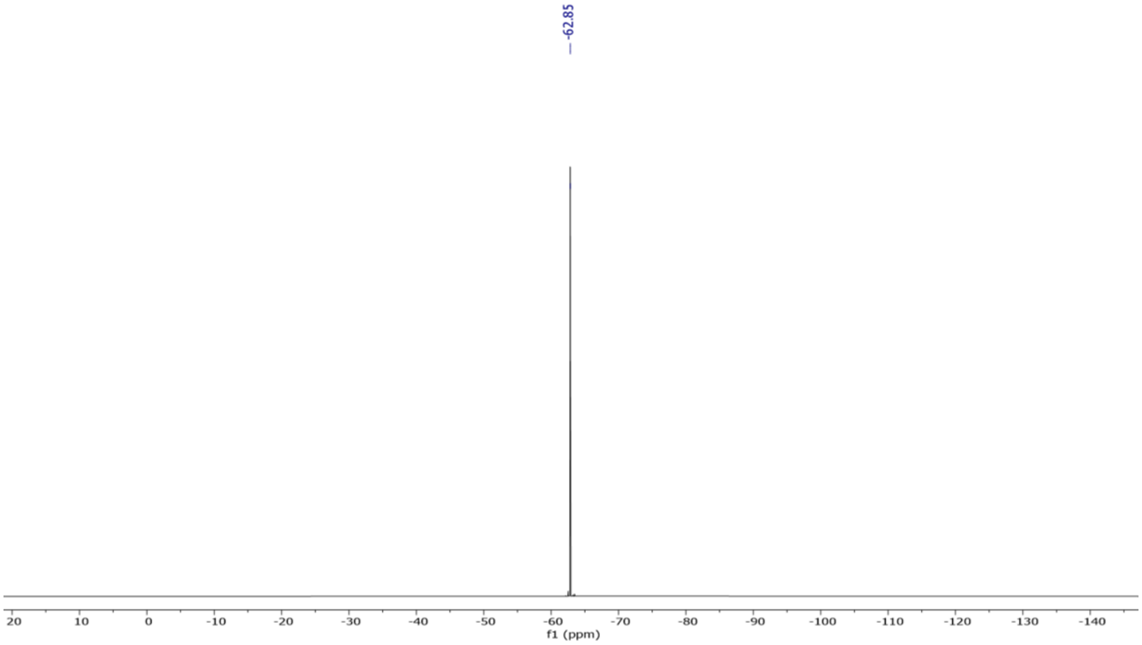

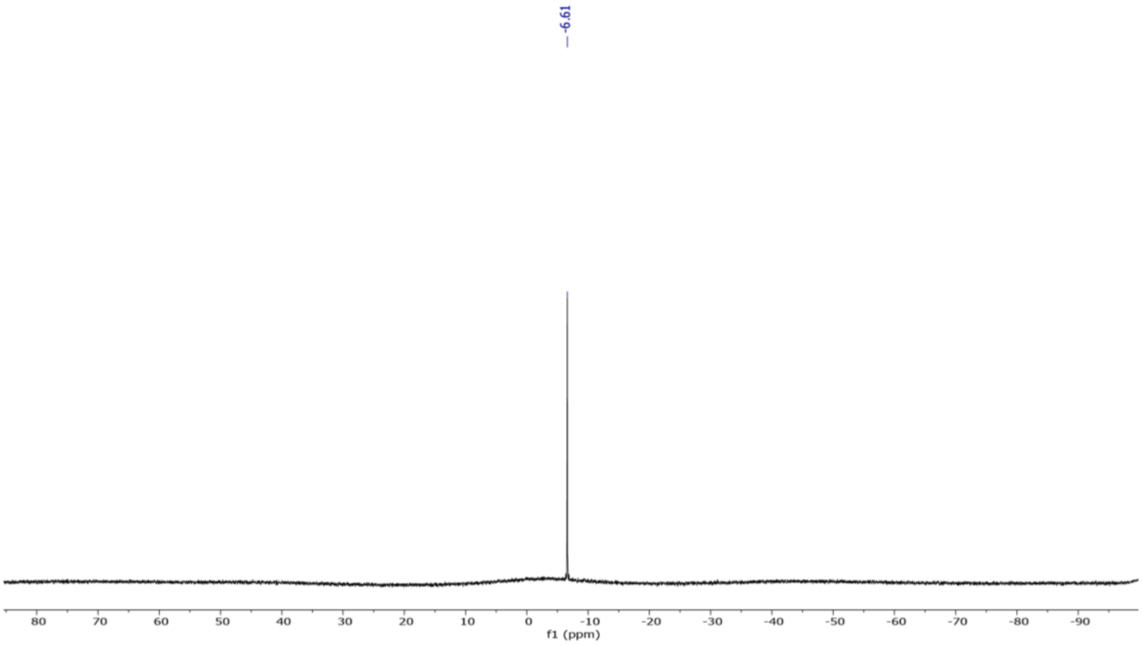

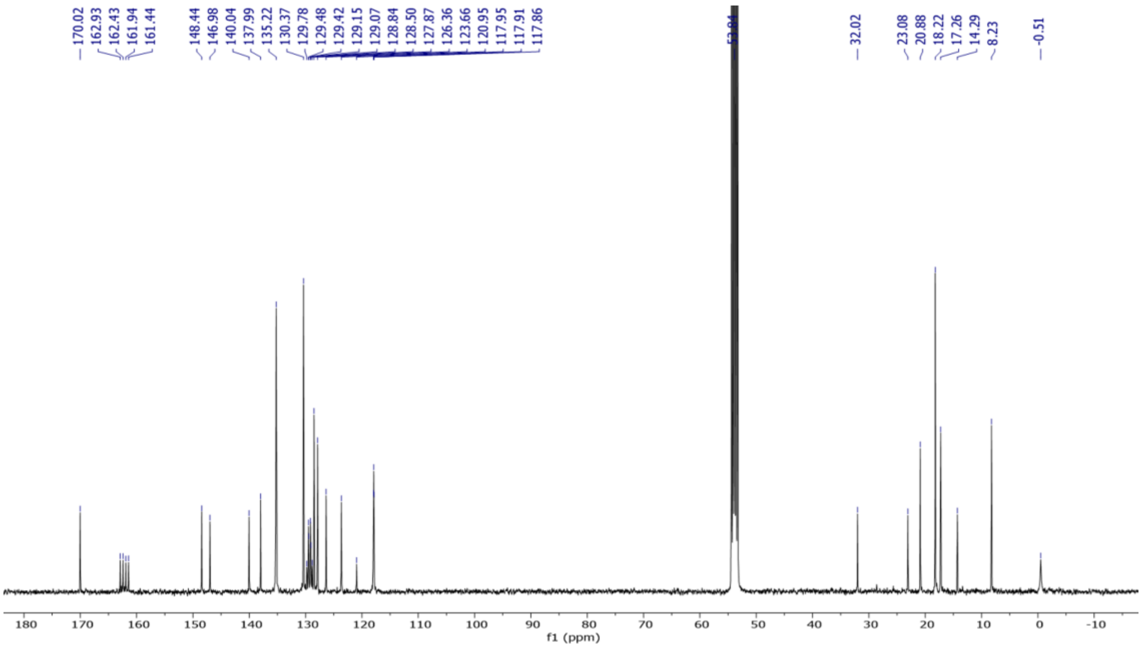

Figure S12. **^{1}H-** (CD_2Cl_2, 25 °C, 400 MHz), **^{19}F{^{1}H}-** (376 MHz), **^{11}B{^{1}H}-** (128 MHz) and **^{13}C{^{1}H}-NMR** (100 MHz) spectra of **([1d][BAr$^{F}_{4}$]).** Resonances marked with asterisks in the ^{1}H-NMR were assigned for retraces of some of the solvents (hexane) used in the crystallization process.

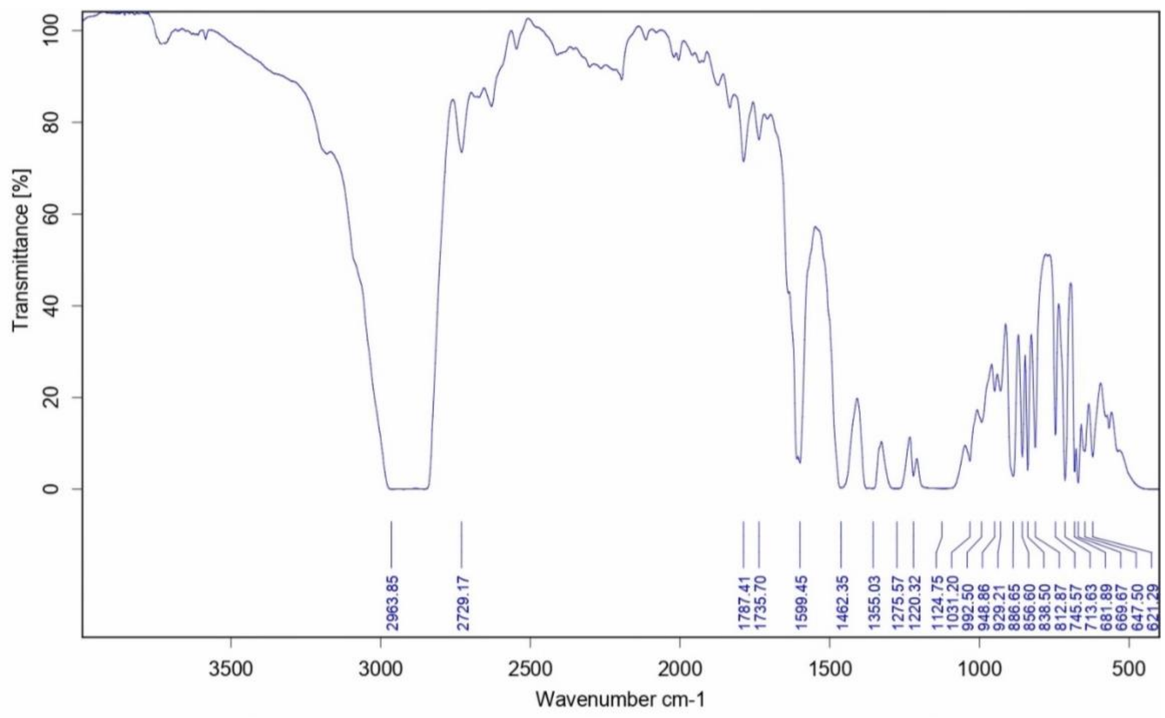

Figure S13. IR (KBr/Nujol, cm^{-1}) spectrum of **([1d][BAr$^{F}{}_{4}$])**.

(^{Mes}BIP·)AlEt$_2$ (2d)

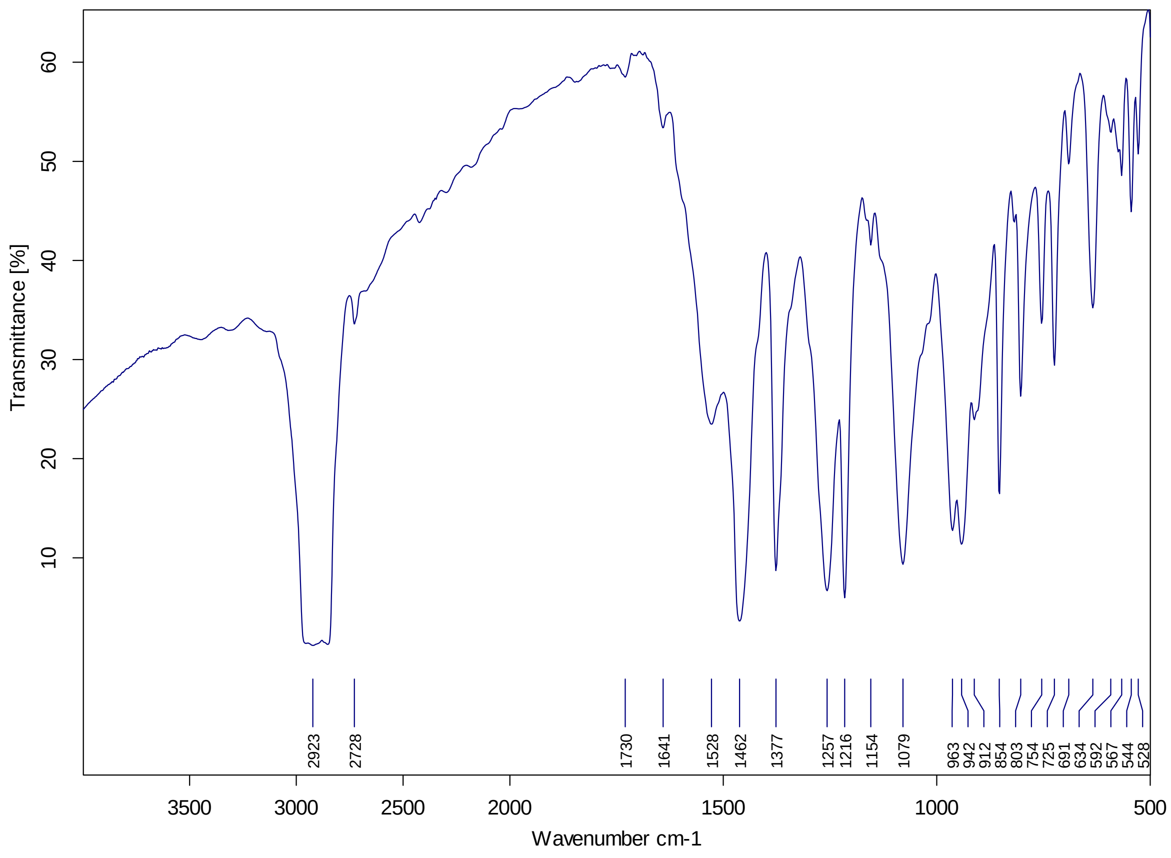

Figure S14. **IR (KBr/Nujol, cm^{-1})** spectrum of **(^{Mes}BIP·)AlEt$_2$ (2d)**

^{1}H NMR redox monitoring for $[Al(Me)_2(^{DiPP}BIP)][BAr^F{}_4]$ ⇄ $(^{DiPP}BIP\cdot)AlMe_2$ ($[\mathbf{1a}][BAr^F{}_4]$ ⇄ **2a**)

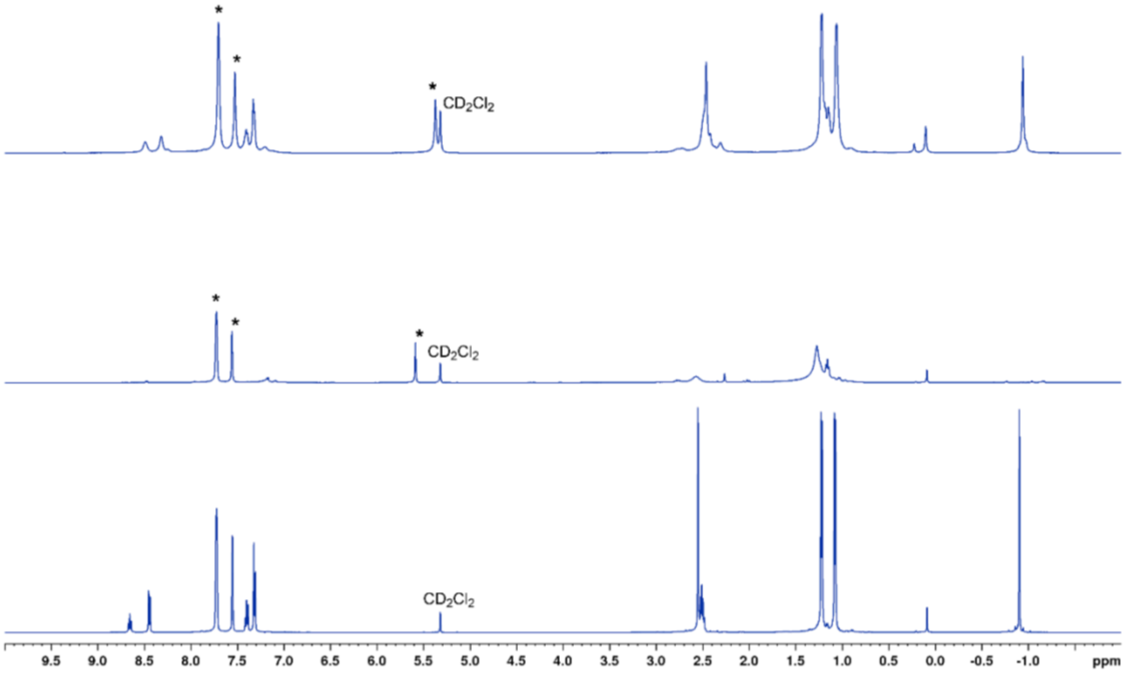

Figure S15. ^{1}H NMR spectroscopy (500 MHz, CD_2Cl_2, 298 K) study for the in-situ reduction of **($[\mathbf{1a}][BAr^F{}_4]$)** (bottom) with Cp_2Co to give **2a** (middle) and consecutively oxidation with $[Fc][PF_6]$ to give $[\mathbf{1a}][\mathbf{PF_6}]$ (top). Resonances marked with * correspond to $[Cp_2Co][BAr^F{}_4]$ and Fc species.

Notes on the ^{1}H NMR spectroscopic study of $[\mathbf{1a}][BAr^F{}_4]$ ⇄ 2a in CD_2Cl_2 solution (298 K):

In a CD_2Cl_2 solution at 298 K, the redox cycle was clearly demonstrated. The initial reaction of $[\mathbf{1a}][\mathbf{BAr^F{}_4}]$ with Cp_2Co resulted in the complete disappearance of signals, indicating the formation of the paramagnetic species **2a**. Upon oxidation with $[Fc][PF_6]$ these signals reappeared, confirming the reformation of the diamagnetic species $[\mathbf{1a}][\mathbf{PF_6}]$.The presence of broadened signals in the top spectrum can be attributed to trace amounts of paramagnetic species, such as **2a**, $[Fc]^+$, or $[Cp_2Co]$, which are remnants from the in-situ experimental conditions.

^{1}H NMR redox monitoring for [Al(Me)$_2$(^{Mes}BIP)][BAr^F$_4$] ⇄ Al(Me)$_2$(^{Mes}BIP·) ([1b][BAr^F$_4$] ⇄ 2b)

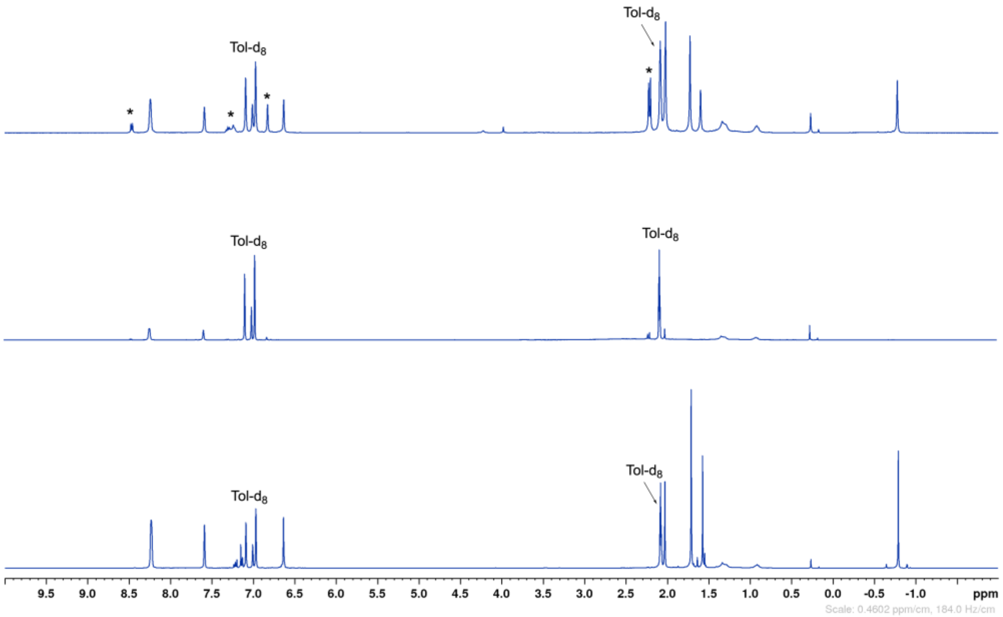

Figure S16. ^{1}H NMR spectroscopy (400 MHz, Tol-d_8, 298 K) study for the in-situ reduction of **[1b][BAr^F$_4$]** (bottom) with Cp$_2$Co to give **2b** (middle) and consecutively oxidation with [Fc][PF$_6$] to give [**2b**][**PF$_6$**] (top). Resonances marked with * correspond to unavoidable trace amounts of hydrolysis to give free BIP ligand.

Notes on the ^{1}H NMR spectroscopic study of [1b][BAr^F$_4$] ⇄ 2b in Tol-d_8 solution (298 K):

In a Tol-d_8 solution at 298 K, the redox cycle was clearly demonstrated. The initial reaction of [**1b**][**BAr^F$_4$**] with Cp$_2$Co resulted in the complete disappearance of signals, indicating the formation of the paramagnetic species **2b**. Upon oxidation with [Fc][PF$_6$] these signals reappeared, confirming the reformation of the diamagnetic species [**1b**][**PF$_6$**] he presence of broadened signals in the top spectrum can be attributed to trace amounts of paramagnetic species, such as **2b**, [Fc]$^+$, or [Cp$_2$Co], which are remnants from the in-situ experimental conditions.

^{1}H NMR redox monitoring for [Al(Et)$_2$(^{DiPP}BIP)][BAr$^F_4$] ⇄ Al(Et)$_2$(^{DiPP}BIP·) ([1c][BAr$^F_4$] ⇄ 2c)

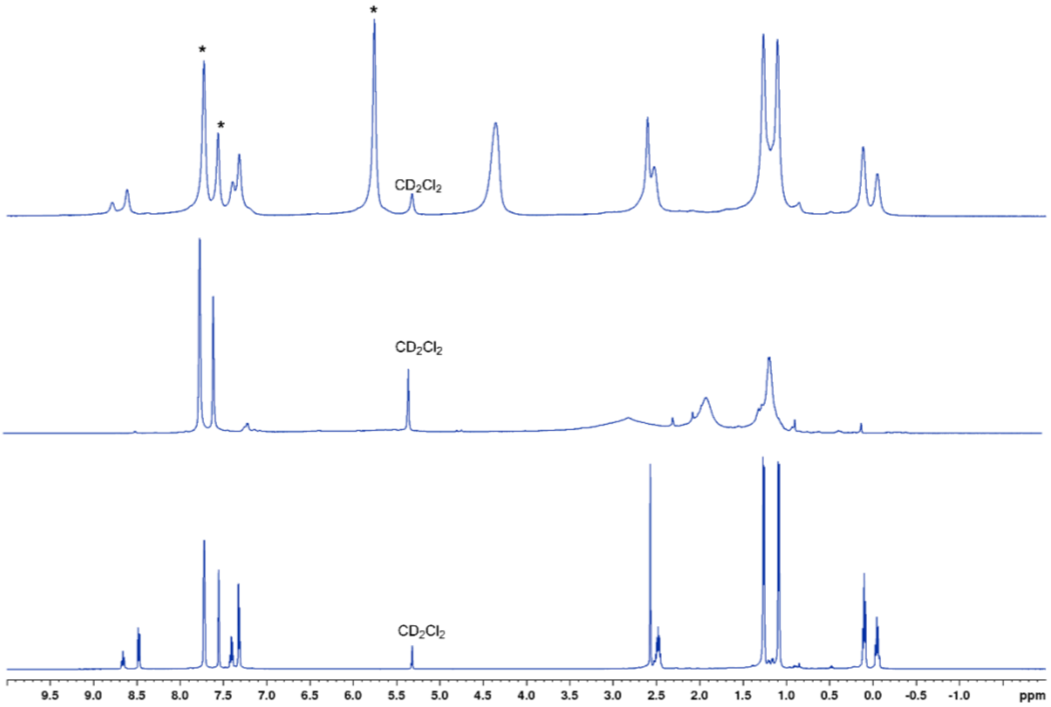

Figure S17. ^{1}H NMR spectroscopy (500 MHz, CD_2Cl_2, 298 K) study for the in-situ reduction of **[1c][BAr$^F_4$]** (bottom) with Cp_2Co to give **2c** (middle) and consecutively oxidation with [Fc][PF_6] to give **[1c][PF$_6$]** (top). Resonances marked with * correspond to [Cp_2Co][BAr$^F_4$] and Fc species.

Notes on the ^{1}H NMR spectroscopic study of [1c][BAr$^F_4$] ⇄ 2c in CD_2Cl_2 solution (298 K):

In a CD_2Cl_2 solution at 298 K, the redox cycle was clearly demonstrated. The initial reaction of **[1c][BAr$^F_4$]** with Cp_2Co resulted in the complete disappearance of signals, indicating the formation of the paramagnetic species **2c.** Upon oxidation with [Fc][PF_6] these signals reappeared, confirming the reformation of the diamagnetic species **[1c][PF$_6$]**. The presence of broadened signals in the top spectrum can be attributed to trace amounts of paramagnetic species, such as **2c**, [Fc]$^+$, or [Cp_2Co], which are remnants from the in-situ experimental conditions.

^{1}H NMR redox monitoring for [Al(Et)$_2$(^{Mes}BIP)][BAr^F$_4$] ⇄ Al(Et)$_2$(^{Mes}BIP·) ([1d][BAr^F$_4$] ⇄ 2d)

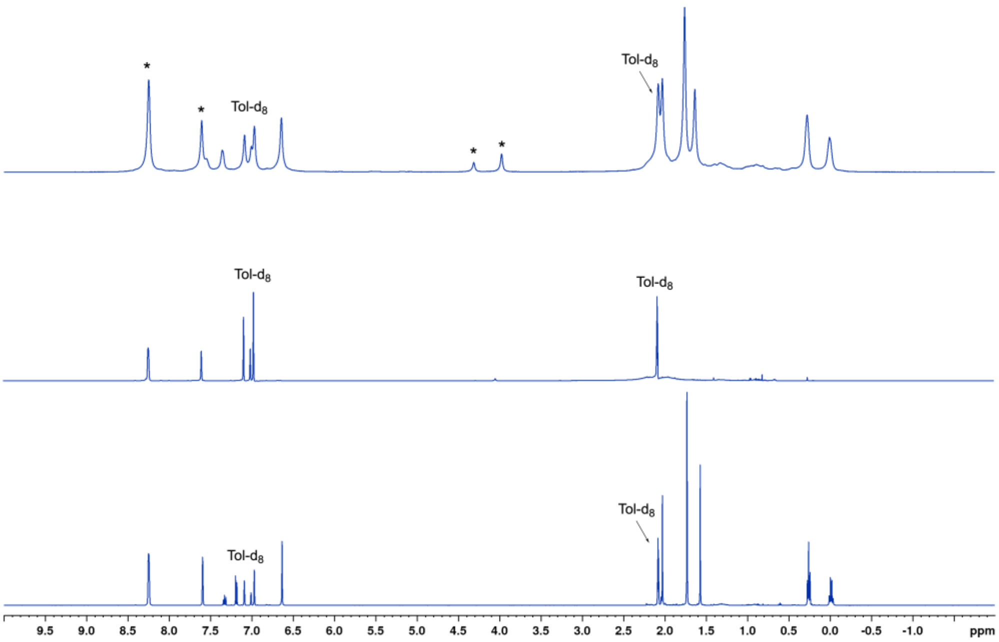

Figure S18. ^{1}H NMR spectroscopy (400 MHz, Tol-d_8, 298 K) study for the in-situ reduction of **[1d][BAr^F$_4$]** (bottom) with Cp_2Co to give **2d** (middle) and consecutively oxidation with [Fc][PF_6] to give **[1d][PF$_6$]** (top). Resonances marked with * correspond to [Cp_2Co][BAr^F$_4$] and Fc species.

Notes on the ^{1}H NMR spectroscopic study of ([1d][BAr^F$_4$]⇄ 2d) in Tol-d_8 solution (298 K):

In a Tol-d_8 solution at 298 K, the redox cycle was clearly demonstrated. The initial reaction of **[1d][BAr^F$_4$]** with Cp_2Co resulted in the complete disappearance of signals, indicating the formation of the paramagnetic species **2d** Upon oxidation with [Fc][PF_6] these signals reappeared, confirming the reformation of the diamagnetic species **[2d][PF$_6$].** The presence of broadened signals in the top spectrum can be attributed to trace amounts of paramagnetic species, such as **2d**, [Fc]$^+$, or [Cp_2Co], which are remnants from the in-situ experimental conditions.

Cyclic voltammetry (CV) of compounds $1a^{+}$-d^{+} and $[H^{DiPP}BIP][BAr^{F}_4]$

CV of compound $[1a][BAr^{F}_4]$

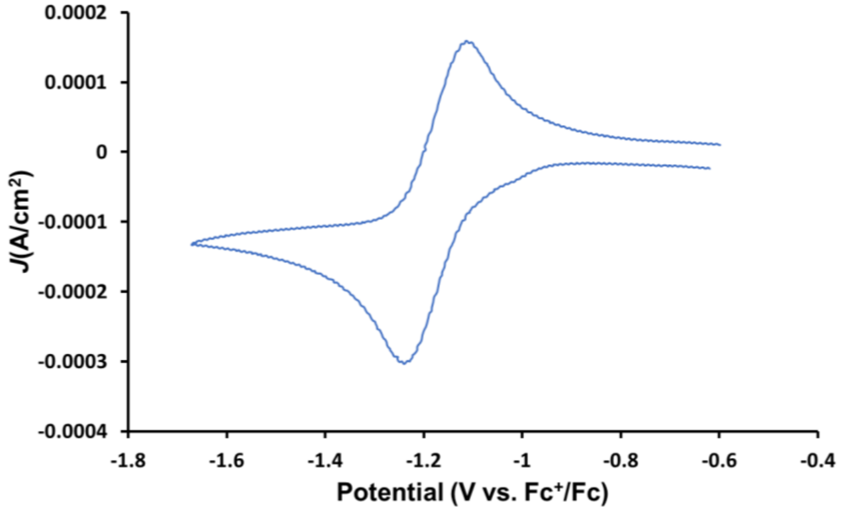

Figure S19. CV of complex $[1a][BAr^{F}_4]$ under N_2 saturation conditions, showing a reversible reduction feature at E = −1.18 V vs Fc^{+}/Fc. Conditions: 1.0 mM analyte, 0.1 M $TBAPF_6$/dichloromethane; glassy carbon working electrode, glassy carbon counter electrode, Ag/AgCl pseudoreference electrode; 100 mV/s scan rate; referenced to internal ferrocene standard.

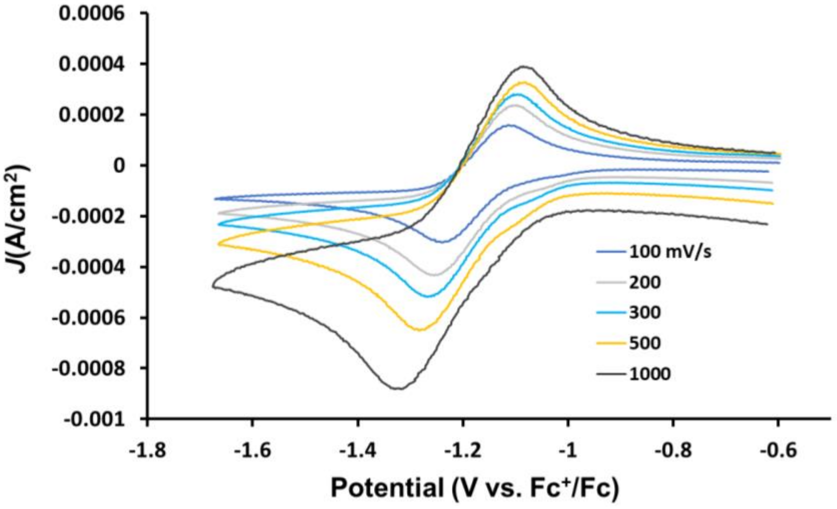

Figure S20. CVs of $[1a][BAr^{F}_4]$ at variable scan rates ranging from 100 to 1000 mV/s, obtained under N_2 saturation conditions.

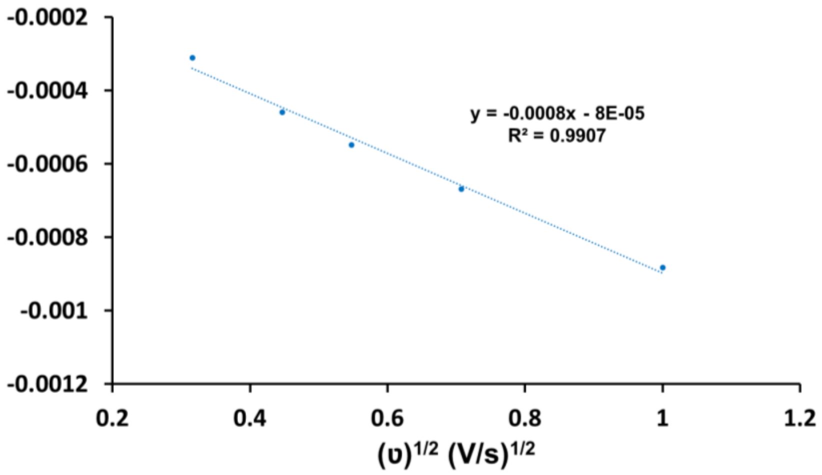

Figure S21. Linear fit of variable scan rate data from Figure S20, demonstrating that **[1a][BAr$^F{}_4$]** shows a diffusion-limited current response.

CV of compound [1b][BAr$^F{}_4$]

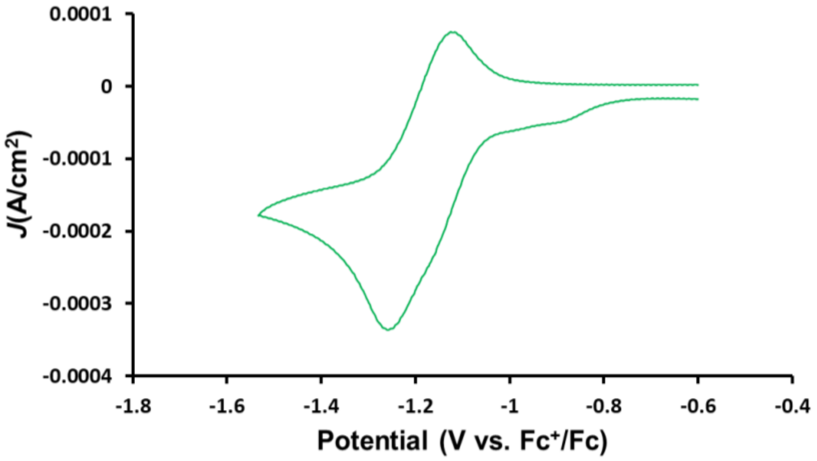

Figure S22. CV of complex [**1b][BAr$^F{}_4$]** under N_2 saturation conditions, showing a reversible reduction feature at E = −1.19 V vs Fc^+/Fc. Conditions: 1.0 mM analyte, 0.1 M $TBAPF_6$/dichloromethane; glassy carbon working electrode, glassy carbon counter electrode, Ag/AgCl pseudoreference electrode; 100 mV/s scan rate; referenced to internal ferrocene standard.

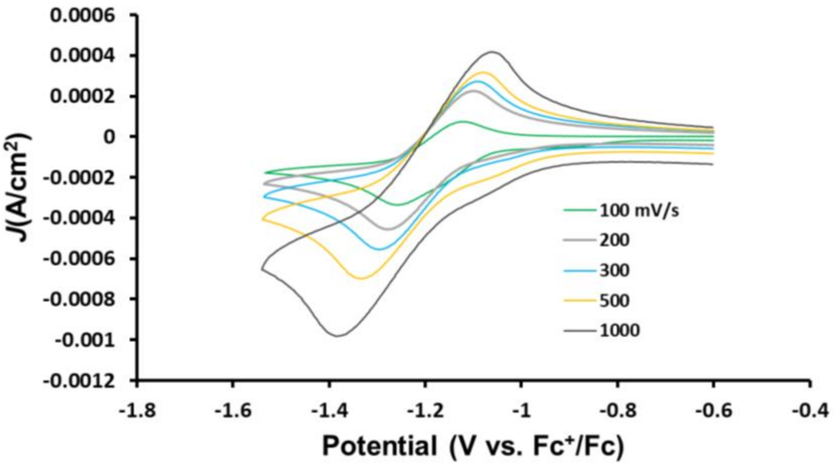

Figure S23. CVs of **[1b][BAr$^F{}_4$]** at variable scan rates ranging from 100 to 1000 mV/s, obtained under N_2 saturation conditions.

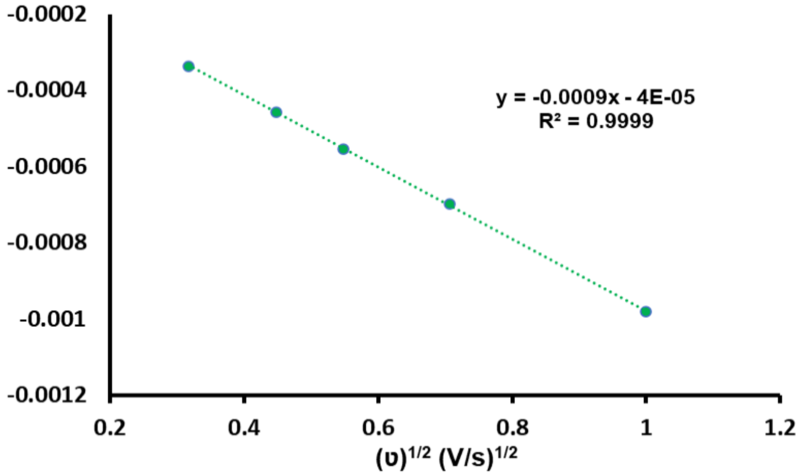

Figure S24. Linear fit of variable scan rate data from Figure S23 demonstrating that **[1b][BAr$^F{}_4$]** shows a diffusion-limited current response.

CV of compound [1c][BAr$^F_4$]

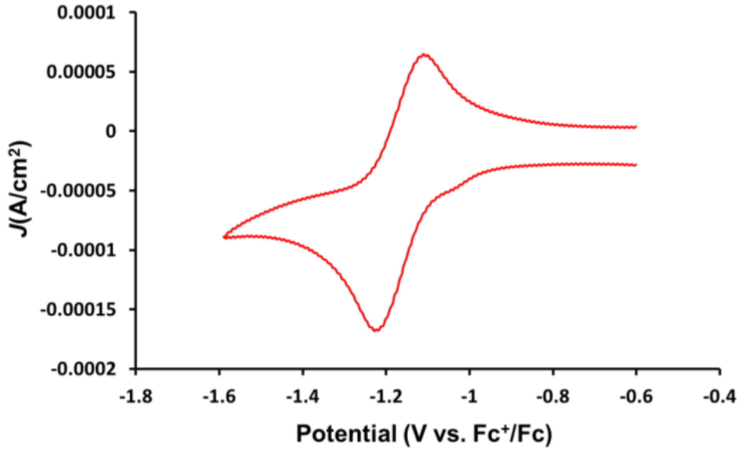

Figure S25. CV of complex **[1c][BAr$^F_4$]** under N_2 saturation conditions, showing a reversible reduction feature at E = −1.17 V vs Fc^+/Fc. Conditions: 1.0 mM analyte, 0.1 M $TBAPF_6$/dichloromethane; glassy carbon working electrode, glassy carbon counter electrode, Ag/AgCl pseudoreference electrode; 100 mV/s scan rate; referenced to internal ferrocene standard.

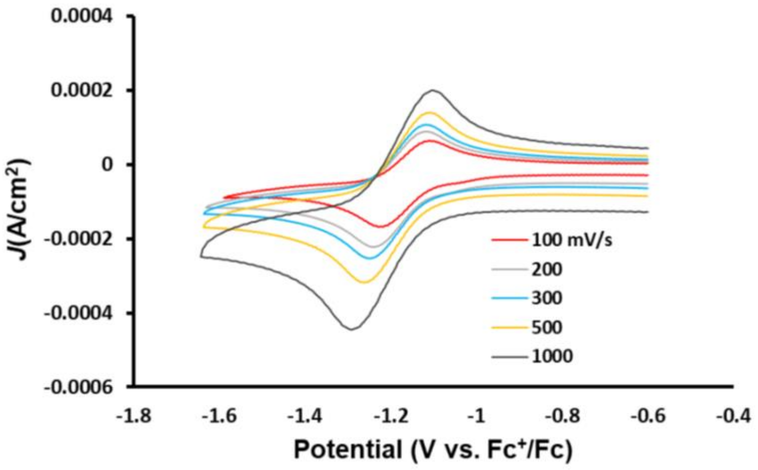

Figure S26. CVs of **[1c][BAr$^F_4$]** at variable scan rates ranging from 100 to 1000 mV/s, obtained under N_2 saturation conditions.

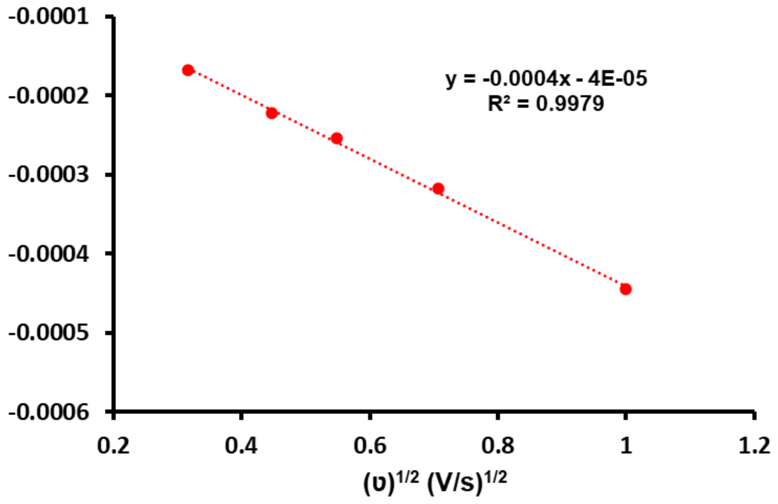

Figure S27. Linear fit of variable scan rate data from Figure S25, demonstrating that **[1c][BAr$^{F}{}_{4}$]** shows a diffusion-limited current response.

CV of compound [1d][BAr$^{F}{}_{4}$]

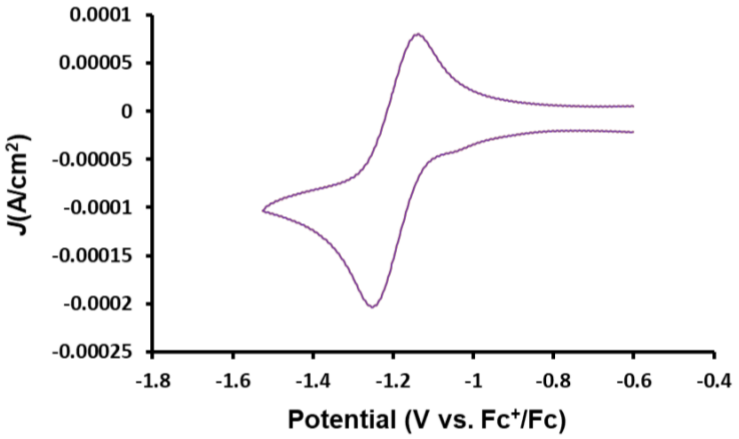

Figure S28. CV of complex **[1d][BAr$^{F}{}_{4}$]** under N_2 saturation conditions, showing a reversible reduction feature at E = −1.19 V vs Fc^{+}/Fc. Conditions: 1.0 mM analyte, 0.1 M $TBAPF_6$/dichloromethane; glassy carbon working electrode, glassy carbon counter electrode, Ag/AgCl pseudoreference electrode; 100 mV/s scan rate; referenced to internal ferrocene standard.

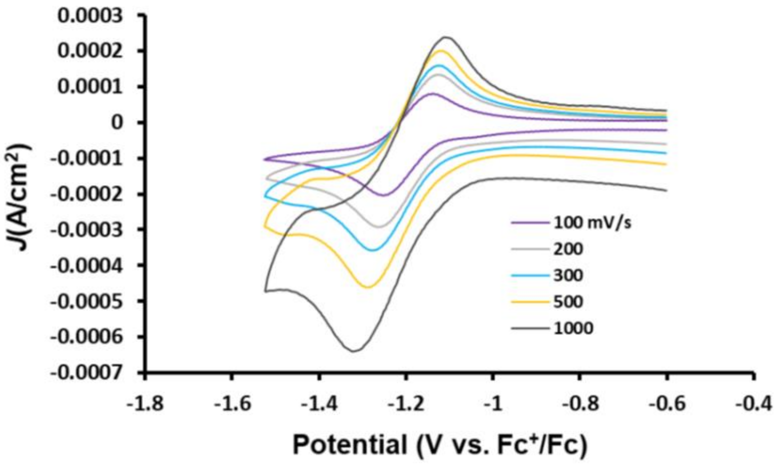

Figure S29. CVs of **[1d][BAr$^{F}_{4}$]** at variable scan rates ranging from 100 to 1000 mV/s, obtained under N_2 saturation conditions.

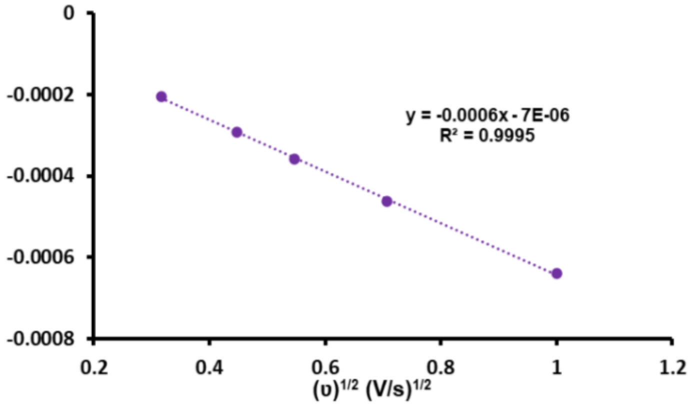

Figure S30. Linear fit of variable scan rate data from Figure S28, demonstrating that **[1d][BAr$^{F}_{4}$]** shows a diffusion-limited current response.

CV of [H^{DiPP}BIP][BAr$^F_4$]

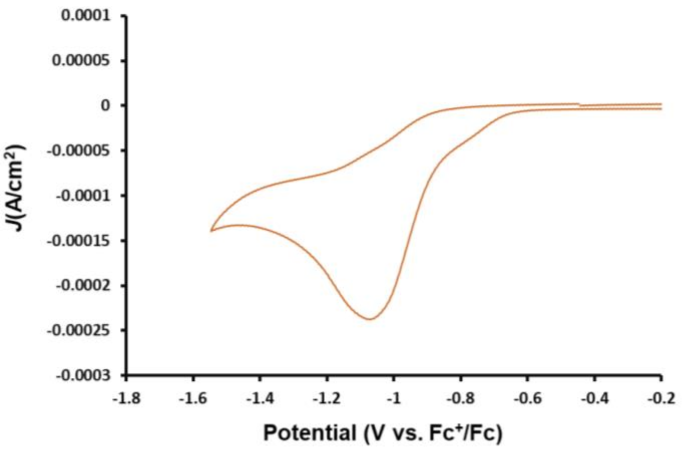

Figure S31. CV of system **[H^{DiPP}BIP][BAr$^F_4$]** under N_2 saturation conditions, showing an irreversible reduction feature at $E_p = -1.07$ V vs Fc^+/Fc. Conditions: 1.0 mM analyte, 0.1 M $TBAPF_6$/dichloromethane; glassy carbon working electrode, glassy carbon counter electrode, Ag/AgCl pseudoreference electrode; 100 mV/s scan rate; referenced to internal ferrocene standard.

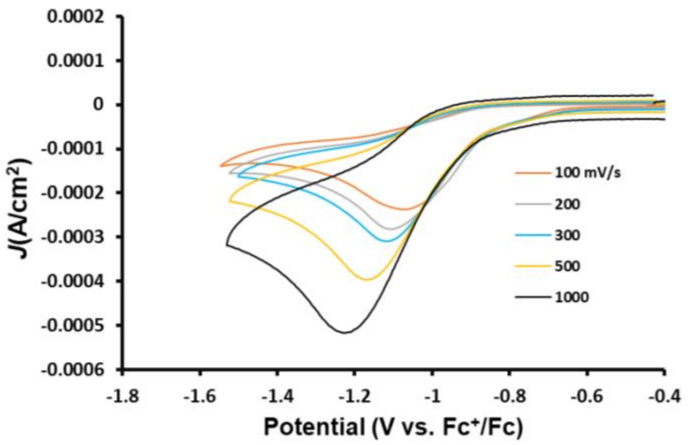

Figure S32. CVs of **[H^{DiPP}BIP][BAr$^F_4$]** at variable scan rates ranging from 100 to 1000 mV/s, obtained under N_2 saturation conditions.

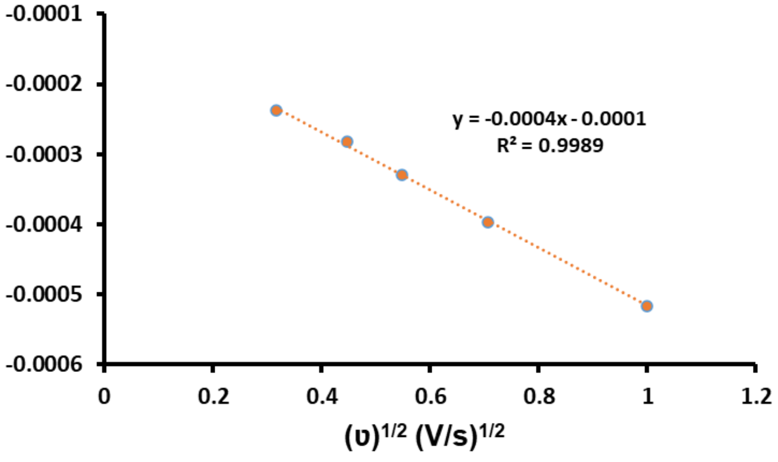

Figure S33. Linear fit of variable scan rate data from Figure S31, demonstrating that **[H^{DiPP}BIP][BAr$^F_4$]** shows a diffusion-limited current response.

EPR of complexes 2a-d

Figures S34 to S38 reproduce the experimental and simulated of complexes **2a-d**, and complete listings of the fitted and DFT-computed EPR parameters is given in **Table S10**.

EPR spectra of 2a

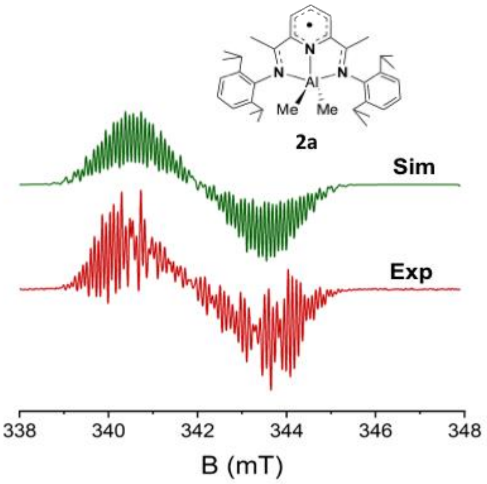

Figure S34. Solution X-band EPR spectrum of **2a** at 293 K. Conditions: Frequency = 9.6054 GHz, modulation amplitude = 0.03 mT, microwave power = 1.000 mW.

EPR spectra of 2b

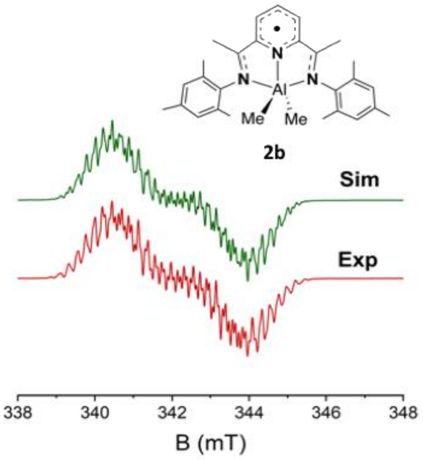

Figure S35. Solution X-band EPR spectrum of **2b** at 293 K. Conditions: Frequency = 9.6027 GHz, modulation amplitude = 0.03 mT, microwave power = 3.981 mW.

EPR spectra of 2c

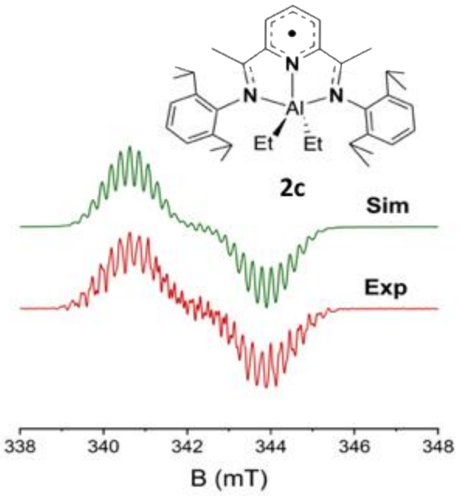

Figure S36. Solution X-band EPR spectrum of **2c** at 293 K. Conditions: Frequency = 9.6008 GHz, modulation amplitude = 0.05 mT, microwave power = 1.995 mW.

EPR spectra of 2d

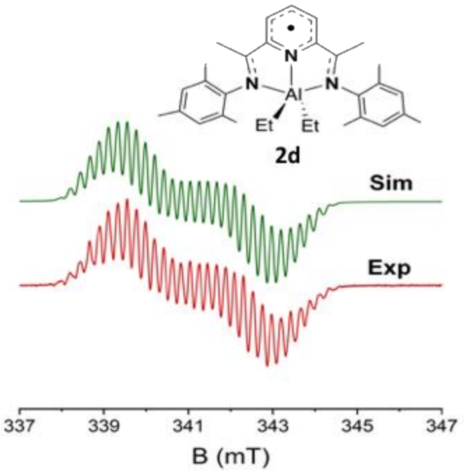

Figure S37. Solution X-band EPR spectrum of **2d** at 293 K. Conditions: Frequency = 9.6019 GHz, modulation amplitude = 0.01 mT, microwave power = 1.995 mW.

Single-Crystal X-ray Analysis

[1a][BAr$^F_4$]

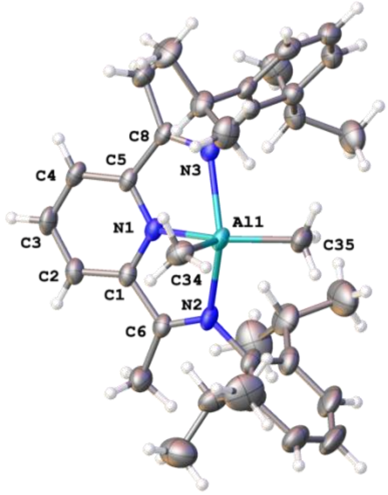

Figure S38. ORTEP view of cationic moiety of complex salt **[1a][BAr$^F_4$]** with thermal ellipsoids depicted at the 50% probability level. [**BAr$^F_4$**] counteranion has been omitted for clarity. Selected bond lengths (Å) and angles (°): Al(1)-N(1) 2.011(4), Al(1)-N(2) 2.149(4), Al(1)-N(3) 2.167(4), Al(1)-C(35) 1.945(5), Al(1)-C(34) 1.959(5), N(1)-C(1) 1.337(6), N(1)-C(5) 1.343(6), N(2)-C(6) 1.288(6), N(2)-C(10) 1.449(6), N(3)-C(8) 1.276(6), N(3)-C(22) 1.468(6), C(1)-C(2) 1.380(6), C(1)-C(6) 1.483(6), C(2)-C(3) 1.370(7), C(3)-C(4) 1.383(7), C(4)-C(5) 1.368(6), C(5)-C(8) 1.498(7), C(6)-C(7) 1.491(7); C(35)-Al(1)-C(34) 120.4(3), C(35)-Al(1)-N(1) 142.5(2), C(34)-Al(1)-N(1) 97.1(2), C(34)-Al(1)-N(2) 99.9(2), C(35)-Al(1)-N(2) 96.5(2), N(1)-Al(1)-N(2) 74.49(15), N(1)-Al(1)-N(3) 74.58(15), C(35)-Al(1)-N(3) 97.2(2), C(34)-Al(1)-N(3) 101.2(2), N(2)-Al(1)-N(3) 144.32(15), C(1)-N(1)-C(5) 120.1(4).

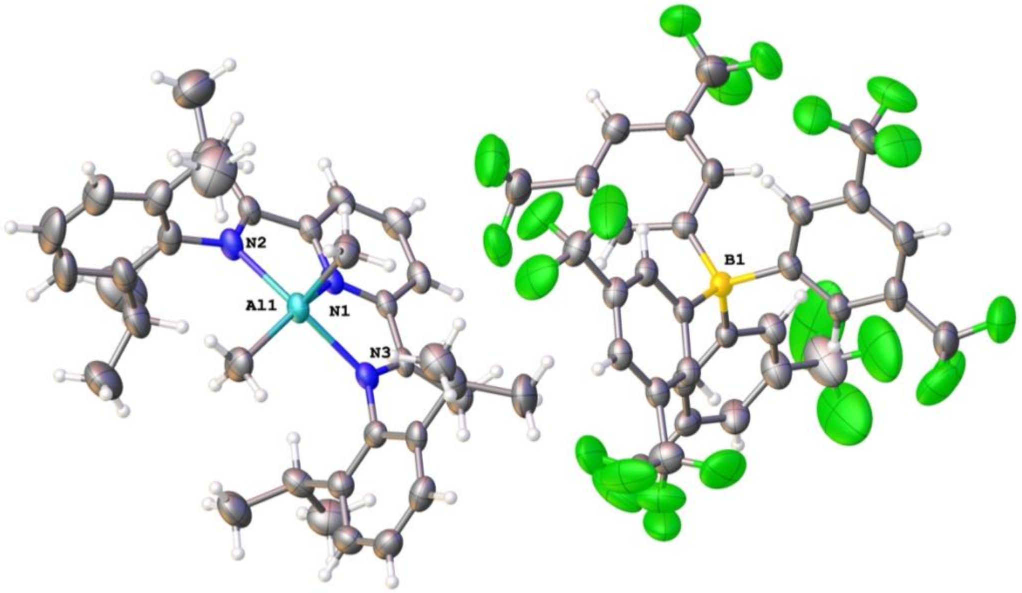

Figure S39. ORTEP view of molecular structure of complex salt **[1a][BAr^F$_4$]**with thermal ellipsoids depicted at the 50% probability level. One of the two components of the disorder models involving four -CF_3 groups of the **[BAr^F$_4$]** anion has been omitted for clarity.

Table S1. Crystal data and structure refinement for **[1a][BAr^F$_4$]**

Empirical formula	$C_{67}H_{61}AlBF_{24}N_3$	
	[$C_{32}H_{12}BF_{24}$, $C_{35}H_{49}AlN_3$]	
Formula weight	1401.97	
Temperature	193(2) K	
Wavelength	0.71073 Å	
Crystal system	Monoclinic	
Space group	$P2_1/c$	
Unit cell dimensions	a = 17.6492(16) Å	α= 90°.
	b = 17.8609(16) Å	β= 105.564(3)°.
	c = 22.870(2) Å	γ = 90°.
Volume	6944.9(11) Å^3	
Z	4	
Density (calculated)	1.341 Mg/m^3	
Absorption coefficient	0.135 mm^{-1}	
F(000)	2872	
Crystal size	0.450 x 0.300 x 0.150 mm^3	
Theta range for data collection	2.045 to 25.099°.	
Index ranges	-21<=h<=21, -21<=k<=21, -27<=l<=27	
Reflections collected	124495	

Independent reflections	12361 [R(int) = 0.1325]
Completeness to theta = 25.099°	99.9 %
Absorption correction	Semi-empirical from equivalents
Max. and min. transmission	0.7461 and 0.5790
Refinement method	Full-matrix least-squares on F2
Data / restraints / parameters	12361 / 519 / 1017
Goodness-of-fit on F2	1.092
Final R indices [I>2sigma(I)]	R1 = 0.0861, wR2 = 0.2272
R indices (all data)	R1 = 0.1388, wR2 = 0.2509
Extinction coefficient	n/a
Largest diff. peak and hole	0.81 and -0.55 e.Å-3

[1c][PF$_6$]

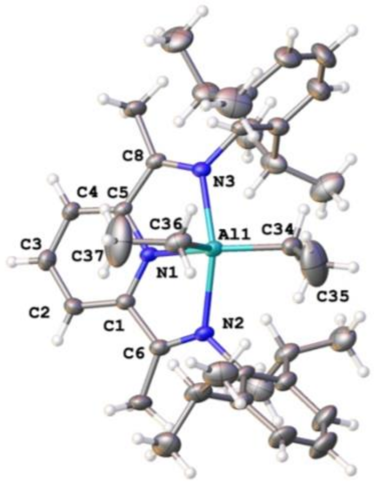

Figure S40. ORTEP view of cationic moiety of complex salt **[1c][PF$_6$]** with thermal ellipsoids depicted at the 50% probability level. **[PF$_6$]** counteranion has been omitted for clarity. Selected bond lengths (Å) and angles (°): Al(1)-N(1) 1.997(3), Al(1)-N(2) 2.175(3), Al(1)-N(3) 2.164(3), Al(1)-C(34) 1.969(4), Al(1)-C(36) 1.974(5), N(1)-C(1) 1.340(4), N(1)-C(5) 1.345(4), N(2)-C(6) 1.277(5), N(2)-C(10) 1.452(4), N(3)-C(8) 1.285(4), N(3)-C(22) 1.455(4), C(1)-C(2) 1.386(5), C(1)-C(6) 1.490(5), C(2)-C(3) 1.386(5), C(3)-C(4) 1.377(5), C(4)-C(5) 1.379(5), C(5)-C(8) 1.476(5), C(6)-C(7) 1.491(5) ; C(34)-Al(1)-C(36)114.8(2), C(34)-Al(1)-N(1) 145.61(16), C(36)-Al(1)-N(1) 99.57(17), C(34)-Al(1)-N(2) 98.01(15), C(36)-Al(1)-N(2) 101.55(17), N(1)-Al(1)-N(2) 74.78(11), N(1)-Al(1)-N(3) 74.72(11), C(34)-Al(1)-N(3) 96.93(15), C(36)-Al(1)-N(3) 101.01(16), N(2)-Al(1)-N(3) 144.51(12), C(1)-N(1)-C(5) 120.0(3).

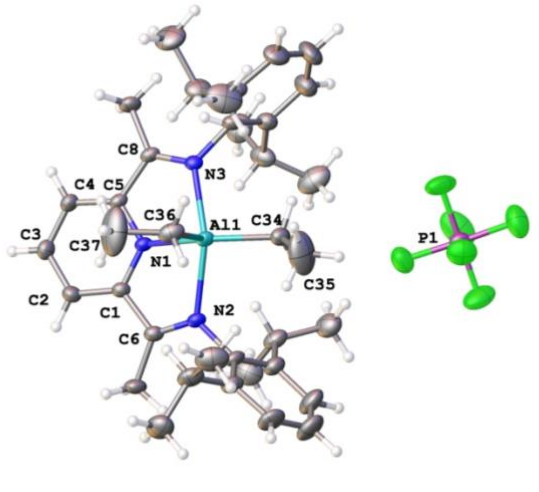

Figure S41. ORTEP view of molecular structure of complex salt **[1c][PF$_6$]** with thermal ellipsoids depicted at the 50% probability level.

Table S2. Crystal data and structure refinement for **[1c][PF$_6$]**

Empirical formula	$C_{41}H_{61}AlF_6N_3P$
Formula weight	783.87
Temperature	193(2) K

Wavelength	0.71073 Å	
Crystal system	Triclinic	
Space group	P$\bar{1}$	
Unit cell dimensions	a = 12.3360(12) Å	α= 67.741(4)°.
	b = 13.1456(12) Å	β= 70.736(4)°.
	c = 16.4311(19) Å	γ = 80.221(4)°.
Volume	2324.8(4) Å^3	
Z	2	
Density (calculated)	1.120 Mg/m^3	
Absorption coefficient	0.133 mm^{-1}	
F(000)	836	
Crystal size	0.100 x 0.050 x 0.030 mm^3	
Theta range for data collection	1.897 to 25.248°.	
Index ranges	-14<=h<=14, -15<=k<=15, -19<=l<=19	
Reflections collected	73572	
Independent reflections	8414 [R(int) = 0.1168]	
Completeness to theta = 25.242°	100.0 %	
Absorption correction	Semi-empirical from equivalents	
Max. and min. transmission	0.7461 and 0.6481	
Refinement method	Full-matrix least-squares on F^2	
Data / restraints / parameters	8414 / 112 / 490	
Goodness-of-fit on F2	1.073	
Final R indices [I>2sigma(I)]	R1 = 0.0846, wR2 = 0.2298	
R indices (all data)	R1 = 0.1157, wR2 = 0.2476	
Extinction coefficient	n/a	
Largest diff. peak and hole	1.004 and -0.869 e.Å^{-3}	

[1d][BAr$^{F}_{4}$]

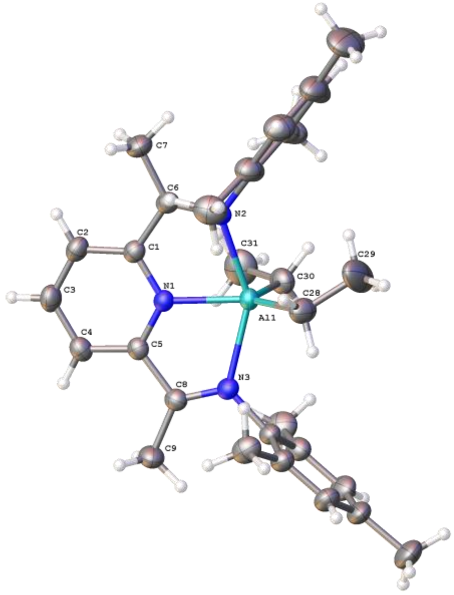

Figure S42. ORTEP view of molecular structure of complex salt **[1d][BAr$^{F}_{4}$]** with thermal ellipsoids depicted at the 50% probability level. Selected bond lengths (Å) and angles (°): Al(1)-N(1) 2.019(2), Al(1)-N(2) 2.177(2), Al(1)-N(3) 2.191(2), Al(1)-C(28) 1.968(3), Al(1)-C(30) 1.974(3), N(1)-C(1) 1.343(3), N(1)-C(5) 1.344(3), N(2)-C(6) 1.288(3), N(2)-C(10) 1.455(3), N(3)-C(8) 1.280(3), N(3)-C(19) 1.450(3), C(1)-C(2) 1.391(3), C(1)-C(6) 1.481(3), C(2)-C(3) 1.383(4), C(3)-C(4) 1.383(4), C(4)-C(5) 1.395(3), C(5)-C(8) 1.487(3), C(6)-C(7) 1.495(3); C(28)-Al(1)-C(30) 117.27(13), C(28)-Al(1)-N(1) 137.18(12), C(30)-Al(1)-N(1) 105.55(10), C(28)-Al(1)-N(2) 97.10(11), C(30)-Al(1)-N(2) 99.54(10), N(1)-Al(1)-N(2) 75.20(8), N(1)-Al(1)-N(3) 74.45(8), C(28)-Al(1)-N(3) 98.73(10), C(30)-Al(1)-N(3) 97.39(10), N(2)-Al(1)-N(3) 148.23(8), C(1)-N(1)-C(5) 119.92(19).

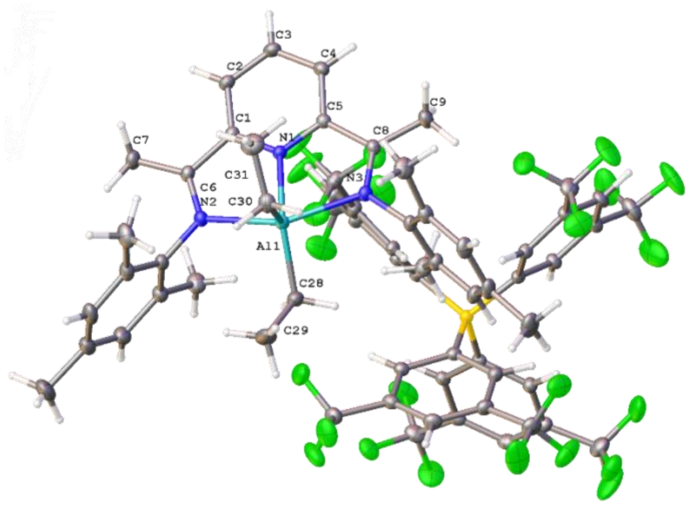

Figure S43. ORTEP view of molecular structure of complex salt **[1d][BAr$^{F}_{4}$]** with thermal ellipsoids depicted at the 50% probability level. One of the two components of the disorder models involving four -CF_3 groups of the **[BAr$^{F}_{4}$]** anion and a molecule of CH_2Cl_2 has been omitted for clarity.

Table S3. Crystal data and structure refinement for **[1d][BAr$^{F}_{4}$]**

Empirical formula	$C_{70}H_{69}AlBCl_2F_{24}N_3$
Formula weight	1516.97

Temperature	193(2) K	
Wavelength	0.71073 Å	
Crystal system	Triclinic	
Space group	$P\bar{1}$	
Unit cell dimensions	a = 11.9464(16) Å	α= 103.232(7)°
	b = 15.586(3) Å	β= 94.019(7)°
	c = 20.816(4) Å	γ = 102.735(6)°
Volume	3650.4(10) Å^3	
Z	2	
Density (calculated)	1.380 Mg/m^3	
Absorption coefficient	0.205 mm^{-1}	
F(000)	1556.0	
Crystal size	0.35 x 0.25 x 0.20 mm^3	
Theta range for data collection	3.916 to 52.712	
Index ranges	$-14 \le h \le 14$, $-19 \le k \le 19$, $-25 \le l \le 25$	
Reflections collected	180379	
Independent reflections	14825 [R_{int} = 0.0692, R_{sigma} = 0.0360]	
Completeness to theta = 25.242°	100.0 %	
Absorption correction	Semi-empirical from equivalents	
Max. and min. transmission	0.7461 and 0.6481	
Refinement method	Full-matrix least-squares on F^2	
Data / restraints / parameters	14825/88/905	
Goodness-of-fit on F^2	1.021	
Final R indexes [I>2sigma(I)]	R_1 = 0.0689, wR_2 = 0.1846	
R indexes (all data)	R_1 = 0.0821, wR_2 = 0.1998	
Extinction coefficient	n/a	
Largest diff. peak and hole	0.94/-1.08 Å^{-3}	

[Cp_2Co][$BAr^F{}_4$]

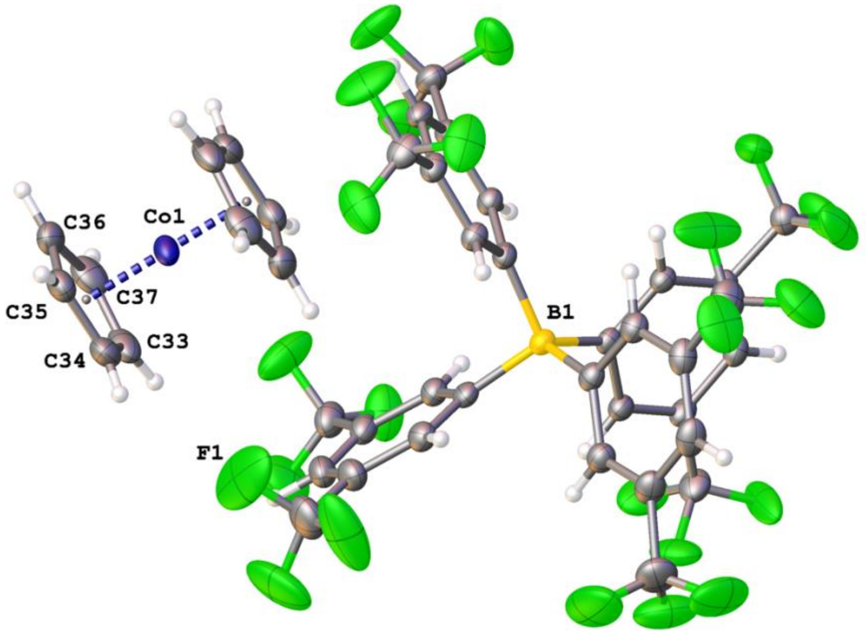

Figure S44 Molecular structure of **[Cp_2Co][$BAr^F{}_4$]** with displacement ellipsoids at 50% probability. Minor disordered components of the CF_3 and Cp groups have been omitted for clarity. Selected bond lengths (Å): Co1···Cent1 1.63, C(33)-C(34) 1.406(5), C(33)-C(37) 1.416(5), C(34)-C(35) 1.404(5), C(35)-C(36) 1.399(5), C(36)-C(37) 1.408(5), C(1)-B(1) 1.635(3), C(9)-B(1) 1.638(3), C(17)-B(1) 1.635(3), C(25)-B(1) 1.632(3). Cent1 = [C33,C34,C35,C36,C37].

Table S4. Crystal data and structure refinement for **[Cp_2Co][$BAr^F{}_4$]**

Empirical formula	$C_{42}H_{22}BCoF_{24}$	
Formula weight	1052.33	
Temperature	150(1) K	
Wavelength	0.71073 Å	
Crystal system	Triclinic	
Space group	P-1	
Unit cell dimensions	a = 12.8766(2) Å	α= 93.3910(10)°
	b = 13.2396(2) Å	β=90.2090(10)°
	c = 14.3298(2) Å	γ = 118.7630(10)°
Volume	2136.35(6) Å^3	
Z	2	
Density (calculated)	1.636 Mg/m^3	
Absorption coefficient	0.538 mm^{-1}	
F(000)	1044	

Crystal size	0.2 x 0.12 x 0.10 mm^3
Theta range for data collection	2.339 to 29.593°
Index ranges	-17<=h<=17, -18<=k<=18, -19<=l<=19
Reflections collected	101540
Independent reflections	11988 [R(int) = 0.0436]
Completeness to theta = 25.242°	99.9 %
Absorption correction	Semi-empirical from equivalents
Max. and min. transmission	0.1402 and 0.1062
Refinement method	Full-matrix least-squares on F^2
Data / restraints / parameters	11988 / 1089 / 814
Goodness-of-fit on F^2	1.0360
Final R indexes [I>2sigma(I)]	R1 = 0.0529, wR2 = 0.1311
R indexes (all data)	R1 = 0.0667, wR2 = 0.1404
Extinction coefficient	n/a
Largest diff. peak and hole	0.706 and -0.600 e.Å$^-$

Additional details for [Cp_2Co][$BAr^F{}_4$]: *This compound crystallised in the triclinic P-1 space group. Three CF_3 groups and the two Cp rings were modelled as disordered over two main domains and restrained to maintain sensible geometries. The hydrogen atoms were included using a riding model.*

2b

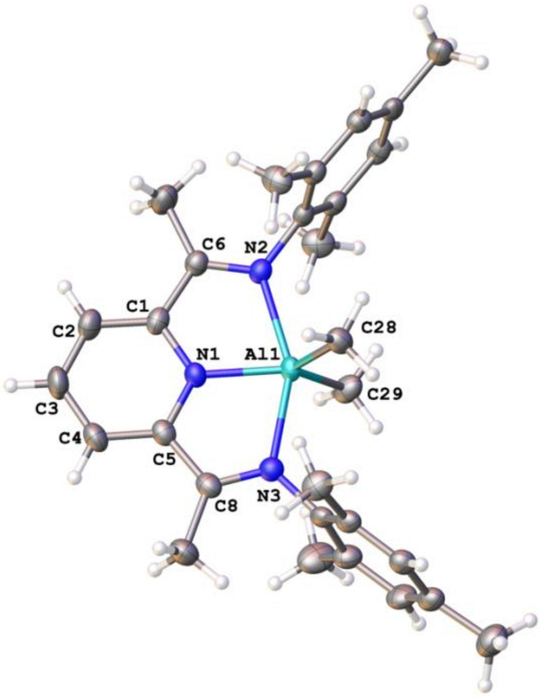

Figure S45. Molecular structure of **2b** with displacement ellipsoids at 50% probability. One disordered toluene molecule, solvent from crystallisation, has been omitted for clarity. Selected bond lengths (Å) and angles (°): Al(1)-N(1) 1.927(2), Al(1)-N(2) 2.166(3), Al(1)-N(3) 2.148(3), Al(1)-N(3) 2.148(3), Al(1)-C(28) 1.972(3), Al(1)-C(29) 1.972(3), N(1)-C(1) 1.381(4), N(1)-C(5) 1.373(4), N(2)-C(6) 1.304(4), N(2)-C(10) 1.443(4), N(3)-C(8) 1.308(4), N(3)-C(19) 1.440(4), C(1)-C(2) 1.378(4), C(1)-C(6) 1.439(4), C(2)-C(3) 1.385(5), C(3)-C(4) 1.388(5), C(4)-C(5) 1.384(4), C(5)-C(8) 1.446(4), C(6)-C(7) 1.500(4); C(28)-Al(1)-C(29) 113.8(2), C(28)-Al(1)-N(1) 122.9(1), C(29)-Al(1)-N(1) 123.2(1), C(28)-Al(1)-N(2) 96.7(1), C(29)-Al(1)-N(2) 96.9(1), N(1)-Al(1)-N(2) 76.5(1), N(1)-Al(1)-N(3) 76.7(1), C(28)-Al(1)-N(3) 97.8(1), C(29)-Al(1)-N(3) 97.6(1), N(2)-Al(1)-N(3) 153.2(1), C(1)-N(1)-C(5) 118.9(3).

Table S5. Crystal data and structure refinement for **2b.**

Empirical formula	$C_{36}H_{45}AlN_3$	
Formula weight	546.73	
Temperature	150(1) K	
Wavelength	0.71073 Å	
Crystal system	Monoclinic	
Space group	P21/c	
Unit cell dimensions	a = 20.6006(10) Å	α= 90(7)°
	b = 8.2294(4) Å	β=117.597(2)°
	c = 21.2265(9) Å	γ = 90 (6)°
Volume	3189.1(3) Å^3	
Z	4	
Density (calculated)	1.139 Mg/m^3	
Absorption coefficient	0.092 mm^{-1}	
F(000)	1180	
Crystal size	0.14 x 0.12 x 0.10 mm^3	
Theta range for data collection	1.922 to 25.706°.	
Index ranges	-25<=h<=25, -10<=k<=10, -25<=l<=23	
Reflections collected	102652	
Independent reflections	5926 [R(int) = 0.1070]	
Completeness to theta = 25.242°	98.8 %	
Absorption correction	Semi-empirical from equivalents	
Max. and min. transmission	0.1341 and 0.1090	
Refinement method	Full-matrix least-squares on F^2	
Data / restraints / parameters	5926 / 394 / 437	
Goodness-of-fit on F^2	1.156	
Final R indexes [I>2sigma(I)]	R_1 = 0.0861, wR_2 = 0.1526	

R indexes (all data)	R_1 = 0.1122, wR_2 = 0.1620
Extinction coefficient	n/a
Largest diff. peak and hole	0.314 and -0.284 e.Å^{-3}

Computational Details

Geometries and energies of the aluminum complexes and metallocene redox couples were computed using the commercial software package Spartan'20.[3] For the calculation of EPR parameters, we relied in the software ORCA.[4]

SPARTAN calculations: Starting points for geometry optimizations were built using coordinates from X-ray diffraction studies, or, in cases in which experimental data was not available, by modification the closest characterized analog. Before optimization, the positions of the hydrogen atoms were optimized using molecular mechanics (MMFF), fixing the positions of heavy atoms. In Spartan, all calculations were performed with the pure m-GGA functional M06–L functional to avoid known issues arising from the mixing Hartree−Fock exchange fraction in hybrid-DFT methods (ref 3801 bdd).[5] Molecular geometries were optimized with the def2–SV(P) basis functions, and the CPCM implicit model solvent using dichloromethane (ε = 8.93) and van der Waals radii of Fe and Co ≈ 2.2 in the MCp_2 molecules. The energy gradient convergence criterion was tightened from Spartan's default value $3x10^{-4}$ to $5x10^{-5}$ erg·bohr^{-1}. All stable geometries were checked to have zero or one imaginary frequency. The accuracy of the optimization procedure was tested by comparing a set of critical bond distances and agles with those of the experimental X-ray diffraction structures (Table S5-S9). In cases when two approximately symmetrical distances exist, we compared the averages. Thermal Corrections (TC = G° – E(SCF)) were computed at 298.15 (25 ºC) and 1 atmosphere. Electronic (SCF) energies were then refined with a single point calculation at the M06-L/def2–SVPD level in the gas phase, with the SCF convergence criterion set to "HIGH", to include full polarization and diffuse functions. To calculate a solvent correction (SC), we performed an inexpensive additional gas phase single-point energy calculation on the optimized geometries at the M06–L/def2–SVPD level (*i.e.*, omitting the solvent in the single-point calculation), therefore SC = E^{SCF} (CPCM) – E^{SCF}(gas), at the M06–L/def2–SV(P)) level. In addition to reducing the computational cost, this procedure has the advantage of computing the solvent effect at a level of the theory similar to that used for the parametrization of the CPCM model. Alternatively, the solvent effect can be included

in the SVPD single-point calculation but, apart from adding a significant cost to the SP calculation, we did not observe any significant variation in the final result. Refined G^o values were calculated for each molecule as G^o = E^{SCF}(M06-L/def2–SVPD) + TC + SC. Reduction potentials relative to the ferrocene/ferricenium couple were calculated from the ΔG^o corresponding to the redox reactions of the oxidized form **1a$^+$-d$^+$** with ferrocene (Eq S1) to give the reduced component **2a-d** plus ferricinium, and applying Faraday's equivalent relationship, ΔG^o = -*n*FE^o (with *n* =1, Eq. 2). Similarly, we computed ΔG^o and ΔE^o for the reactions of **1** with cobaltocene (Eq 2), which as a benchmark for this procedure, the potential computed for the cobalticinium/cobaltocene redox couple is -1.36 V, *versus* the experimental -1.33 V.[6] Similarly, energy balances for the analogous reactions with $CoCp_2$ enabled us to compute the expected potentials for the chemical reduction with cobaltocene.

$$\mathbf{1}^+ + FeCp_2 \rightarrow \mathbf{2} + FeCp_2^+ \quad (1)$$

$$\Delta E^o_{\mathbf{1+/2}} (vs.\ Fc^+/Fc) = -\Delta G^o(1)\ (\text{in kcal}\cdot\text{mol}^{-1}) \times 0{,}04336 \quad (2)$$

ORCA calculations: For the EPR calculations, the above geometries were re-optimized using the BP86 functional, def2-SVP basis set and def2/JK auxiliary (Coulomb and exchange) set. Single-point computations were then carried out to obtain the theoretical hyperfine coupling constants. In our hands, semi-local approaches —such as TPSS, M06L or SCAN— yielded values of hyperfine constants much larger than 20 MHz, specifically at the pyridine atoms H1 and N, which were inconsistent with the shape of the observed spectra. When resorting to hybrid functionals, however, the hyperfine coupling values obtained from DFT computations are consistent with those obtained by our fits and compare satisfactorily with previously published data (Scott *et al* for complex **2a**, **Table S9**). For the final single point calculations we selected the hybrid PW6B95 hybrid functional.[7] In these, the epr-II basis set,[8] was applied to 1st and 2nd row elements, whereas Ahlrichs' ma-TZVP set[9] was used for aluminium. Dispersion correction profited on the extended D3 coefficient model.[10] The Conductor-like Polarization Continuum Model (CPCM, toluene)[11] was applied to account for solvation effects. Final g and hyperfine coupling constants for complexes **2a-d** are collected in Table S1-S4) The calculated spectral parameters were fed into the EasySpin software (see EPR section in the "General Methods and Instrumentation" section) to check the consistency of results with

experimental data. For this purpose, isotropic values were derived, and data from equivalent atoms were averaged.

Table S6. DFT (M06-L) SCF energies a Gº for all species (Kcal·mol[-1]).

Comp.	**Geometry Optimization** M06L/SV(P)-CPCM (dichloromethane) ESCF(SV(P), solv)	Gº(SV(P), solv)	**Single point (gas)** M06L(SV(P) ESCF(SV(P), gas)	Thermal Correction, TC[a]	Solvent Correction, SC[b]	Single point (gas) M06L/SVPD ESCF(SVPD, gas)	Estimated[c] Gº(SVPD, solv)
**1a**[+]	-1109711,101	-1109269,010	-1109672,564	442,091	-38,537	-1109834,166	-1109391,632
**2a**	-1109793,810	-1109354,337	-1109785,608	439,473	-8,202	-1109917,894	-1109478,246
**1b**[+]	-961846,988	-961511,217	-961808,240	335,771	-38,748	-961902,607	-961605,584
**2b**	-961931,354	-961598,285	-961923,049	333,069	-8,305	-962018,817	-961694,053
**1c**[+]	-1158996,549	-1158518,439	-1158958,939	478,110	-37,610	-1159090,468	-1158649,968
**2c**	-1159079,401	-1158605,821	-1159071,401	473,580	-8,000	-1159204,022	-1158738,442
**1d**[+]	-1011131,529	-1010760,046	-1011093,884	371,483	-37,645	-1011235,223	-1010863,591
**2d**	-1011214,416	-1010846,681	-1011206,365	367,735	-8,051	-1011318,874	-1010950,952
**[Fc]**	-1035574,626	-1035489,734	-1035571,736	84,892	-2,890	-1035603,117	-1035521,115
**[Fc]**[+]	-1035460,382	-1035375,551	-1035417,549	84,831	-42,833	-1035449,035	-1035407,037
**[Cc]**	-1110257,807	-1110175,753	-1110254,782	82,054	-3,025	-1110286,006	-1110206,977
**[Cc]**[+]	-1110179,076	-1110092,991	-1110136,074	86,085	-43,002	-1110167,416	-1110124,333

a) TC (Thermal Correction) = Gº(SV(P), solv)-ESCF(SV(P), solv). b) SC (Solvent Correction) = ESCF(SV(P), solv) - ESCF(SV(P), gas). c) Estimated Gº(SVPD, solv) ≈ ESCF(SVPD, gas) + TC + SC.

Table S7. Comparison of Experimental (**Exp**, X-ray diffraction) and calculated (**Calc**, M06-L/SV(P), solvent), bond distances (Å) and angles (deg) for molecules **1**[+] and **2** and **RE**, relative errors.[a] Approximately symmetrical distances (e. g., *imine* N=C) are given as averages.

**Molecule**	**1a**[+]			**1c**[+]			**1d**[+]			**2a**			**2b**			**2c**		
**Distance/Angle**	**Exp**	**Calc**	**RE**	**Exp**	**Calc**	**RE%**	**Exp**	**Calc**	**RE%**	**Exp**	**Calc**	**RE%**	**Exp**	**Calc**	**RE**	**Exp**	**Calc**	**RE%**
C=N(im)	1.283	1.29	0.78	1.281	1.29	1.01	1.284	1.29	1.80	1.314	1.31	0.00	1.306	1.31	0.54	1.319	1.32	0,15
NC-C(Py)	1.492	1.48	0.87	1.483	1.47	0.47	1.484	1.48	1.07	1.421	1.45	1.76	1.443	1.45	0.17	1.453	1.44	0,58
N(Py)-αC(Py)	1.339	1.34	0.07	1.342	1.34	0.07	1.344	1.34	0.19	1.377	1.38	0.15	1.377	1.38	0.07	1.367	1.37	0.29
αC(Py)-βC(Py)	1.378	1.40	1.01	1.382	1.38	1.01	1.393	1.40	0.25	1.373	1.39	1.31	1.381	1.40	1.01	1.381	1.39	0.80
βC(Py)-γC(Py)	1.383	1.40	0.98	1.383	1.38	0.29	1.383	1.39	0.83	1.388	1.40	0.94	1.387	1.40	1.12	1.387	1.40	1.01
Al-N(im)	2.152	2.15	0.14	2.169	2.15	0.78	2.184	2.14	1.92	2.169	2.15	0.78	2.157	2.17	0.60	2.181	2.16	0.89
Al-N(Py)	2.011	2.01	0.07	1.997	2.02	1.25	2.019	2.02	0.20	1.997	2.02	1.25	1.927	1.95	1.40	1.958	1.96	0.15
Al-C	1.959	1.99	2.05	1.973	2.00	1.57	1.975	2.00	1.27	2.007	1.98	1.40	1.972	1.99	0.86	1.984	2.01	1.11
Al-C'	1.945	1.97	1.98	1.968	1.99	0.97	1.965	1.99	1.32	1.982	1.98	0.10	1.972	1.99	0.86	1.972	2.01	1.67
Ave. Rel. Err.%			**0.86**			**0.83**			**0.80**			**0.85**			**0.74**			**0.74**
N(im)-Al-N(im)	144.32	144.3	0.01	144.5	146.82	1.60	148.23	148.4	0.08	152.90	153.1	0.11	153.22	152.2	0.70	145.36	148.6	
Largest N(Py)-Al-C	142.50	149.1	4.65	145.7	149.26	2.45	137.18	145.7	6.20	126.10	126.2	0.08	123.22	125.1	1.53	142.72	145.2	
$\tau 5$ index[b]	0.03	0.08	--	0.07	0.04	0.02	0.18	0.04	--	0.45	0.45	--	0.50	0.45	--	0.04	0.06	--

a: Relative errors (**RE**), $100 \times \sqrt{(calc - exp)^2}/exp.$). **b**, Pentacoordinate geometry index, τ^5, (N(im)-Al-N(im) – largest N(Py)-Al-C)/60 (see Ref 32, main text)

Table S8. Optimized molecular geometries (M06-L/def2-SV(P)/CPCM) and atomic coordinates

1a^{+} S=0

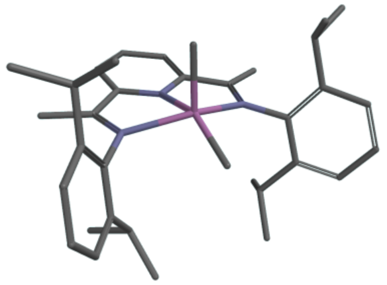

88

C	-1.882015	1.303575	-4.974611
C	-2.413602	0.320982	-4.140064
C	-1.630203	-0.298680	-3.163820
C	-2.184331	-1.410243	-2.293171
C	-1.859352	-2.778610	-2.893732
C	-3.679003	-1.291195	-2.030210
C	-3.868207	-1.009962	1.848105
H	-2.515641	1.781851	-5.730141
H	-3.464105	0.032353	-4.254068
H	-3.819863	0.570558	3.897591
H	-2.339867	-2.894860	-3.882764
H	-2.228195	-3.591135	-2.242212
H	-4.274272	-1.472555	-2.943217
H	-3.954259	-0.294720	-1.640532
H	-3.996499	-2.045095	-1.289365
H	-4.137911	-1.843326	1.176850
H	-4.099417	-0.064319	1.325771
H	-4.533994	-1.081792	2.726935
Al	0.396338	0.287807	0.001851
C	1.910363	-2.004558	-0.965795
C	2.892847	-2.995095	-0.901756
C	3.329484	-3.417776	0.353640
C	2.782009	-2.854117	1.505929
C	1.803646	-1.867629	1.363843
C	1.326139	-1.445529	-2.204507
C	1.733123	-1.989371	-3.522495
C	1.114524	-1.168310	2.470297
C	1.398019	-1.551503	3.874325
C	-0.277337	0.096764	-3.055803
C	0.284654	1.080954	-3.889417
C	-0.548574	1.674949	-4.846920
C	1.718623	1.550212	-3.752318
C	2.492396	1.426056	-5.062002
C	1.761959	2.982391	-3.222557
C	-0.539345	0.465165	2.989754
C	-1.903915	0.097236	3.021999
C	-2.758133	0.834555	3.845308
C	-2.283033	1.904481	4.602816
C	-0.936694	2.246231	4.552367
C	-0.033655	1.535364	3.750502
C	-2.400031	-1.103075	2.238760
C	-2.137231	-2.397307	3.009990
C	1.414426	1.975947	3.686625

2a S=1/2

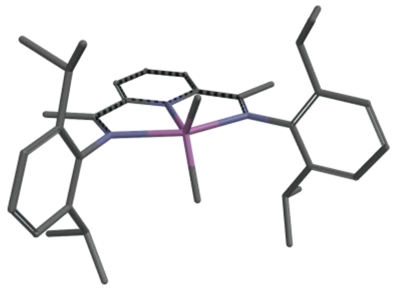

88

Al	0.018310	0.001111	-0.007466
C	-0.513453	2.935201	3.287143
C	-0.367866	1.795638	2.332682
C	-0.493757	0.434978	2.805491
C	-0.749795	0.077538	4.125427
C	-0.856848	-1.274774	4.473969
C	-0.702552	-2.243052	3.473782
C	-0.446879	-1.851272	2.163681
C	-0.272776	-2.761835	1.053799
C	-0.367756	-4.235938	1.273887
C	0.311427	5.611599	-0.917324
C	-0.944984	5.024127	-0.800123
C	-1.120749	3.801840	-0.140864
C	0.016356	3.167508	0.415076
C	-2.518099	3.228949	0.011170
C	-3.273659	3.930083	1.140685
C	-3.337736	3.299897	-1.274267
C	1.460679	-3.302926	-1.673310
C	0.543801	-4.279182	-3.707000
C	-0.736372	-3.895365	-3.317929
C	-0.960314	-3.212429	-2.116635
C	0.152614	-2.917693	-1.292715
C	-2.379818	-2.864689	-1.707109
C	-3.208400	-2.282468	-2.849360
C	-3.094610	-4.086316	-1.128291
C	-1.337006	0.363076	-1.404105
H	-0.401645	3.908623	2.784798
H	0.237871	2.877574	4.095088
H	-1.499847	2.916226	3.784001
H	-0.782525	-3.307513	3.716952
H	-0.213070	-4.801535	0.341856
H	-1.354562	-4.514964	1.684737
H	0.379822	-4.578581	2.011492
H	0.426781	6.569865	-1.437316
H	-1.818719	5.532184	-1.226382
H	-2.414396	2.163432	0.285333
H	-2.745306	3.860398	2.106147
H	-4.278303	3.488930	1.275109
H	-3.408996	5.004586	0.914016
H	-2.796906	2.878057	-2.138806
H	-3.618569	4.338897	-1.526777
H	-4.279242	2.732911	-1.159432
H	0.696858	-4.813483	-4.652043

C	2.066172	2.023376	5.065807
C	1.525675	3.325384	2.979150
C	2.082518	1.352212	0.009867
C	-1.313488	1.251272	-0.139354
H	3.313226	-3.425610	-1.814991
H	4.100818	-4.189918	0.434004
H	3.114596	-3.173489	2.497605
H	-0.137486	2.452384	-5.502628
H	2.225732	0.909638	-3.007961
H	-1.669510	-1.353865	-1.311923
H	-2.971215	2.475012	5.236809
H	-0.571048	3.093302	5.145582
H	-1.809084	-1.156038	1.300798
H	1.977165	1.240974	3.082612
H	2.078420	2.088612	-5.843669
H	2.474675	0.394183	-5.455487
H	3.548862	1.713748	-4.916278
H	2.801866	3.297466	-3.021110
H	1.188856	3.085925	-2.284135
H	1.333635	3.690148	-3.956398
H	-0.775058	-2.932334	-3.035480
H	-2.696935	-2.404320	3.963583
H	-1.069283	-2.534948	3.255192
H	-2.459859	-3.276421	2.423673
H	1.598206	2.787655	5.712472
H	3.136585	2.281647	4.978607
H	1.994384	1.053470	5.589437
H	1.042523	3.303500	1.986288
H	2.583620	3.610751	2.835721
H	1.040019	4.123762	3.570486
H	1.905485	2.439270	-0.089346
H	2.751268	1.051961	-0.820648
H	2.654086	1.198314	0.946133
H	-1.419321	1.949109	0.715872
H	-2.243678	0.654269	-0.176881
H	-1.302464	1.876412	-1.055348
H	1.099288	-1.608117	-4.337686
H	1.706661	-3.092492	-3.527195
H	2.779979	-1.698373	-3.734295
H	0.697327	-1.078454	4.579095
H	2.424407	-1.236405	4.143757
H	1.360447	-2.646421	4.005699
N	1.397057	-1.475151	0.150847
N	0.479131	-0.490897	-1.999920
N	0.295873	-0.247169	2.079854

1b^{+} S=0

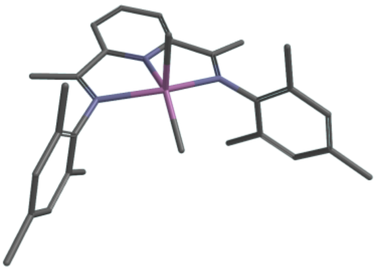

70

Al	-0.022521	-0.050094	0.153159
C	-0.531736	2.896750	3.440121
C	-0.393704	1.740281	2.506950

H	-1.590477	-4.138859	-3.961699
H	-2.319680	-2.099997	-0.911412
H	-2.693219	-1.449967	-3.358573
H	-4.170136	-1.896284	-2.465832
H	-3.451517	-3.045029	-3.611965
H	-2.561167	-4.516265	-0.264174
H	-3.187135	-4.883541	-1.889892
H	-4.115633	-3.823547	-0.795975
H	-1.144388	-0.313290	-2.261563
H	-1.185200	1.395623	-1.779382
H	-2.411350	0.266871	-1.153725
N	-0.127356	1.904222	1.045878
N	-0.341523	-0.521498	1.828925
N	-0.039029	-2.175975	-0.098074
H	-0.867227	0.856148	4.886040
H	-1.058341	-1.569226	5.508465
C	1.301107	3.751855	0.304027
C	1.419774	4.975120	-0.366264
C	2.528624	3.124021	0.938479
C	2.865516	3.805416	2.265318
C	3.752268	3.147824	0.026407
C	1.627541	-3.983802	-2.885151
C	2.663883	-3.053714	-0.782403
C	3.884015	-2.550791	-1.548978
C	3.034277	-4.315438	-0.001700
C	1.817580	0.259370	-0.790425
H	2.407637	5.444545	-0.450645
H	2.288996	2.066681	1.153313
H	2.026928	3.770955	2.980622
H	3.120943	4.870094	2.105786
H	3.736381	3.322538	2.745352
H	3.534637	2.742809	-0.976853
H	4.566749	2.541672	0.462662
H	4.148979	4.171531	-0.103143
H	2.634246	-4.296925	-3.188106
H	2.381203	-2.274977	-0.050742
H	3.646760	-1.686670	-2.193118
H	4.320261	-3.338783	-2.189976
H	4.675354	-2.236144	-0.844805
H	2.198473	-4.691283	0.611757
H	3.887161	-4.123130	0.674758
H	3.331942	-5.129037	-0.689955
H	1.920126	-0.415782	-1.664071
H	2.709675	0.099712	-0.153703
H	1.883337	1.293736	-1.184552

2b S=1/2

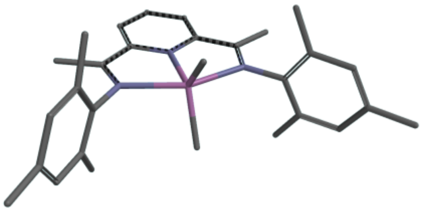

70

C	-1.745665	1.246809	-4.951533
C	-2.134274	0.087288	-4.271300
C	-1.316022	-0.525248	-3.317731

C	-0.517521	0.380400	2.983438	C	-1.754199	-1.768498	-2.605352
C	-0.769034	0.022609	4.303435	H	-3.111972	-0.362542	-4.488557
C	-0.873296	-1.331120	4.651540	H	-3.499067	0.221966	4.188919
C	-0.720258	-2.298063	3.649903	Al	0.667898	0.239086	0.014791
C	-0.468898	-1.904154	2.339568	C	1.940672	-2.193593	-0.954257
C	-0.296007	-2.808768	1.226123	C	2.790220	-3.299769	-0.889476
C	-0.382175	-4.286498	1.415485	C	3.165604	-3.777050	0.366133
C	0.298804	5.496091	-0.862945	C	2.681971	-3.157297	1.518161
C	-0.965034	4.926466	-0.672529	C	1.835996	-2.055405	1.376326
C	-1.148166	3.743860	0.052392	C	1.400198	-1.590403	-2.192615
C	-0.014693	3.098868	0.586205	C	1.643391	-2.252808	-3.496507
C	-2.524185	3.222492	0.330602	C	1.192410	-1.311218	2.481884
C	1.423624	-3.336058	-1.497061	C	1.314340	-1.812474	3.871901
C	0.529381	-4.219795	-3.599639	C	-0.068084	0.064312	-3.041498
C	-0.755865	-3.881356	-3.162649	C	0.360218	1.228137	-3.698568
C	-0.985023	-3.260762	-1.929423	C	-0.491512	1.792121	-4.653870
C	0.122800	-2.948758	-1.116834	C	1.686197	1.843259	-3.380923
C	-2.380384	-3.013563	-1.445141	C	-0.325441	0.435868	2.995590
C	-1.432948	0.308731	-1.203079	C	-1.607627	-0.100375	3.218817
H	-0.415708	3.856383	2.912048	C	-2.494995	0.630241	4.013858
H	0.225103	2.855556	4.244110	C	-2.142295	1.856165	4.589866
H	-1.519324	2.896497	3.935387	C	-0.852486	2.344331	4.353298
H	-0.797628	-3.363693	3.890214	C	0.070228	1.660597	3.554507
H	-0.213299	-4.824392	0.469131	C	-2.005993	-1.413454	2.617166
H	-1.373090	-4.583160	1.804229	C	1.435554	2.216029	3.299258
H	0.364109	-4.641623	2.148589	C	2.399459	1.218274	0.036329
H	-1.848042	5.424628	-1.095810	C	-1.095496	1.103285	-0.119602
H	-2.564976	2.121312	0.322683	H	3.147583	-3.783469	-1.802994
H	-1.619151	-4.116641	-3.800119	H	3.831017	-4.642120	0.447334
H	-2.468908	-2.068502	-0.885333	H	2.954004	-3.528657	2.510257
H	-1.293477	-0.361663	-2.076471	H	-0.157909	2.693900	-5.184006
H	-1.328274	1.345819	-1.584336	H	2.503181	1.098528	-3.384755
H	-2.486826	0.194977	-0.882359	H	-1.632910	-1.684840	-1.508844
N	-0.157959	1.841433	1.220485	H	-0.546247	3.296220	4.806740
N	-0.366673	-0.573599	2.004231	H	-1.786084	-1.458759	1.533808
N	-0.068263	-2.213729	0.078117	H	2.228106	1.459895	3.447337
H	-0.885139	0.801799	5.064016	H	2.281479	2.316950	-0.031382
H	-1.071508	-1.626695	5.686386	H	3.042473	0.905661	-0.809259
C	1.263589	3.680275	0.469205	H	2.963198	1.006621	0.965561
C	1.394603	4.864509	-0.264246	H	-1.265696	1.786195	0.736070
C	2.437594	3.091000	1.188225	H	-1.959235	0.410864	-0.147170
C	1.600653	-3.955651	-2.738724	H	-1.156686	1.717164	-1.040090
C	2.574638	-3.164824	-0.554547	H	1.156214	-1.716874	-4.324715
C	1.779445	0.221011	-0.642521	H	1.283928	-3.297255	-3.481750
H	2.392526	5.313662	-0.361650	H	2.729656	-2.299544	-3.695957
H	2.424372	1.989068	1.179285	H	0.759108	-1.183406	4.583571
H	2.615786	-4.250542	-3.038034	H	2.377820	-1.832312	4.172551
H	2.543039	-2.198250	-0.025831	H	0.948603	-2.852094	3.948140
H	1.908919	-0.452983	-1.514670	N	1.494017	-1.605581	0.162541
H	2.655381	0.059490	0.015579	N	0.715226	-0.511532	-1.999636
H	1.862432	1.257097	-1.031609	N	0.534386	-0.266061	2.101914
H	-3.258498	3.605203	-0.398086	H	-2.812927	-1.992561	-2.813030
H	-2.869563	3.540842	1.334332	H	-1.169711	-2.655439	-2.916973
H	3.392602	3.433382	0.755390	H	1.943351	2.634892	-4.103127
H	2.438505	3.394924	2.253927	H	1.686747	2.301021	-2.373920
H	2.563175	-3.948607	0.228651	H	1.650215	3.069224	3.962900
H	3.542199	-3.245547	-1.077727	H	1.537071	2.574086	2.257600
H	-2.705063	-3.817491	-0.754994	H	-3.083162	-1.602118	2.752529
H	-3.102099	-2.994893	-2.279080	H	-1.468105	-2.263185	3.079833
C	0.752987	-4.836357	-4.947110	C	-3.123321	2.633629	5.412401

H	1.627616	-5.509350	-4.951061
H	0.943783	-4.065569	-5.718342
H	-0.125184	-5.412359	-5.285622
C	0.473857	6.735480	-1.686977
H	1.339173	7.332587	-1.351620
H	-0.421485	7.379696	-1.654752
H	0.650887	6.491931	-2.752207

1c^{+} S=0

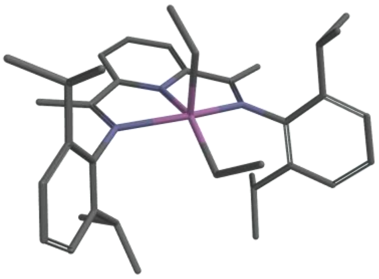

94

H	3.030821	-2.662752	1.716098
C	-0.258089	-2.881982	0.569250
C	-0.667138	-4.203167	0.373059
C	-1.221573	-4.554387	-0.856032
C	-1.360699	-3.587534	-1.851198
C	-0.929508	-2.287079	-1.584434
C	0.618179	-3.271441	2.937906
C	-1.516420	-1.374013	-3.900543
H	-0.560828	-4.942867	1.171476
H	-1.552778	-5.581472	-1.036959
H	-1.804847	-3.839193	-2.818270
H	1.171703	-4.160023	2.588556
H	-0.331243	-3.642865	3.368220
H	-2.595785	-1.616761	-3.871682
H	-1.014077	-2.236781	-4.370513
C	2.582245	-0.098233	2.662209
C	3.260153	0.563105	3.688632
C	3.287900	-0.502266	1.381707
C	4.394245	0.459305	0.969799
C	3.824264	-1.930103	1.483708
C	3.270001	1.384678	-2.862130
H	4.318684	0.815304	3.561624
H	2.531821	-0.494335	0.569258
H	4.037973	1.504527	0.935829
H	4.772400	0.197781	-0.033781
H	5.257881	0.417286	1.657642
H	4.585488	-2.005214	2.282267
H	4.298515	-2.241528	0.535669
H	3.585024	1.841359	-3.817611
H	4.071371	0.686574	-2.563790
H	3.222000	2.188940	-2.105952
H	2.886716	-1.084517	-3.947016
C	2.611443	0.910225	4.873104
H	3.160293	1.429848	5.666714
Al	-0.254620	-0.014615	0.104579
C	0.330550	-2.348702	1.812840
C	-1.018668	-1.150744	-2.521378
C	1.220307	-0.418326	2.867342
C	-0.542271	1.164094	-2.801820
C	-1.682313	1.940926	-3.084768

H	-2.620005	3.248697	6.177280
H	-3.846748	1.973793	5.920295
H	-3.712893	3.327783	4.783801
C	-2.653737	1.900018	-5.947629
H	-2.087800	2.393170	-6.756067
H	-3.273758	2.683439	-5.471916
H	-3.349675	1.176426	-6.404257

2c S=1/2

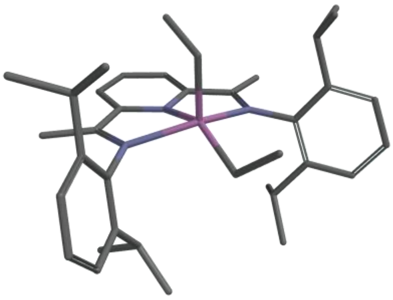

94

H	3.042471	-2.671274	1.771810
C	-0.191585	-2.883908	0.570703
C	-0.523212	-4.213405	0.337259
C	-1.047062	-4.585796	-0.910809
C	-1.232033	-3.604962	-1.888685
C	-0.885804	-2.281385	-1.615825
C	0.653142	-3.283616	2.948301
C	-1.540468	-1.379037	-3.913340
H	-0.386263	-4.959671	1.126467
H	-1.314627	-5.627883	-1.111616
H	-1.655841	-3.867667	-2.863708
H	1.217800	-4.170490	2.613721
H	-0.279334	-3.659920	3.410115
H	-2.628883	-1.581178	-3.905773
H	-1.063235	-2.243453	-4.405657
C	2.566517	-0.110809	2.705526
C	3.246228	0.546014	3.734346
C	3.277529	-0.513561	1.427178
C	4.373205	0.458073	1.008770
C	3.830424	-1.934493	1.535126
C	3.237817	1.337739	-2.908928
H	4.307781	0.790155	3.610823
H	2.518299	-0.518570	0.619398
H	4.003620	1.498788	0.968726
H	4.753377	0.196726	0.005220
H	5.239326	0.432341	1.695168
H	4.594684	-1.999099	2.332507
H	4.306431	-2.247214	0.587756
H	3.562660	1.795734	-3.861085
H	4.033246	0.633277	-2.607222
H	3.192572	2.140202	-2.150177
H	2.838974	-1.124735	-4.004868
C	2.597570	0.898656	4.917694
H	3.147601	1.412689	5.714659
Al	-0.312404	-0.035559	0.090184
C	0.342342	-2.365454	1.814255
C	-1.032509	-1.174341	-2.525586
C	1.197441	-0.424181	2.893808
C	-0.571722	1.157354	-2.814489
C	-1.694910	1.965565	-3.090818

C	-1.492165	3.143014	-3.779296	C	-1.498888	3.155827	-3.803674
C	-0.226305	3.558244	-4.175927	C	-0.233441	3.544275	-4.229894
C	0.884640	2.762960	-3.898795	C	0.864965	2.728169	-3.962170
C	0.754258	1.552784	-3.212748	C	0.721894	1.526587	-3.262078
C	-3.083383	1.549233	-2.656207	C	-3.094935	1.598474	-2.638724
C	-3.689387	2.593539	-1.719045	C	-3.690394	2.670913	-1.727768
C	-3.998086	1.328317	-3.860301	C	-4.020789	1.347652	-3.828328
C	1.948161	0.640556	-2.996806	C	1.909729	0.604848	-3.050057
C	2.039094	-0.396525	-4.117181	C	1.996705	-0.428811	-4.173561
C	0.821365	1.651403	0.229298	C	0.822563	1.612754	0.208556
C	-0.041610	2.912366	0.179086	C	-0.006593	2.896251	0.176218
C	-2.130197	0.179580	0.782582	C	-2.159238	0.266603	0.812045
C	-3.005133	-1.048635	0.937857	C	-3.043121	-0.966675	0.928477
H	-1.327962	-0.851792	3.365070	H	-1.341132	-0.820098	3.374472
H	-2.362720	3.769826	-4.008407	H	-2.363167	3.795340	-4.024699
H	-0.101905	4.505554	-4.712741	H	-0.100191	4.483493	-4.779584
H	1.875561	3.090597	-4.231855	H	1.857265	3.033376	-4.314364
H	-3.025726	0.594164	-2.099734	H	-3.030361	0.659962	-2.056510
H	1.782103	0.083386	-2.052148	H	1.733541	0.047075	-2.109172
H	-1.496736	1.680346	3.612515	H	-1.518221	1.700363	3.688790
H	-3.027799	2.820099	-0.864799	H	-3.021364	2.916590	-0.884505
H	-4.656108	2.241311	-1.316419	H	-4.655594	2.335089	-1.306651
H	-3.880600	3.544019	-2.250430	H	-3.882663	3.608052	-2.282943
H	-4.142869	2.265345	-4.428546	H	-4.162168	2.268127	-4.425014
H	-4.995815	0.985978	-3.532075	H	-5.019831	1.022061	-3.485519
H	-3.596652	0.575414	-4.560314	H	-3.627821	0.570773	-4.506856
H	1.124851	-1.009568	-4.200886	H	1.076929	-1.033108	-4.257156
H	2.195797	0.096185	-5.094547	H	2.159615	0.064833	-5.150083
H	-0.568662	3.027164	-0.786973	H	-0.569609	3.009357	-0.770839
H	0.548953	3.839217	0.325728	H	0.602146	3.818237	0.288959
H	-0.824021	2.912269	0.962836	H	-0.761864	2.920314	0.987060
H	-3.199368	-1.547829	-0.033035	H	-3.212992	-1.446732	-0.057124
H	-3.999002	-0.824140	1.375250	H	-4.048661	-0.766790	1.356622
H	-2.548963	-1.812445	1.601927	H	-2.583701	-1.746726	1.570866
H	1.193168	-2.781735	3.738136	H	1.235491	-2.778310	3.734987
H	-1.377613	-0.486953	-4.536968	H	-1.373929	-0.489272	-4.541689
N	-0.381303	-1.967510	-0.402812	N	-0.348557	-1.933596	-0.400529
N	0.549768	-1.073206	1.790215	N	0.527088	-1.065814	1.822512
N	-0.635003	-0.023193	-2.017355	N	-0.677836	-0.008358	-2.017162
C	1.266362	0.603764	5.041651	C	1.248556	0.602334	5.077176
C	0.539141	-0.064162	4.046503	C	0.522532	-0.057482	4.075642
C	-0.935075	-0.339961	4.263524	C	-0.952342	-0.333024	4.288001
C	-1.175314	-1.256090	5.461677	C	-1.192825	-1.279994	5.462019
C	-1.713179	0.965491	4.426378	C	-1.730719	0.966743	4.486965
H	0.758314	0.891252	5.970439	H	0.737657	0.890189	6.005034
H	-0.633338	-2.213413	5.371723	H	-0.652987	-2.235559	5.343700
H	-2.250873	-1.485676	5.565532	H	-2.268682	-1.511078	5.564745
H	-0.849414	-0.777423	6.403255	H	-0.861500	-0.827918	6.415489
H	-1.460109	1.465495	5.379575	H	-1.475964	1.444321	5.451649
H	-2.801701	0.774536	4.431660	H	-2.819744	0.776322	4.490533
H	-2.620808	0.914036	0.114684	H	-2.666327	1.027226	0.183854
H	-2.030447	0.702019	1.754509	H	-2.064627	0.749113	1.806399
H	1.362512	1.641295	1.196746	H	1.391350	1.594946	1.161039
H	1.622184	1.726447	-0.531618	H	1.603582	1.671107	-0.576089

1d^{+} S=0 **2d** S=1/2

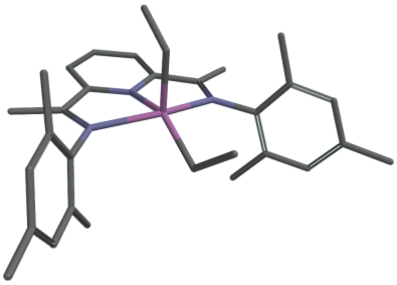

76

C	-0.210707	-3.071668	0.480279
C	-0.591085	-4.398102	0.267497
C	-1.154092	-4.742453	-0.960284
C	-1.327527	-3.763961	-1.938202
C	-0.924032	-2.457970	-1.655368
C	0.694123	-3.466156	2.844255
C	-1.515394	-1.522621	-3.965154
H	-0.453449	-5.148200	1.051451
H	-1.462583	-5.774323	-1.153914
H	-1.774648	-4.010326	-2.905451
H	1.301279	-4.317184	2.489540
H	-0.237298	-3.893870	3.259658
H	-2.555646	-1.896893	-3.960033
H	-0.905982	-2.292603	-4.470248
C	2.554126	-0.298865	2.695271
C	3.164105	0.429709	3.715032
C	3.334294	-0.807791	1.523048
H	4.234780	0.657827	3.630057
H	2.865982	-0.537565	0.557456
C	2.453481	0.869190	4.841951
Al	-0.213719	-0.192306	0.035700
C	0.377627	-2.544165	1.726860
C	-1.045797	-1.309848	-2.574980
C	1.178853	-0.594081	2.815616
C	-0.634889	1.012163	-2.830784
C	-1.743423	1.858636	-2.977913
C	-1.559228	3.059164	-3.677962
C	-0.327721	3.427903	-4.222163
C	0.743196	2.533034	-4.087067
C	0.613700	1.320242	-3.410178
C	-3.093110	1.526701	-2.418573
C	1.742714	0.336601	-3.358636
C	0.969191	1.408982	0.066094
C	0.145181	2.694823	0.146149
C	-2.045678	0.118550	0.774697
C	-3.021425	-1.043023	0.780003
H	-1.399805	-1.219562	3.340693
H	-2.421641	3.728193	-3.799159
H	1.712997	2.781400	-4.538230
H	-3.178222	0.479526	-2.085571
H	1.893365	-0.090686	-2.350418
H	-0.544097	2.813272	-0.712288
H	0.776972	3.605657	0.169607
H	-0.482620	2.724930	1.057519
H	-3.220014	-1.422396	-0.243259
H	-4.009731	-0.786760	1.212337
H	-2.644763	-1.906363	1.366512
H	1.232358	-2.954422	3.656106
H	-1.481935	-0.594330	-4.554768

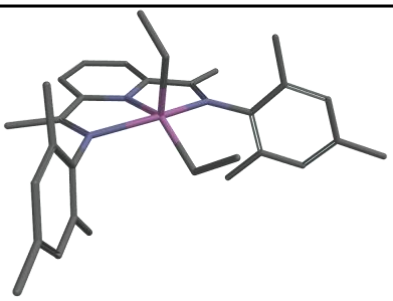

76

C	-0.138487	-3.076600	0.479849
C	-0.415227	-4.414733	0.213624
C	-0.936627	-4.777782	-1.035052
C	-1.169709	-3.785160	-1.992485
C	-0.877280	-2.455324	-1.693393
C	0.788920	-3.415752	2.867159
C	-1.501001	-1.459216	-3.992921
H	-0.234348	-5.180663	0.973470
H	-1.161413	-5.825496	-1.257698
H	-1.583752	-4.054936	-2.968581
H	0.714249	-4.489661	2.645326
H	0.156334	-3.206668	3.749890
H	-2.435398	-0.891509	-4.158467
H	-1.679081	-2.501377	-4.293713
C	2.525658	-0.322878	2.742902
C	3.144077	0.371889	3.781941
C	3.303955	-0.800259	1.555585
H	4.218036	0.590281	3.704689
H	2.841713	-0.481791	0.602365
C	2.437477	0.792593	4.918559
Al	-0.287627	-0.210034	0.043286
C	0.395997	-2.551788	1.717773
C	-1.049325	-1.327591	-2.578247
C	1.142885	-0.604698	2.839443
C	-0.670111	0.996285	-2.842867
C	-1.773499	1.849756	-3.013394
C	-1.596636	3.029775	-3.749732
C	-0.369180	3.383620	-4.312886
C	0.702534	2.494188	-4.152331
C	0.574795	1.300838	-3.439583
C	-3.118517	1.539163	-2.429431
C	1.717376	0.334938	-3.348021
C	0.912434	1.404102	0.085264
C	0.105966	2.702526	0.126837
C	-2.090988	0.207528	0.812609
C	-3.072901	-0.954363	0.843212
H	-1.435184	-1.171190	3.321912
H	-2.459626	3.696868	-3.883164
H	1.672907	2.734296	-4.608340
H	-3.185634	0.510336	-2.040684
H	1.844954	-0.071168	-2.328597
H	-0.573363	2.807114	-0.742334
H	0.738107	3.615601	0.140584
H	-0.537385	2.755918	1.027009
H	-3.283929	-1.339125	-0.175665
H	-4.058487	-0.710844	1.294691
H	-2.675092	-1.818501	1.414552
H	1.828054	-3.200951	3.176310
H	-0.755145	-1.028597	-4.685776

N	-0.371460	-2.143610	-0.474471
N	0.565233	-1.264658	1.718468
N	-0.705869	-0.178657	-2.049438
C	1.104566	0.526032	4.937827
C	0.441532	-0.213389	3.946560
C	-0.993894	-0.593951	4.151010
H	0.534878	0.832088	5.825406
H	-2.465113	0.969099	0.202049
H	-1.899756	0.509631	1.801996
H	1.633520	1.369381	0.951148
H	1.655826	1.468239	-0.800781
H	2.690766	0.798310	-3.678983
H	1.554448	-0.523008	-4.031409
H	-3.337005	2.166869	-1.550182
H	-3.883341	1.707829	-3.168260
H	-1.641200	0.297479	4.235595
H	-1.115181	-1.155882	5.095125
H	4.362847	-0.412296	1.524185
H	3.403375	-1.912759	1.533012
C	-0.147577	4.735726	-4.929377
H	0.394561	5.463210	-4.296470
H	-1.114324	5.194005	-5.195759
H	0.445967	4.622975	-5.853337
C	3.129525	1.682076	5.902516
H	4.119374	1.269361	6.164662
H	2.525845	1.737740	6.823326
H	3.304395	2.720150	5.562796

Fc^{+} (FeCp$_2$)$^{+}$ S=1/2

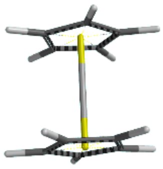

23

Fe	-0.000004	0.000002	0.000000
H	-0.734336	2.183490	-1.652949
C	-0.390062	1.146785	-1.695566
C	-1.226840	-0.000276	-1.718294
H	1.854108	1.357771	-1.624534
H	-2.318947	-0.000421	-1.703601
C	-0.389912	-1.147148	-1.696891
H	-0.734093	-2.183907	-1.654415
C	0.972576	-0.714207	-1.658515
H	1.853887	-1.358637	-1.625637
C	0.972640	0.713560	-1.657847
H	-1.853861	1.358672	1.625653
C	-0.972562	0.714226	1.658523
C	0.389935	1.147140	1.696886
H	-1.854134	-1.357735	1.624552
H	0.734137	2.183892	1.654407
C	1.226841	0.000252	1.718284
H	2.318947	0.000377	1.703581
C	0.390040	-1.146792	1.695564
H	0.734293	-2.183504	1.652944
C	-0.972653	-0.713541	1.657856
centr	-0.012320	-0.000257	-1.685423
centr	0.012320	0.000257	1.685423

N	-0.358650	-2.111608	-0.469148
N	0.526984	-1.243098	1.734961
N	-0.743655	-0.165772	-2.035583
C	1.082155	0.471239	4.996303
C	0.413669	-0.229644	3.980268
C	-1.032091	-0.583827	4.161865
H	0.511818	0.767196	5.887698
H	-2.530891	1.054160	0.246355
H	-1.943045	0.604826	1.839127
H	1.555996	1.374754	0.988312
H	1.624654	1.452770	-0.763829
H	-3.360686	2.219499	-1.591084
H	-3.917675	1.674897	-3.180329
H	1.560477	-0.541288	-4.007342
H	2.666463	0.804987	-3.655511
H	-1.178594	-1.173343	5.086009
H	-1.662463	0.318219	4.266794
H	3.350309	-1.905816	1.517085
H	4.341037	-0.426222	1.577497
C	3.123208	1.563944	6.005136
H	4.088326	1.106219	6.286501
H	2.501473	1.632241	6.913471
H	3.351339	2.598734	5.686868
C	-0.193487	4.671668	-5.058559
H	0.333257	5.427021	-4.444849
H	-1.162036	5.111431	-5.350260
H	0.410629	4.537853	-5.973235

Fc (FeCp$_2$) S=0

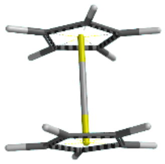

23

Fe	-0.000005	0.000001	0.000000
H	-0.713520	2.196254	-1.599922
C	-0.375654	1.156704	-1.624241
C	-1.215986	0.000050	-1.623929
H	1.868362	1.357282	-1.599452
H	-2.309070	0.000074	-1.599330
C	-0.375706	-1.156642	-1.624274
H	-0.713618	-2.196176	-1.599984
C	0.984020	-0.714828	-1.624474
H	1.868301	-1.357320	-1.599490
C	0.984052	0.714830	-1.624454
H	-1.868394	1.357191	1.599499
C	-0.984068	0.714761	1.624479
C	0.375627	1.156667	1.624271
H	-1.868268	-1.357410	1.599463
H	0.713468	2.196225	1.599979
C	1.215986	0.000033	1.623922
H	2.309071	0.000085	1.599318
C	0.375734	-1.156679	1.624240
H	0.713671	-2.196205	1.599919
C	-0.984002	-0.714898	1.624461
centr	0.000145	0.000023	-1.624274
centr	-0.000145	-0.000023	1.624274

Cc⁺ $(CoCp_2)^+$ S=0

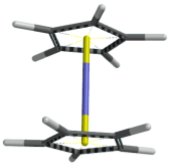

23

Co	0.000002	-0.000021	0.000006
H	-0.713012	-2.192542	-1.569811
C	-0.375221	-1.154832	-1.615842
C	0.982656	-0.714151	-1.615514
H	-2.305114	0.000883	-1.569353
H	1.865277	-1.355920	-1.568283
C	0.983203	0.713389	-1.615485
H	1.866319	1.354473	-1.568222
C	-0.374335	1.155110	-1.615798
H	-0.711321	2.193080	-1.569711
C	-1.213801	0.000462	-1.615770
H	0.712989	-2.192537	1.569828
C	0.375206	-1.154825	1.615848
C	-0.982668	-0.714126	1.615513
H	2.305115	0.000870	1.569371
H	-1.865292	-1.355889	1.568286
C	-0.983195	0.713415	1.615472
H	-1.866305	1.354505	1.568206
C	0.374351	1.155117	1.615786
H	0.711346	2.193083	1.569696
C	1.213802	0.000458	1.615774
centr	0.000500	-0.000004	-1.615682
centr	-0.000501	0.000008	1.615679

Cc $(CoCp_2)$ S=1/2

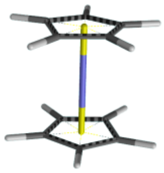

23

Co	0.000000	0.000000	0.000000
H	-1.606152	1.644620	-1.698683
C	-0.850777	0.854596	-1.711863
C	-1.091417	-0.534156	-1.732851
H	1.076315	2.032762	-1.659387
H	-2.066922	-1.026121	-1.720024
C	0.185621	-1.190787	-1.665825
H	0.339915	-2.273370	-1.637217
C	1.215632	-0.204205	-1.733160
H	2.291686	-0.390727	-1.709997
C	0.577801	1.059715	-1.682618
H	-2.291686	0.390727	1.709997
C	-1.215632	0.204205	1.733160
C	-0.185621	1.190787	1.665825
H	-1.076315	-2.032762	1.659387
H	-0.339915	2.273370	1.637217
C	1.091417	0.534156	1.732851
H	2.066922	1.026121	1.720024
C	0.850777	-0.854596	1.711863
H	1.606152	-1.644620	1.698683
C	-0.577801	-1.059715	1.682618
centr	0.007372	-0.002967	-1.705263
centr	-0.007372	0.002967	1.705263

Table S9. Comparison of computed EPR parameters for complex with literature data (Scott *et al*,[2] for **2a**) and **2b**, using different computational models.

	Scott *et al.* (**2a**)**Error! Bookmark not defined.**		This work (**2b**)				
**DFT Method** Functional/Basis<br>**Parameter** (No. of eq. nuclei)	**Experim. Data** (fitted)	B3-LYP def2-TZVP	**Experim. Data** (fitted)	B3-LYP EPRII/ def2-maTZVP	PW6B95 EPRII/ def2-maTZVP	BP86 def2-svp	PW6B95 IGLOIII
g_{iso}	2.0047	2.0031	2.0049	2.0033	2.0031	2.0029	2.11
A_{iso}^{Al}	13.39	20.90	16.43	-20.03	-19.53	-17.49	-21.59
A_{iso}^{1N}	15.45	9.90	12.16	10.23	9.32	19.59	10.48
A_{iso}^{2N} (2)	5.50	4.60	2.99	4.39	3.81	8.42	4.35
A_{iso}^{1H}	17.81	15.80	14.47	-17.56	-16.83	-13.80	-15.52
A_{iso}^{2H} (2)	4.94	4.50	5.13	4.56	3.93	1.83	3.68
A_{iso}^{6H} (6)	7.50	6.50	6.82	4.24	6.46	7.68	6.01
A_{iso}^{Me} (6)	<0.5	0.11	0.17	-0.12	-0.25	-0.35	-0.23

Table S10. Full listing of fitted (experimental) and calculated EPR parameters for complexes **2a-d** at the PW6B95/EPRII/def2-maTZVP level

	**2a**		**2b**		**2c**		**2d**	
**Parameter** (no. of nuclei)	**Exp.** (fitted)	**Calcd.**	**Exp.**	**Calcd.**	**Exp.** (fitted)	**Calcd.**	**Exp.** (fitted)	**Calcd.**
g_{iso}	2.0063	2.0031	2.0049	2.0031	2.0042	2.0031	2.0063	2.0031
A_{iso}^{Al}	-14.83	-19.33	16.43	-19.53	15.30	-19.78	15.25	-21.52
A_{iso}^{1N}	14.87	8.91	12.16	9.32	12.32	8.84	14.42	8.84
A_{iso}^{2N} (2)	4.99	4.21	2.99	3.81	5.18	4.43	4.94	4.35
A_{iso}^{1H}	16.04	-15.56	14.47	-16.83	13.28	-12.21	17.50	-13.78
A_{iso}^{2H} (2)	2.57	2.88	5.13	3.93	2.16	0.66	5.02	3.11
A_{iso}^{6H} (6)	4.99	6.88	6.82	6.46	6.55	7.92	7.45	7.92
A_{iso}^{Me} (6)	0.16	-0.34	0.17	-0.25	--	--	--	--
$A_{iso}^{CH_2(Et)}$ (4)	--	--	--	--	0.19	0.08	0.17	-0.076
$A_{iso}^{Me(Et)}$ (6)	--	--	--	--	<0.01	0.52	*n.a.*	0.56

References

[1] Stoll, S.; Schweiger, A. EasySpin, a comprehensive software package for spectral simulation and analysis in EPR. *Journal of Magnetic Resonance* **2006**, *178*, 42-55. DOI: https://doi.org/10.1016/j.jmr.2005.08.013

[2] Scott, J.; Gambarotta, S.; Korobkov, I.; Knijnenburg, Q.; de Bruin, B.; Budzelaar, P. H. M., Formation of a Paramagnetic Al Complex and Extrusion of Fe during the Reaction of (Diiminepyridine)Fe with AlR_3 (R = Me, Et). *Journal of the American Chemical Society* **2005,** *127* (49), 17204-17206. DOI: https://doi.org/10.1021/ja056135s

[3] Spartan'20. Wavefunction, Inc. Irvine, CA. (Version 1.1.2, 2021).

[4] a) ORCA Version 4.1.2. Freely available from https://orcaforum.kofo.mpg.de/app.php/dlext/?cat=8. b) Neese, F. The ORCA program system. *WIREs Computational Molecular Science* **2012**, *2*, 73-78. DOI: https://doi.org/10.1002/wcms.81. c) Neese, F. Software update: the ORCA program system, version 4.0. *WIREs Computational Molecular Science* **2018**, *8*, e1327. DOI: https://doi.org/10.1002/wcms.1327.

[5] Toma, M.; Kuvek, T.; Vrček, V. Ionization Energy and Reduction Potential in Ferrocene Derivatives: Comparison of Hybrid and Pure DFT Functionals. *J. Phys. Chem., A* **2020**, *124*, 8029-8039. DOI: https://doi.org/10.1021/acs.jpca.0c06663.

[6] Connelly, N. G.; Geiger, W. E. Chemical Redox Agents for Organometallic Chemistry. *Chem. Rev.* **1996**, *96*, 877-910. DOI: https://doi.org/10.1021/cr940053x.

[7] Zhao, Y.; Truhlar, D. G., Design of Density Functionals That Are Broadly Accurate for Thermochemistry, Thermochemical Kinetics, and Nonbonded Interactions. *J. Phys. Chem. A,* **2005,** *109* (25), 5656-5667. DOI: doi.org/10.1021/jp050536c

[8] Baroe, V., Structure, Magnetic Properties and Reactivities of Open-Shell Species From Density Functional and Self-Consistent Hybrid Methods. In *Recent Advances in Density Functional Methods*, pp 287-334. DOI: https://doi.org/10.1142/9789812830586_0008

[9] Weiend, F.; Ahlrichs, R., Balanced basis sets of split valence, triple zeta valence and quadruple zeta valence quality for H to Rn: Design and assessment of accuracy. *PhysChemChemPhys* **2005,** *7*, 3297-3305. DOI: https://doi.org/10.1039/B508541A

[10] Caldeweyher, E.; Bannwarth, C.; Grimme, S. Extension of the D3 dispersion coefficient model. *J. Chem. Phys.* **2017**, *147*. DOI: https://doi.org/10.1063/1.4993215 (accessed 6/9/2024).

[11] Barone, V.; Cossi, M.; Tomasi, J. Geometry optimization of molecular structures in solution by the polarizable continuum model. *J. Comput. Chem.* **1998**, *19*, 404-417. DOI: https://doi.org/10.1002/(SICI)1096-987X(199803)19:4<404::AID-JCC3>3.0.CO;2-W.
